# Characterization of the WRKY gene family in *Akebia trifoliata* and their response to *Colletotrichum acutatum*

**DOI:** 10.1186/s12870-022-03511-1

**Published:** 2022-03-14

**Authors:** Feng Wen, Xiaozhu Wu, Tongjian Li, Mingliang Jia, Liang Liao

**Affiliations:** 1 School of Pharmacy and Life Science, Jiujiang University, Jiujiang, China; 2 State Key Laboratory of Ecological Pest Control for Fujian and Taiwan Crops, College of Plant Protection, Fujian Agriculture and Forestry University, Fuzhou, China

**Keywords:** WRKY transcription factors, *Akebia trifoliata*, biotic stress, *Colletotrichum acutatum*

## Abstract

**Background:**

*Akebia trifoliata*, belonging to the Lardizabalaceae family, is a well-known Chinese traditional medicinal plant, susceptible to many diseases, such as anthracnose and powdery mildew. WRKY is one of the largest plant-specific transcription factor families and plays important roles in plant growth, development and stress response, especially in disease resistance. However, little was known about the numbers, characters, evolutionary relationship and expression of *WRKY* genes in *A. trifoliata* in response to plant disease due to lacking of *A. trifoliata* genome.

**Results:**

A total of 42 putative *AktWRKY* genes were identified based on the full-length transcriptome-sequencing data of *A. trifoliata*. Then 42 *AktWRKY* genes were divided into three major groups (Group I-III) based on the WRKY domains. Motif analysis showed members within same group shared a similar motif composition, implying a functional conservation. Tissue-specific expression analysis showed that *AktWRKY* genes could be detected in all tissues, while few *AktWRKY* genes were tissue specific. We further evaluated the expression of *AktWRKY* genes in three varieties in response to *Colletotrichum acutatum* by qRT-PCR. The expression patterns of *AktWRKY* genes were similar between C01 and susceptible variety I02, but distinctly different in resistant variety H05. In addition, it showed that more than 64 percentages of *AktWRKY* genes were differentially expressed during fungal infection in I02 and H05. Furthermore, Gene ontology (GO) analysis showed that *AktWRKY* genes were categorized into 26 functional groups under cellular components, molecular functions and biological processes, and a predicted protein interaction network was also constructed.

**Conclusions:**

Results of bioinformation analysis and expression patterns implied that AktWRKYs might play multiple function in response to biotic stresses. Our study could facilitate to further investigate the function and regulatory mechanism of the WRKY in *A. trifoliata* during pathogen response.

**Supplementary Information:**

The online version contains supplementary material available at 10.1186/s12870-022-03511-1.

## Introduction

The WRKY transcription factor family is one of the largest transcriptional regulatory gene families in plants, which can regulate downstream transcription through specifically recognizing and binding with the cognate *cis*-element W-box (TTGACT/C), which is usually located in the promoter region of genes related to growth, development and stress response [1–3].

Structurally, WRKY transcription factors contain one or two conserved WRKY domains, which consist of a signature sequence (WRKYGQK) along with a C_2_H_2_ or C_2_HC zinc-binding motif. Generally, WRKY transcription factors are classified into three major groups based on their structure. Group I WRKYs contain two WRKY domains at the N- and C-terminal, each of which is followed by a C_2_H_2_ zinc-finger, while Group II WRKYs have only one WRKY domain. Similarly, Group III also contains only one WRKY domain, but there is a C_2_HC zinc-finger motif at the C-terminal of the WRKY domain instead of C_2_H_2_ zinc-finger [4–7]. By inferring the results of a nuclear magnetic resonance solution structure of the C-terminal WRKY domain of AtWRKY4, the conserved WRKY domain is composed of a four-stranded beta-sheet with a zinc binding pocket, which is directly involved in DNA binding [8, 9]. Previous studies have shown that WRKY domain sequences can directly bind to W-box (C/T)TGAC(C/T) *cis*-regulatory element, which were found in the promoter region of the target genes [3, 10]. These (C/T)TGAC(C/T) sequence elements contain the invariant TGAC core that mediate transcriptional responses to biotic stresses. Thus, plant genes that contain TGAC core in the promoter regions are considered as defense-associated genes [5, 11]. Interactions between the WRKY domain and the W box have been demonstrated by numerous binding experiments, both in *vitro* and in *vivo*, and these interactions can also be regulated post-translationally, since these bindings can be inhibited by phosphatase and protein-kinase inhibitors [12, 13]. Previous studies have proven that WRKYs play essential roles in plant growth, development, and abiotic or biotic stresses responses [14–16]. For instance, AtWRKY12 could partly mediate the effect of GA3 in controlling flowering time and regulating the formation of pith secondary wall in Arabidopsis [17–19]. Wintersweet WRKY71 was proven to be involved in the regulation of flowering and leaf senescence in Arabidopsis [20]. Interestingly, WRKY transcription factors, although are transcription factors themselves, are also regulated by other WRKYs [21]. For example, WRKY18 was able to bind directly to different W-boxes in the WRKY53 promoter region, thereby repressing the expression of WRKY53. Thus, WRKY18 could act as a positive senescence regulator due to its repressing function on WRKY53 [22]. Furthermore, WRKYs have been found to be involved in various biotic and abiotic stress defense responses, such as viruses, bacterial pathogens, fungi, heat, drought [23–28]. For instance, PlWRKY65, as a disease resistance-related transcriptional activator, could exert a regulatory effect on JA and SA signals to enhances the resistance of *Paeonia lactiflora* to *Alternaria tenuissima* [29]. Additionally, it has been reported that VvWRKY30 overexpression lines had higher antioxidant activities and lower reactive oxygen species contents under salinity stress, thus enhancing their resistance to salt stress [30].

Since WRKY transcription factors play critical roles in plant development and stresses resistance, WRKYs have been identified genome-wide from various plant species with the development of high-throughput sequencing, including 74 WRKYs in Arabidopsis, 103 in rice, 86 in *Brachypodium distachyon*, 197 in soybean, and 54 in pineapple [5, 21, 31–34]. However, the numbers, characters, evolutionary relationships and expressions of *WRKY* genes in *Akebia trifoliata* (Thunb.) Koidz. were completely unknown. *A. trifoliata* belonging to the Lardizabalaceae family, was mainly distributed in the eastern part of Asia, which was a well-known Chinese traditional medicinal plant, as its antiphlogistic, antineoplastic and diuretic characters [35, 36]. The wild resources of *A. trifoliata* were on the verge of exhaustion because of overexploitation. Due to its medicinal and edible value, *A. trifoliata* has been developing as an artificial cultivation commercial crop in Hunan and Jiangxi province in China. However, the cultivated *A. trifoliata* seedlings were susceptible to disease, thus it is very important to protect them from pathogen. In this study, a total of 42 putative *AktWRKY* genes were identified based on the full-length transcriptome-sequencing data of *A. trifoliata*. Subsequently, the characters of *AktWRKYs* and their expression patterns in response to *Colletotrichum acutatum* have been further analyzed. Our results could provide the novel insight into protein structures, evolutionary relationships, and expression pattern of WRKYs in *A. trifoliata*, which could also facilitate to further investigate the biological functions of AktWRKYs under biotic stresses.

## Materials and methods

### Database search and identification of WRKY transcription factors

All 42 putative WRKY proteins were retrieved from full-length transcriptome sequencing data of *A. trifoliata* (unpublished data). Arabidopsis WRKY protein sequences were downloaded from the database of The Arabidopsis Information Resource (TAIR, https://www.arabidopsis.org/). The AktWRKY proteins were identified by blastp method using Geneious software with Arabidopsis WRKY proteins as query sequences. The identified AktWRKY proteins in *A. trifoliata* were rechecked and confirmed to avoid repetition and verify the reliability of our results: a) short AktWRKY sequences with an incomplete WRKY domain have been removed, b) all putative non-redundant sequences were assessed with UniProt and SMART (http://smart.embl-heidelberg.de/) analyses, respectively. The neighbor-joining (NJ) phylogenetic tree based on 42 putative WRKY proteins were constructed to classify the AktWRKYs. All putative WRKY gene family members in *A. trifoliata* were designed their names base on the homologs in Arabidopsis.

### Protein Motifs, Structure Analysis and Phylogenetic Analysis

The conserved motifs in the WRKY proteins were predicted using MEME suite (http://meme.sdsc.edu/meme/cgi-bin/meme.cgi). The parameters were set as follows: maximum number, 6; site distribution, any number of repetitions; minimum width, 10; and maximum width, 80. The graph was generated by TBtools v1.068. Subsequently, multiple alignment analyses of the WRKY domains sequence were performed by ClustalW (www.ebi.ac.uk/clustalw/). The phylogenetic tree based on the alignment of WRKY domains in rice, Arabidopsis, and *A. trifoliata* was constructed using MEGA X software with NJ method and following parameters: p-distance and pairwise deletion. Bootstrap analysis was performed with 1000 replicates.

### Plant materials and expression analysis

*A. trifoliata*, which is not a protected plant, was identified by Prof. Liao Liang according to Flora of China. The original samples (C01, I02, and H05) were obtained from Yuncheng City, Shanxi Province, Lu’an City, Anhui Province, and Xiangyang City, Hubei Province, respectively. The voucher specimens deposited in Jiujiang University (accession number JJU130C01, JJU130I02, and JJU130H05, respectively). The seedlings were planted in Mutong yard in Jiujiang University, Jiujiang, Jiangxi province, China. *A. trifoliata* tissues of buds, young leaves, mature leaves, stems, female flowers, and male flowers were collected from the Mutong yard. For pathogen infection analysis, *A. trifoliata* seedlings were sprayed with spores of *C. acutatum*. After infection for 6 h, the leaves were collected for genes expression analysis. Total RNA was extracted from plant tissues by using Trizol reagent (Invitrogen, USA) according to the manufacturer’s protocol. A RNase-free DNaseI (TaKaRa, Japan) was used to remove genomic DNA contamination. First-strand cDNA was synthesized using PrimeScript™ 1st Strand cDNA Synthesis Kit (TaKaRa, Japan) according to the manufacturer’s protocol. The expression of the all *AktWRKY* genes was assessed upon the qPCR result analysis. For tissue-specific analysis, the average of total Δ*C*Tvalue (Δ*C*T. average) was subtracted from all other Δ*C*T values to obtain second normal standardization, according to the previous method [37]. For phytopathogen infection analysis, the expression level of test genes was calculated with the 2 ^−ΔΔ*C*T^ method. The data were statistically analyzed using an OriginPro 7.5 software. Gene specific primers for quantitative real-time PCR are listed in Additional file 4. Public Arabidopsis expression datasets were obtained from the Gene Expression Omnibus (GEO) and the AtGenExpress Consortium (Arabidopsis eFP Browser).

### Gene ontology annotation and protein interaction analysis

To perform the functional classification, all putative *AktWRKY* genes were analyzed using Blast2GO basic software. And then, the results were performed by using an online tool WEGO (http://wego.genomics.org.cn/) to compare and plot Gene Ontology annotation results. Protein-protein interactions were predicted by the STRING 11.0 program (https://string-db.org/) based on an Arabidopsis association model with the confidence parameter set at a threshold of 0.35.

## Results and discussion

### Identification of WKRY transcription factors in *A. trifoliata*

**Fig. 1:**
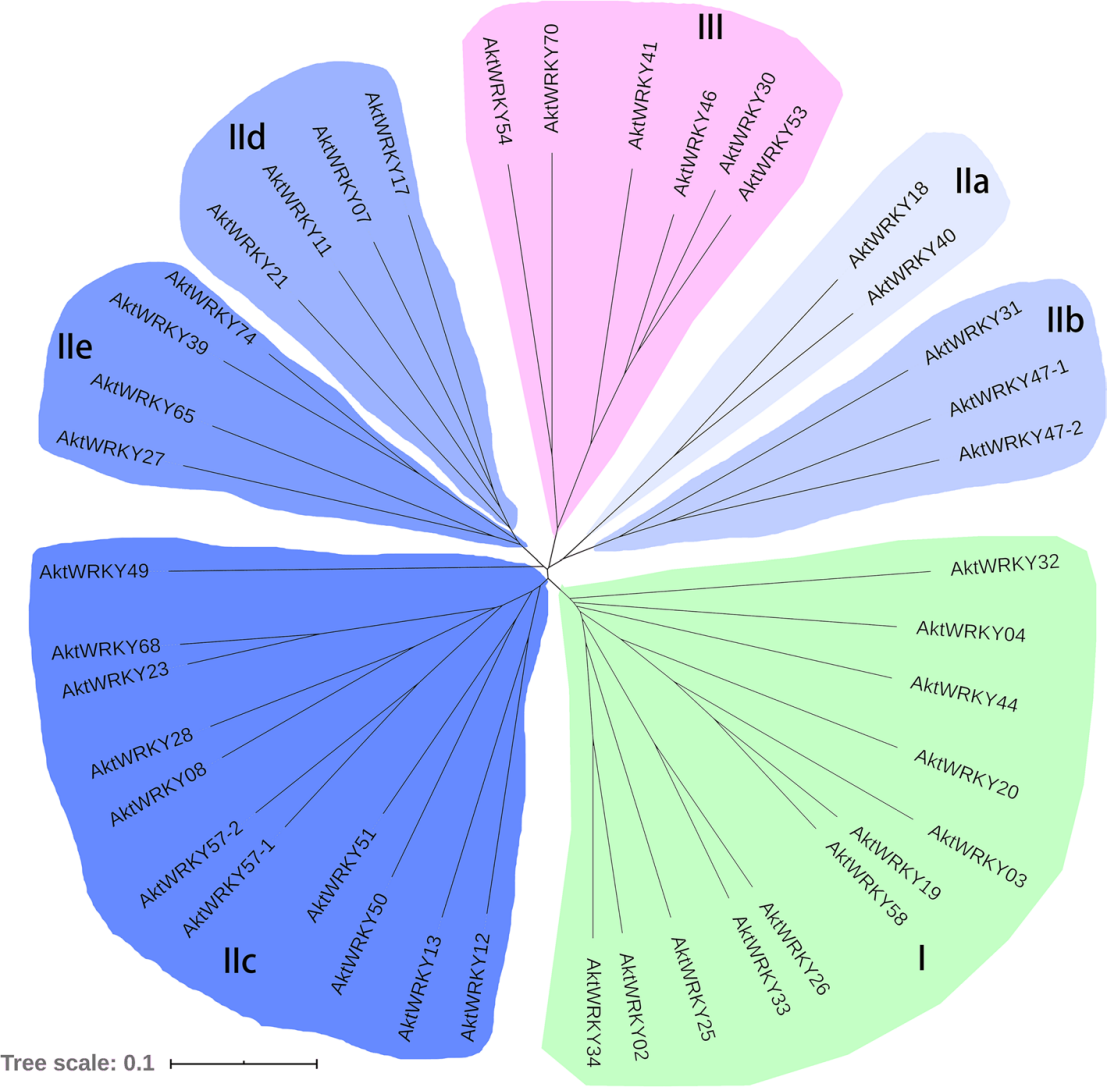
The phylogenetic tree of the AktWRKY proteins. The NJ tree was constructed from the amino acid sequences of AktWRKYs using MEGA10.0 with 1000 bootstrap replicates

As one of the largest gene families of transcriptional regulators in plant, WRKY transcription factor family plays critical roles in regulating plant growth and development as well as abiotic or biotic stress responses [38–40]. Although the functions and evolutionary relationships of WRKYs in several model plants have been investigated, little is known about this family genes in the Chinese traditional medicinal plant, *A. trifoliata*. To systematically explore the organizational structure, evolutionary relationship and function of WRKYs in *A. trifoliata*, the full-length transcriptome-sequencing data of this species were applied to identify WRKY genes *in silico*. A total of 42 *AktWRKY* genes were identified by searching the transcriptome-sequencing datasets using total Arabidopsis *WRKY* genes as queries (Table 1 and Additional file 1). All 42 deduced AktWRKY proteins contained at least one highly conserved WRKY domains, while 11 of them had two WRKY domains. As shown in Table 1, the deduced AktWRKY proteins contained amino acid residues between 145 (AktWRKY51) to 747 (AktWRKY34), the range of which was similar to that of other dicotyledons, such as *Glycyrrhiza glabra* and *Santalum album* [41, 42]. Their molecular weight (MW) varied between 16.77 kDa (AktWRKY51) to 81.41 kDa (AktWRKY25), while the isoelectric point (pI) of 21 AktWRKYs were acidic and the other 21 were basic proteins. A neighbor-joining phylogenetic tree was preformed to investigate the phylogenetic classification of the WRKY proteins in *A. trifoliata* according to previous report [5]. As shown in phylogenetic tree, all 42 deduced AktWRKY proteins were clustered into three main groups, namely, Group I, II, and III (Fig. 1). Seven AktWRKYs containing one WRKY domain and a C_2_CH zinc-binding motif at the C-terminal were classified as Group III, while twenty-three main clustering proteins with one WRKY domain and a C_2_H_2_ motif were clustered into Group II, which was further divided into five subgroups, named Group IIa-IIe. The twelve remaining proteins were included in Group I.

**Table 1:** Identified WRKY genes in *A. trifoliata*

Gene name	Protein length	Subcellular Location	pI	MW (kD)	Group	N-WRKY domain	C-WRKY domain
AktWRKY02	735	Nuclear	5.64	79.74	I	320–378	529–588
AktWRKY03	224	Nuclear	7.14	24.86	I	88–146	
AktWRKY04	520	Nuclear	8.17	56.64	I	235–293	412–471
AktWRKY07	310	Nuclear	9.66	34.22	II-d	237–297	
AktWRKY08	318	Nuclear	6.97	35.65	II-c	177–236	
AktWRKY11	220	Nuclear	9.6	24.91	II-d	147–207	
AktWRKY12	215	Nuclear	7.55	24.49	II-c	140–199	
AktWRKY13	227	Nuclear	9.34	26.06	II-c	150–209	
AktWRKY17	337	Nuclear	9.65	36.87	II-d	257–317	
AktWRKY18	310	Nuclear	8.85	34.21	II-a	154–214	
AktWRKY19	628	Nuclear	6.93	69.45	I	279–337	438–497
AktWRKY20	508	Nuclear	4.88	55.99	I	162–220	339–398
AktWRKY21	256	Nuclear	9.74	29.03	II-d	185–245	
AktWRKY23	309	Nuclear	5.61	34.66	II-c	150–209	
AktWRKY25	742	Nuclear	5.88	81.41	I	267–325	485–544
AktWRKY26	598	Nuclear	7.71	66.37	I	254–312	423–482
AktWRKY27	249	Nuclear	9.62	28.27	II-e	138–198	
AktWRKY28	309	Nuclear	7.74	34.64	II-c	176–235	
AktWRKY30	297	Nuclear	8.2	33.72	III	211–273	
AktWRKY31	588	Nuclear	5.82	64.4	II-b	327–387	
AktWRKY32	525	Nuclear	7.91	57.08	I	210–269	386–445
AktWRKY33	590	Nuclear	6.66	65.49	I	254–312	421–480
AktWRKY34	747	Nuclear	5.91	80.95	I	326–384	542–601
AktWRKY39	278	Nuclear	5.89	32	II-e	79–139	
AktWRKY40	292	Nuclear	8.81	32.8	II-a	136–196	
AktWRKY41	342	Nuclear	5.68	38.9	III	117–179	
AktWRKY44	474	Nuclear	9.4	52.46	I	181–239	393–452
AktWRKY46	181	Nuclear/Extracellular	8.34	20.68	III	117–179	
AktWRKY47–1	499	Nuclear	7.98	55.14	II-b	269–329	
AktWRKY47–2	492	Nuclear	7.13	54.66	II-b	255–315	
AktWRKY49	300	Nuclear	5.72	33.11	III	116–175	
AktWRKY50	198	Nuclear	6.09	22.84	II-c	115–174	
AktWRKY51	145	Nuclear/Cytoplasmic	5.7	16.77	II-c	83–142	
AktWRKY53	343	Nuclear	5.17	38.47	III	121–183	
AktWRKY54	319	Nuclear	5.6	36.58	III	128–190	
AktWRKY57–1	292	Nuclear	8.13	32.51	II-c	148–207	
AktWRKY57–2	303	Nuclear	7.7	34.01	II-c	156–215	
AktWRKY58	646	Nuclear	5.96	70.88	I	278–336	455–514
AktWRKY65	270	Nuclear	5.8	30.21	II-e	74–134	
AktWRKY68	306	Nuclear	6.03	34.4	II-c	147–206	
AktWRKY70	323	Nuclear	5.7	36.82	III	138–200	
AktWRKY74	249	Nuclear	5.6	28.33	II-e	55–115

**Fig. 2:**
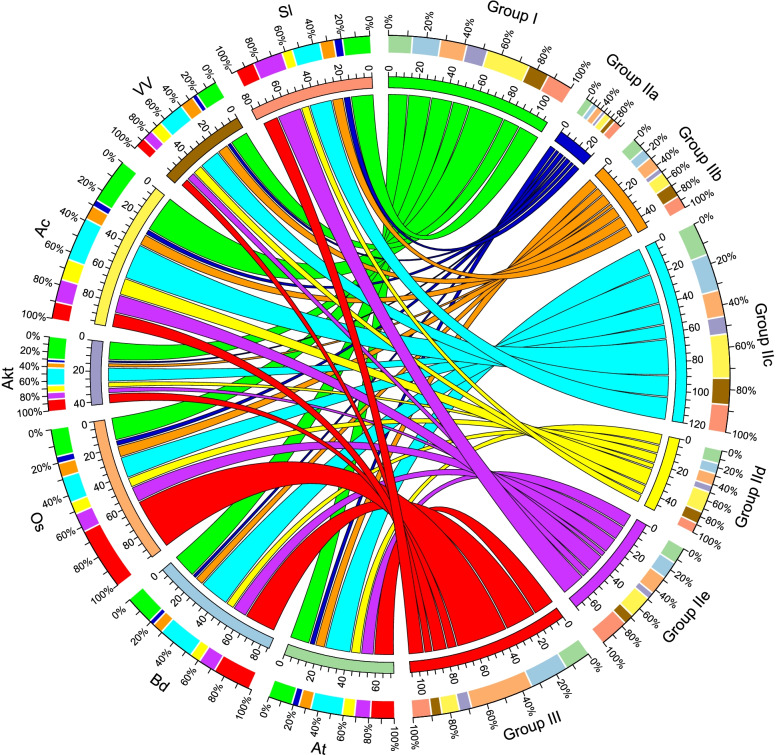
The distribution of WRKY transcription factors from Arabidopsis, *B. distachyon*, rice, *A. trifoliata*, kiwifruit (*A. chinensis*), grape and tomato. The width of the band represents the percentage of WRKYs in each group. Green represents group I, blue represents group IIa, orange represents group IIb, cyan represents group IIc, yellow represents group IId, purple represents group IIe, and red represents group III

The number of *AktWRKY* genes belonging to each subgroup was compared to the number of WRKYs in other plant species, in which the WRKY gene family has been fully identified and characterized, including Arabidopsis, *Brachypodium distachyon*, rice, kiwifruit (*Actinidia chinensis*), grape and tomato (Fig. 2 and Additional file 2) [21, 43, 44]. Comparing the number of WRKY genes in each subgroup, Group I and Group IIc experienced a significant expansion by having majority members in dicot species. In addition, the number of Group I WRKY genes was similar in *A. trifoliata*, Arabidopsis, *B. distachyon*, rice, grape and tomato, but it showed a significant expansion in kiwifruit, which might attribute to the two whole-genome duplications of kiwifruit genome [45]. On the contrary, it was apparent that variations in the number of WRKY genes in Group III were the primary cause of the diversity of WRKY gene family size in monocot species, i. e. *B. distachyon* and rice [21, 31]. These results suggested that numerous duplications and diversifications of Group III WRKY genes might be occurred after the divergence of the monocots and dicots. Previous studies have reported that the Group III of WRKY genes, as a newly defined group, was the most dynamic group with respect to gene family evolution [46]. In this study, the number of WRKY genes in Group III was relatively less in *A. trifoliata*, suggesting that this group had experienced less gene duplications during the evolutionary course, which could attribute to the fact that *A. trifoliata* located at the basal clade of the phylogenetic tree.

### Conserved motifs of AktWRKYs

**Fig. 3:**
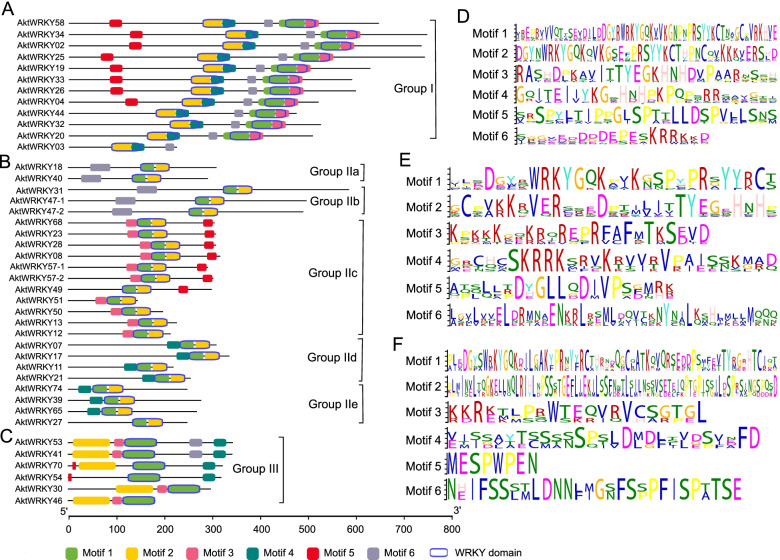
Diagram showed information of different motifs and their sequence logos for all AktWRKY proteins. **A**-**C** Distribution of conserved motifs in the 42 AktWRKYs. **A**, **B**, and **C** for Group I, Group II and Group III, respectively. Each motif was represented by a colored box. Blue hollow boxes represented the WRKY domains. **D**-**F** The logo of each motif (Color figure online). **D**, **E**, and **F** for Group I, Group II and Group III, respectively

To better understand the conservation and diversity of AktWRKYs, the conserved motifs of all the putative AktWRKY proteins were predicted by MEME online program. The distribution of motifs in each group and the diversity of conserved motifs were shown in Fig. 3. Generally, most members within the same group or subgroup shared a similar motif composition, which implied that AktWRKYs homologs located under the same group might have similar functions. However, AktWRKY members of different groups had no common conserved motifs except for the WRKY domain at the C-terminal. The motif 1 and 2 of group I contained conserved heptapeptides WRKYGQK sequence, representing the C-terminal and N-terminal WRKY domains, respectively. Obviously, the C-terminal and N-terminal WRKY domains were differentiated, suggesting that these two WRKY domains might be different in origin or functional differentiation. It was consistent with the fact that the specific binding to W-box was mediated mainly by the C-terminal WRKY domain, whereas N-terminal WRKY domain showed weak binding activity [9]. Further, insight into motif analysis of group II indicated that most of members of group IIa and IIb shared similar motif composition, while group IId and IIe shared motif 1, 2 and 4. The members of group IIc were relatively different with other subgroup members, even within subgroup itself. Most members of group III contained motif 1, which shared a tripeptide HTC residue at the C-terminal of the motif. Overall, these results present group-based motif analysis of AktWRKYs, while the functions of most of these motifs need to be further investigated.

### Comparative analysis of WRKY domains

**Fig. 4:**
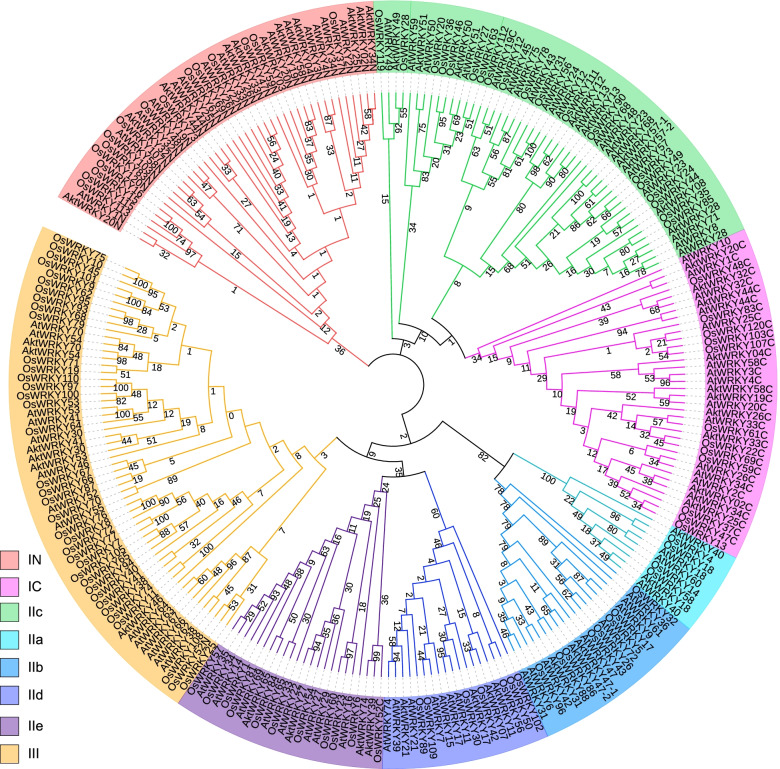
NJ analyses of 252 conserved WRKY domains from *O. sativa*, Arabidopsis, and *A. trifoliata*. The domains clustered into eight major subgroups, IN, IC, IIa, IIb, IIc, IId, IIe, and III

**Fig. 5:**
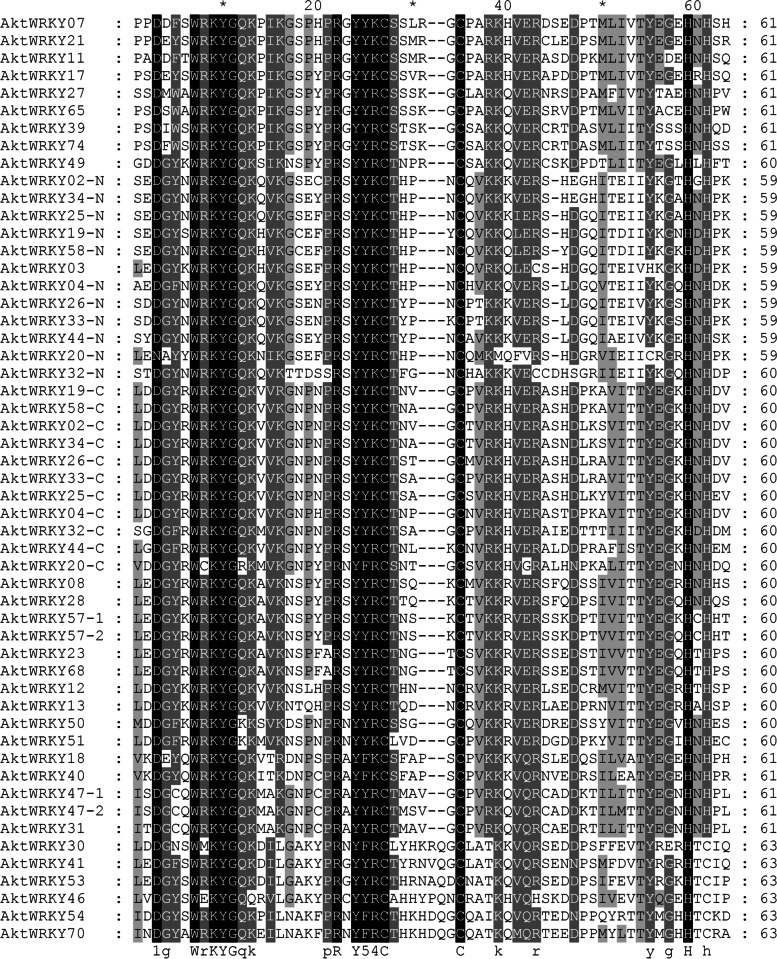
ClustalW amino acid sequence alignment of typical *A. trifoliata* WRKY domains. Gaps (dots) have been inserted for optimal alignment. Black and gray shading indicate the presence of identical and conserved amino acid residues, respectively. Consensus amino acid residues are shown below the alignment

To understand the diversity and evolutionary relationships of the AktWRKY domains, we compared the WRKY domains from the two other sequenced plant genomes (*O. sativa* and Arabidopsis). An unrooted neighbor-joining comparative phylogenetic tree was constructed by using MEGA software version 7 from the 252 conserved WRKY domains among these three plant species. As shown in Fig. 4, the complete WRKY domains were divided into three major groups (I, II and III), and all groups were present in monocots and eudicots. Of the three major groups, group II was the largest major group in the phylogenetic tree, with 118 WRKY domains distributed in five subgroups, including 9 in IIa, 19 in IIb, 49 in IIc, 18 in IId and 23 in IIe. Group I contained 77 WRKY domains, which were divided into subgroup IN and IC, containing 41 and 36 WRKY domains, respectively. Apparently, the subgroup IN and IC were clustered into different clades, suggesting that these domains originated from different ancestors and maintained their own differentiation after the lineage divergence. In addition, group I was considered to be the oldest group, located on the basal clade of the phylogenetic tree [47]. Subgroup IC and IIc were closely clustered, which was consistent with the previous results [48]. Meanwhile, no species-specific WRKY domain subgroups were observed in these three species, and WRKY domains belonging to the same group had similar conserved domain compositions, implying that WRKY family genes were conserved during plant evolution. Furthermore, multiple sequence alignment of the 42 AktWRKY protein domains was performed based on the conserved WRKY domain using clustal W software, containing approximately 60 amino acids for each AktWRKY (Fig. 5). The highly conserved heptapeptide sequence WRKYGQK was found within a total of 37 AktWRKYs, while five proteins (AktWRKY20, AktWRKY30, AktWRKY46, AktWRKY50 and AktWRKY51) were different due to one or two amino acid substitution. The protein AktWRKY50 and AktWRKY51 were found to contain a WRKYGKK sequence, while AktWRKY20, AktWRKY30 and AktWRKY46 contained a WCKYGRK, WMKYGQK and WEKYGQQ sequence, respectively. In addition, CX_4_CX_22-23_HXH and CX_4_CX_23_HXH zinc finger motifs were found in the N-terminal and C-terminal of group I AktWRKYs, respectively. CX_5_CX_23_HXH motifs were found in subgroup IIa, IIb, IId and IIe AktWRKYs, and CX_7_CX_23_HTC motifs were found in group III AktWRKYs, while the zinc finger motifs in subgroup IIc were same with C-terminal of group I AktWRKYs.

### Tissue-specific expression patterns of *AktWRKY* genes

**Fig. 6:**
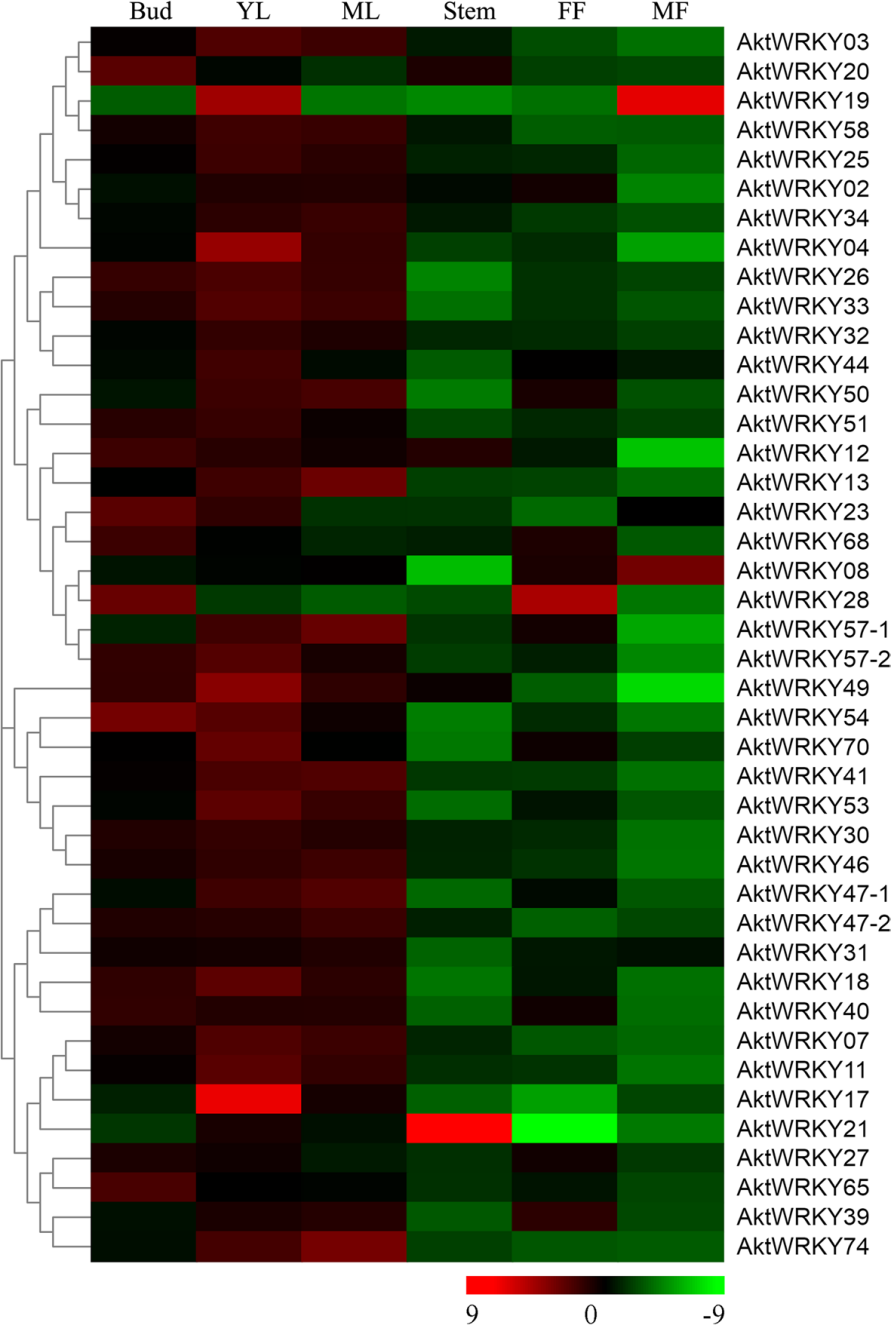
Expression patterns of *WRKY* genes in *A. trifoliata* in different tissues. YL for young leaves, ML for mature leaves, FF for female flowers, MF for male flowers. The expression values of the 42 *AktWRKY* genes were assessed upon the qPCR result analysis. Red represents a higher level of abundance while green signify lower expression levels

Numerous studies have demonstrated that the abundance of transcription factor genes varied greatly in different tissues and at different developmental stages, and played critical roles in regulating plant growth and development. For instance, *WRKY12* was specifically expressed in pith and cortex cells of stem and hypocotyls, playing a critical role in pith secondary wall formation, while *WRKY13* transcripts were highly abundant in the juvenile phase but decreased over time, implying that WRKY13 was involved in the control of age-mediated pathway [17–19]. Thus, tissue- and developmental stage-specific gene expression patterns might provide clues to gene functional divergence during evolution [49]. To investigate the patterns and expression levels of putative *AktWRKY* genes, the expression levels of 42 *A. trifoliata WRKY* genes in six tissues (buds, young leaves, mature leaves, stems, female flowers and male flowers) were determined by qRT-PCR (Fig. 6). Our results showed that *AktWRKY* genes could be detected in all test tissues, and exhibited distinct expression patterns. Generally, most *AktWRKY* genes were relatively highly abundant in leaves, especially in young leaves, while most *AktWRKY* genes were expressed at low levels in stem and flowers. However, tissue-specific expression of *WRKY* genes was also observed in *A. trifoliata*. For instance, *AktWRKY21* was particularly highly expressed in stems but less expressed in female flowers. *AktWRKY17* exhibited extremely high levels in young leaves, but low in the other tissues, implying this gene might be involved in leaf development. *AktWRKY28* showed high levels of expression in buds and female flowers, while *AktWRKY19* were highly expressed in young leaves and male flowers. Previous reports demonstrated that *WRKY* genes with high expression in plant tissues were often found to be able to regulate target genes involved in the relevant processes of plant growth and organs development [50, 51]. Thus, tissue-specific *AktWRKY* genes in this study might provide some useful clues for further investigation of their biological functions in the growth and organs development of *A. trifoliata*. In addition, some clustered gene pairs showed the same expression pattern, such as *AktWRKY03*/*20*, *AktWRKY26*/*33*, *AktWRKY07*/*11*, *AktWRKY27*/*65*, etc., implying that they might be functionally redundant. On the other hand, *AktWRKY* gene pairs with different expression patterns might executed different biological functions in plant growth and development.

### Expression analysis of AktWRKY genes in response to phytopathogen

**Fig. 7:**
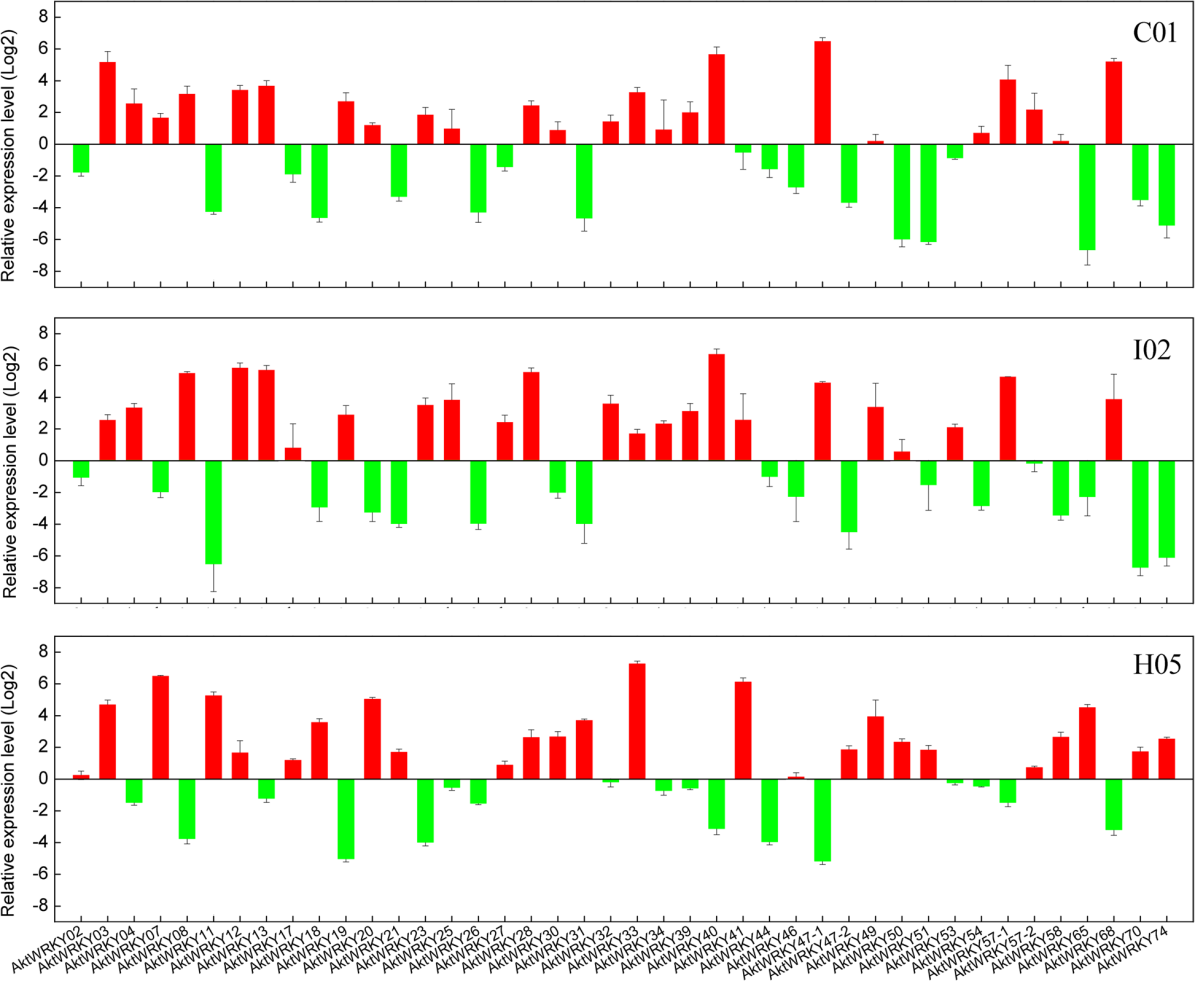
The expression level of 42 *AktWRKY* genes in three different varieties of *A. trifoliata* (C01, I02 and H05) after *C. acutatum* infection for 6 h. The red columns represented up-regulated genes, while the green columns represented down-regulated genes

As crucial components of plant defense signaling networks, WRKY genes were known to exhibit complex response patterns [16, 52]. The function of *WRKY* genes in the regulation of plant response to biotic stresses has been studied in many plant species, especially in Arabidopsis, rice and tomato [7]. It is well known that the functions of *AtWRKY* genes response to phytopathogens have been well studied. Expression profiles of *AtWRKY* genes under various phytopathogen stresses indicated that most of AtWRKYs were involved in various biotic stresses response (Additional file 5) [53–60]. For example, *AtWRKY3*, *AtWRKY4*, *AtWRKY53* and *AtWRKY70* played positive roles in disease defense against the necrotrophic fungal pathogen and the biotrophic pathogen *Pseudomonas syringae* [61–63]. AtWRKY33, which was activated by two sigma factor binding proteins, SIB1 and SIB2, was essential for defense toward the necrotrophic fungus *Botrytis cinerea* [62, 64, 65], while AtWRKY18 and PtrWRKY18 could activate pathogenesis-related genes, and increase resistance to the biotrophic pathogens [66, 67]. Moreover, some *WRKY* genes, such as AtWRKY7 and AtWRKY48, had direct negative effects on plant defense responses [68, 69]. In regards to *AktWRKY* expression in response to biotic stresses, three different varieties of *A. trifoliata* (C01, susceptible variety I02 and resistant variety H05) were infected with a polyphagous fungal plant pathogen, *C. acutatum*. Then, qRT-PCR was performed on *C. acutatum* infected leaves in order to investigate the expression profiles of *A. trifoliata* in response to *C. acutatum* (Additional file 3). As shown in Fig. 7, a large number of *AktWRKY* genes were induced by *C. acutatum* infection. It has been shown that four *WRKY* genes (*AktWRKY03*, *12*, *28* and *33*) were highly expressed in all three tested *A. trifoliata* varieties, which were consistent with the previous reports that their homologs (AtWRKY3, BrWRKY12, BnWRKY15 and AtWRKY33) enhanced resistance to phytopathogens through transcriptional activation of defense-related genes [61, 65, 70, 71]. These results suggested that, at least, these four genes might be involved in plant disease resistance. Furthermore, *AktWRKY28* showed an expression pattern different from *AktWRKY03* and *AktWRKY33*, with high abundance 6 hpi in susceptible variety, but low abundance in the resistant one, although the expression level of which were almost similar in the control samples (Additional file 6). Such divergent behaviors were previously found in rice and cacao [72, 73]. For instance, knock-out of *OsWRKY28* led to a two-fold increase in resistance to *Magnaporthe oryzae*, while overexpression of *OsWRKY28* resulted in enhanced susceptiblility to *M. oryzae* [72, 74]. Therefore, it was speculated that AktWRKY28, like OsWRKY28 and TcWRKY28, might act as negative regulator of basal defense responses to pathogens infection. Generally, the expression patterns of *AktWRKY* genes in C01 and susceptible variety I02 were similar, but distinctly different in resistant variety H05, implying a functional divergence of AktWRKYs in response to phytopathogen infection between susceptible and resistant varieties. In total, a number of nine *AktWRKY* genes (*AktWRKY11*, *18*, *21*, *31*, *47–2*, *51*, *65*, *70*, and *74*) increased after *C. acutatum* infection in H05, but decreased in C01 and I02. On the other hand, thirteen *AktWRKY* genes, including *AktWRKY04*, *13*, *14*, *19*, *23*, *25*, *32*, *34*, *39*, *40*, *47–1*, *57–1*, and *68*, were up-regulated after *C. acutatum* infection in C01 and I02, but down-regulated in H05. For example, AktWRKY18 and AktWRKY70 were induced by *C. acutatum* in resistant variety (Additional file 6), which was consistent with the previous results that WRKY18 and WRKY70 could positively modulate defense-related gene expression and disease resistance in Arabidopsis [63, 67]. Although analysis of WRKY expression was helpful in discriminating the role and function of these proteins at the tissue and organism levels, further molecular and biological experiments are needed to investigate their biological function.

### Gene ontology annotation and interaction analysis of AktWRKY proteins

**Fig. 8:**
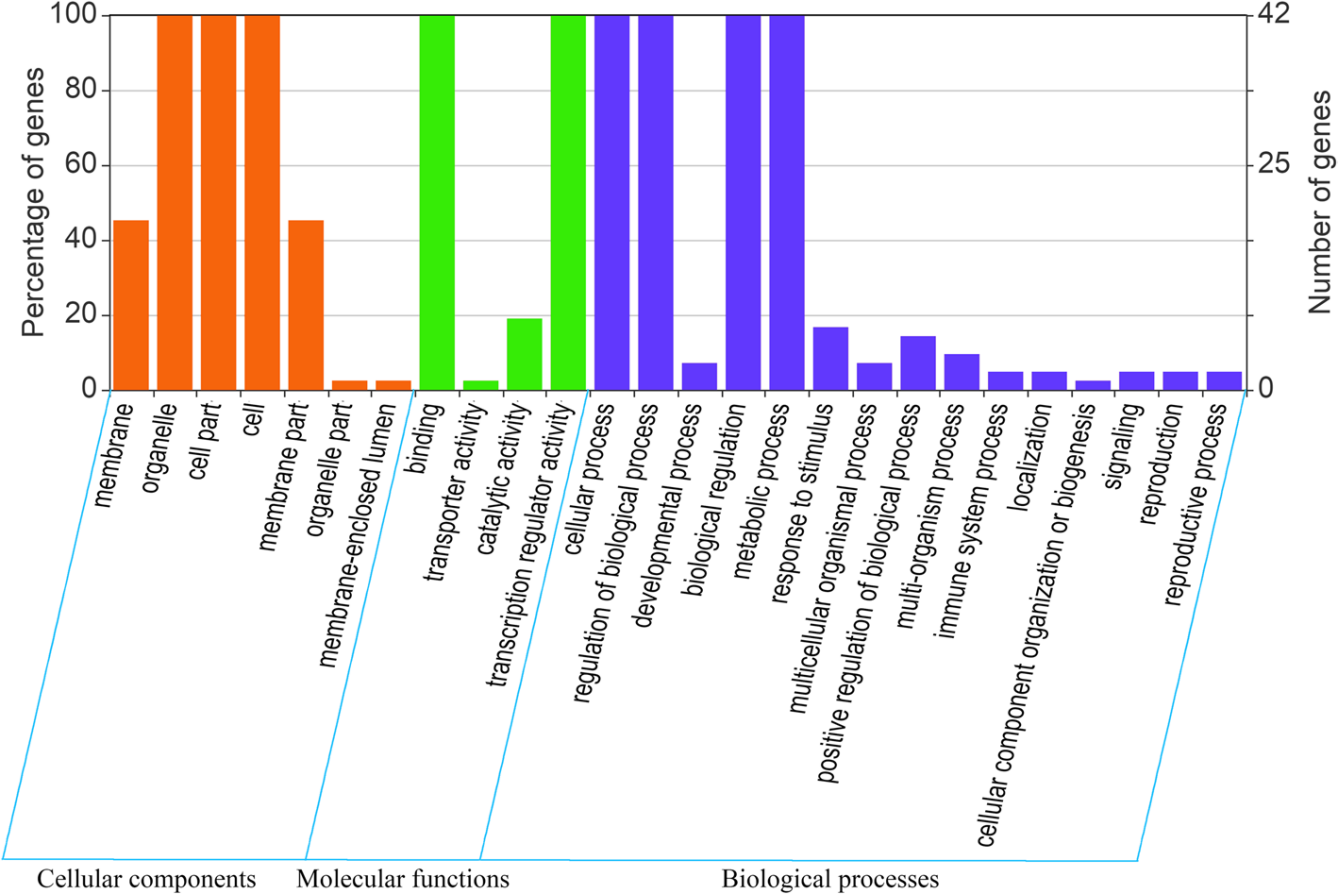
Gene ontology analysis of identified AktWRKYs. Three main categories, including cellular component, molecular function, and biological process were defined by GO classification. Left and right y-axis represented the percentage and number of genes, respectively

Functional annotation of proteins and prediction of protein-protein interaction could help us predict their possible regulatory functions, and provide great support for further investigation of gene families. Based on the similarity of peptide sequences, the Gene Ontology (GO) annotations of one protein could be coordinately transferred to another [75, 76]. Here, GO annotations of 42 AktWRKY proteins were analyzed using the Blast2GO basic tool (Fig. 8). Among the protein sequences annotated in the GO database, AktWRKY proteins were categorized into three main categories (i. e. biological processes, molecular functions, and cellular components) and 26 subcategories. In the category of biological process, most AktWRKYs were identified to be involved in the ‘regulation of cellular process’ (GO:0009987), ‘regulation of biological process’ (GO:0050789), biological regulation (GO:0065007), metabolic process (GO:0008152), and so on. However, three AktWRKYs (AktWRKY08, 27, and 28) were predicted to be involved in developmental process, including cell differentiation and leaf senescence, while four AktWRKYs (AktWRKY26, 33, 50, and 51) appeared to be involved in defense response to biotic stimulus. The molecular functions of AktWRKYs were associated mostly with ‘binding’ (GO:0005488) and ‘transcription regulator activity’ (GO:0140110). The cellular component of this protein family included organelle (GO:0043226), cell part (GO:0044464) and cell (GO:0005623), besides, all AktWRKYs were predicted to be localized in the nucleus (Table 1).

**Fig. 9:**
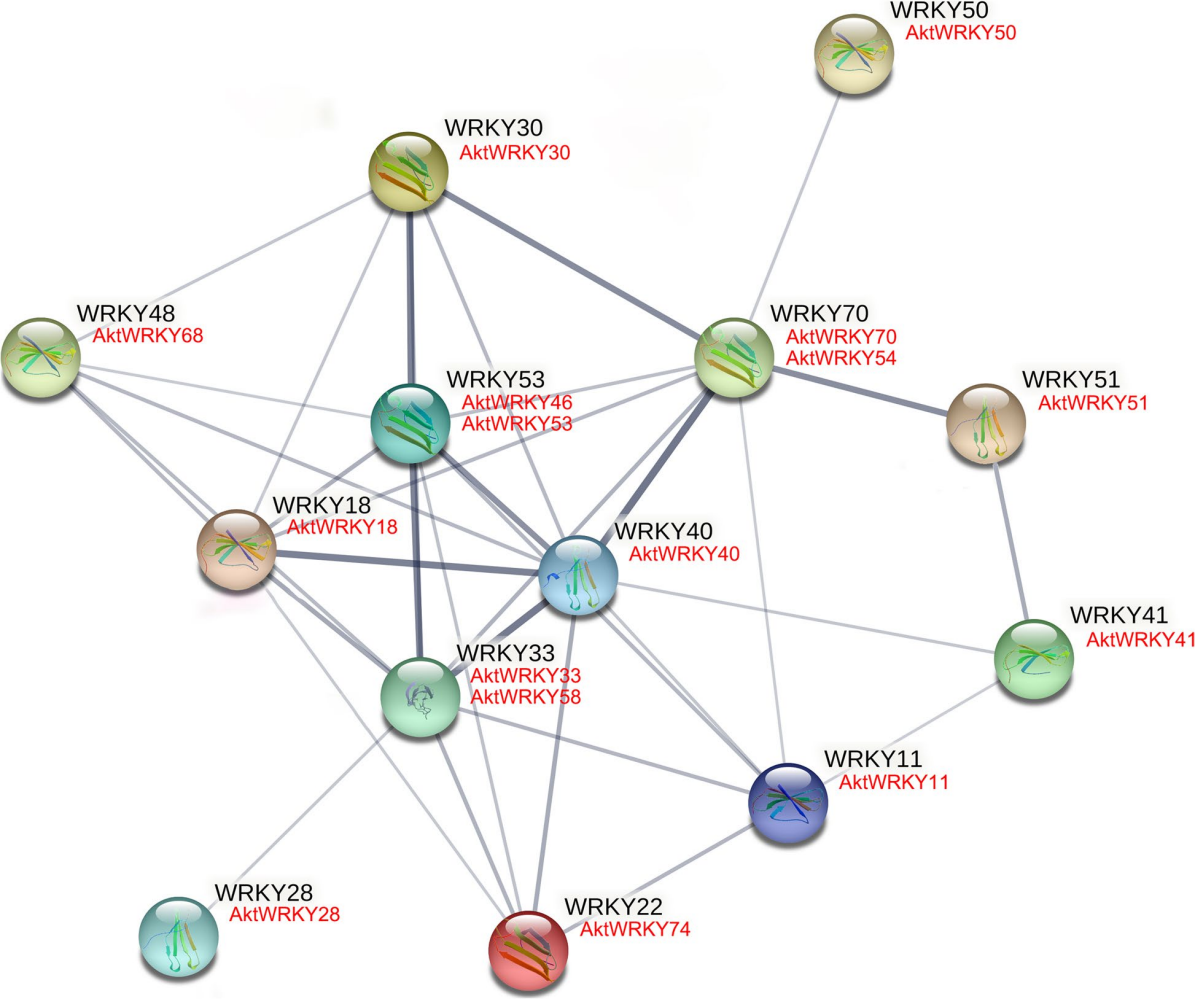
Protein-Protein interaction of AktWRKYs based on AtWRKYs orthologs as predicted by STRING search tool. The thickness of the lines represents the level of interaction between proteins

Subsequently, to systematically analyze the interaction of AktWRKY proteins, a predicted protein interaction network containing 16 AktWRKY proteins was constructed based on Arabidopsis homologous genes using STRING 11.0 software with the confidence parameter set at a threshold of 0.35 (Fig. 9). Among these proteins, the interaction between AtWRKY33 (AktWRKY33 and 58) and AtWRKY22 (AktWRKY74) were related to the Kyoto Encyclopedia of Genes and Genomes (KEGG) signaling pathway of plant MAPK signaling pathway (ath04016) and plant-pathogen interaction (ath04626) [77–81]. The interaction network among AtWRKY18 (AktWRKY18), AtWRKY33 (AktWRKY33 and 58), AtWRKY40 (AktWRKY40), AtWRKY53 (AktWRKY46 and 53), and AtWRKY70 (AktWRKY54 and 70) enriched significantly GO term in defense response, including bacterium (GO: 0042742), fungus (GO:0050832), chitin (GO:0010200) and salicylic acid (GO:0009751) [22, 65, 67, 82]. In addition, these five AtWRKYs and corresponding ortholog AktWRKYs were also involved in a stronger interaction network with other proteins. Overall, the results showed that there were multiple interactions among AktWRKY proteins, implying that AktWRKY proteins were involved in multiple stress responses.

## Conclusion

In conclusion, a total of 42 *WRKY* genes were identified based on transcriptome sequences, which was the first study on the organizational structure and abundance of WRKY in *A. trifoliata*. The classification, protein structure, conserved motif composition, and phylogenetic relationship of AktWRKYs were systematically analyzed and compared, which provide a basis for further study of the molecular and functional structure of AktWRKYs. Furthermore, based on qPCR results, the expression patterns of tissues-specific and phytopathogen-responsive *AktWRKYs* were obtained, providing useful information for further investigation of the function of AktWRKYs in response to biotic stresses. Our study could help researchers better understand the function and regulatory mechanism of WRKYs in *A. trifoliata* during pathogen response.

## Supplementary Information

**Additional file 1. **The CDs and amino acid sequences of WRKYs in *A. trifoliata.*

**Additional file 2.** The number of WRKY genes belonging to each subgroup in different species.

**Additional file 3. **Expression data of the *AktWRKY* family genes after *C. acutatum* infection.

**Additional file 4. **The list of primer-sets of *AktWRKY* genes for qRT-PCR.

**Additional file 5. **Expression patterns of *AtWRKY* genes in response to biotic stresses obtained from the GEO (A) and the Arabidopsis eFP Browser (B). (A) Expression profiles of *AtWRKY* genes in leaves (12, 24, 48 and 72 hpi) under *Vibrio vulnificus* (GSE61418); mature leaves (24 hpi) under *Sclerotinia sclerotiorum* (GSE106811); rosette leaves (3dpi) under *Alternaria brassicicola* (GSE83478); leaves (24 hpi) under Pst DC3000 hrcC (GSE107786); leaves (48 hpi) under *Golovinomyces orontii* (GSE129011); seedlings (0.5 hpi) under flg22 (GSE146189); roots (1, 2, 3, 4 and 6 dpi) under *Fusarium oxysporum* (GSE168015); and seedlings (24 hpi) under *Agrobacterium* (GSE179628). (B) Expression profiles of *AtWRKY* genes in leaves under phytopathogens. Bc, *Botrytis cinerea*; Ps, *Pseudomonas syringae*; Pi, *Phytophthora infestans*; Eo, *Erysiphe orontii*. The red represented up-regulated genes, while the blue represented down-regulated genes. The gray block indicated missing data.

**Additional file 6. **The expression level of *AktWRKY* genes provided as -Δ*Ct* values in three different varieties of *A. trifoliata* after *C. acutatum* infection.

## Data Availability

Data generated or analyzed during this study were are available in the NCBI Short Read Archive with accession number PRJNA795256 and the Additional files. Public Arabidopsis expression datasets were obtained from the AtGenExpress Consortium (Arabidopsis eFP Browser) and the Gene Expression Omnibus (Accession No. GSE61418, GSE106811, GSE83478, GSE107786, GSE129011, GSE146189, GSE168015 and GSE179628).
