# Supplementary material for: Characterization of the WRKY gene family in Akebia trifoliata and their response to Colletotrichum acutatum
Source: BMC Plant Biol. 2022 Mar 14;22:115. doi: 10.1186/s12870-022-03511-1 (PMC8919620; doi:10.1186/s12870-022-03511-1)
Supplement: Supplementary file 1 — Additional file 1. The CDs and amino acid sequences of WRKYs in A. trifoliata. [file 12870_2022_3511_MOESM1_ESM.docx]

Additional file 1 The CDs and amino acid sequences of WRKYs in *A. trifoliata*

| Gene name | Sequence |
| --- | --- |
| AktWRKY02 | ATGGTTGGGAGTGATGAACACATTGCTGTAATTGGGGATTGGGTGCCGCAAAATTCCAGCAATGGGACCTTTTTCTCTTCAATCTTAGGAGATGATTTCGGTTCAAGATCGTTCTCCGAGATTTTAGGAGACAATGGGAATGAAGGGCCTTTCATGGGATCTACTGAAAAGCTAAAATTGGCAATGGATCTTGAAGAAGAAGATGGGGCAGGAGTGGGTGTTTCTGATGATCATTTGTCTGACTCAAGCAATTTTTCCAGCCAGAAATCAAGCTATCGTGGGGGTCTTGCTGAGAGGATGGCGGCAAGAGCTGGGTTTAATGCTCCAAAGTTGAACACAGCGAGGATTAGATCTGCTAATCTGTCCTCCACTGAAGTTCGGTCTCCTTACCTTACGATTCCACCAGGTTTAAGTCCGACGACATTGCTGGATTCCCCGGTTTTCCTTTCCAATTCTCTGGTTCAGCCATCTCCAACAACAGGAAAATTTTCCTTTGGCCCAAGCACTAGCACTAAAACCTCCATACCAATCCCCGAAGCTCCTGACAGAGAGAAAGATGATTTATTTGAAGATGTTGATACCTCGTCATTTGTATTCAAGCCTCACATGGAATCGAGTTCCTCATTTTTTCACAGTGCGGCAAATAAAGAAACTCCTTCCACAAATCCTCCACTTTCCTTTCCCATTATTGAGGTGTCGGTCCAGTCAGAGCACACTCTTCAGTCACGAAGTCTCGAAGCCAGTGAAGGTCTCTCCCAGAATAAAAACAATTTCCATCCATGGGCAGACTTCGCCAAATCATCTGATGAAAGAGATATTAACGGTAATAATGTCAATTTGGAACCAAGGGTTTCTGATCCTGTAATTGCCAATACCCGGAATTCTCCTGAAGTTGATGATCAGCAGGACGGGGAAGGCTGTGGGGAGCTGAATTCCACCGTTGTTGGTGGTGCCACATCTGAGGATGGGTACAATTGGAGAAAGTATGGGCAGAAACAGGTAAAAGGAAGTGAGTGTCCTCGAAGTTATTACAAATGCACACATCCAAATTGTCAGGTTAAAAAGAAGGTGGAGCGATCTCATGAGGGCCACATTACAGAGATCATCTACAAGGGGACTCACGGTCACCCTAAACCTCCACCTAACCGTCGATCGGGTATTGGGTTTTCTAATCCACAAAATGATATGCAGCTTGACATTCCTGAGCATGCTGGGGCACAGGCTGTGGCCGAGGCTGACAACATGATTGGACACGTTTCACGAGGTCCTGATTGGAGGCATGACAACCTGGAGGCCACATCATCTGAATCTATGGCCCCCGAATTCTGCGACCCATCTACCTCTATGCAGGCACAAAAAGGCCCTCACTTTGAACCCATTACTGTAGATGCCTCACCGTCCATGTCTAATGATGAAGATGAGGATGATAGGGGGACACATGGAAGTGCATCATTGGGATATGATGGCGAAGGATATGAGTCTGAGTCAAAGAGAAGAAAGATAGATGCGATGGAAATAGGCGGGGCCGCAAGAGCCATTCGTGAACCAAGAGTTGTCGTCCAAACAGCCAGTGAGGTTGACATCCTTGATGATGGATACCGCTGGCGCAAGTATGGGCAGAAAGTCGTCAAAGGAAATCCAAATCCAAGGAGCTACTACAAGTGCACTAATGTAGGTTGCACCGTTAGAAAGCACGTAGAACGGGCATCACACGACCTCAAGTCAGTTATCACCACGTATGAGGGGAAGCATAATCATGATGTTCCCGCTGCAAGGAATAGCAGCCATTCCAGCTCTGGGACCTCTAACACAGTGCCCAATGCAACCTCAGCTGCTCAATCCTTCGTCCACAGGCCTGAGCCATCTCAAATTCACGATAACATGGCAAGATTTGATGGGCGTGCATCATTTGGTGCATTCGGTCTGCCTGGACGACAACATATGGGGCCCTCACCTGCCTTTTCATTTGGAATGAATCAGCAAAGTCTGGCCAACCTGGCGATGGCTGGGGTAGGCCCTGGCCAAGGTAAGCGGCCTTTCCATCCGTATTTAGGACCGCAACATCACTTGAATGATGAAGGTGGTGGGTTCATGATGCTGAAAGCAGAACCAAAGAATGAACCTGTGTCTGAGCCTTGTCTGAACTTATCGAATGGGCCGACAGTTTATCATCATCAGATCATGGGTAGACAGTCTCTTGGCCCTTAG |
|  | MVGSDEHIAVIGDWVPQNSSNGTFFSSILGDDFGSRSFSEILGDNGNEGPFMGSTEKLKLAMDLEEEDGAGVGVSDDHLSDSSNFSSQKSSYRGGLAERMAARAGFNAPKLNTARIRSANLSSTEVRSPYLTIPPGLSPTTLLDSPVFLSNSLVQPSPTTGKFSFGPSTSTKTSIPIPEAPDREKDDLFEDVDTSSFVFKPHMESSSSFFHSAANKETPSTNPPLSFPIIEVSVQSEHTLQSRSLEASEGLSQNKNNFHPWADFAKSSDERDINGNNVNLEPRVSDPVIANTRNSPEVDDQQDGEGCGELNSTVVGGATSEDGYNWRKYGQKQVKGSECPRSYYKCTHPNCQVKKKVERSHEGHITEIIYKGTHGHPKPPPNRRSGIGFSNPQNDMQLDIPEHAGAQAVAEADNMIGHVSRGPDWRHDNLEATSSESMAPEFCDPSTSMQAQKGPHFEPITVDASPSMSNDEDEDDRGTHGSASLGYDGEGYESESKRRKIDAMEIGGAARAIREPRVVVQTASEVDILDDGYRWRKYGQKVVKGNPNPRSYYKCTNVGCTVRKHVERASHDLKSVITTYEGKHNHDVPAARNSSHSSSGTSNTVPNATSAAQSFVHRPEPSQIHDNMARFDGRASFGAFGLPGRQHMGPSPAFSFGMNQQSLANLAMAGVGPGQGKRPFHPYLGPQHHLNDEGGGFMMLKAEPKNEPVSEPCLNLSNGPTVYHHQIMGRQSLGP |
| AktWRKY03 | ATGGAGAAAGGAGGGAGTGGACAGGTGGACCCTTCCCCAACTATTGGTACCTTCACCAAGCCTCAAATCATGCATGATACTATTGGTTCTGAAAATATCTCTCTTCCAAACCCCTCAGTTCGCATGGTTACTTCGGTAGCCAGCGTACCAGCTGAAGTTGTTTCTGACAAGTTACATAAGAGAAAATTGTCAGACATTGCGGTCCAAGAAACACCATCTGATCAGAAAGGAACTATTACTTTGATAGCATCTGAGAGACCATTGGAAGATGGATATAAGTGGCGAAAATATGGGCAGAAACATGTTAAAGGAAGTGAATTTCCACGGAGCTATTACAAGTGCACACATCCCAATTGTCAAGTCAGAAAGCAATTAGAATGCTCTCATGATGGGCAGATCACAGAGATTGTCCACAAGGGAAAGCATGATCACCCTAAGCCTCAGCCTAGTCGTCGAATGCCAATTGATACAATTTTCTCTATTCAAGAAGGACGATCAGATAGGTTTTCTTCTTTAAAAGGCACAAAATGCAGAGAGGCATCTCATGATATTGAGCCAAATGGTACCCCTAAGCTTTCTCCTGTCACAGCAAATGATGGTGACATAGGCAATGAAGCTGTGGACAATGATGATCCGGAGTCTAAGCACAGGTGCCTTAATACAGATACTTTTTGA |
|  | MEKGGSGQVDPSPTIGTFTKPQIMHDTIGSENISLPNPSVRMVTSVASVPAEVVSDKLHKRKLSDIAVQETPSDQKGTITLIASERPLEDGYKWRKYGQKHVKGSEFPRSYYKCTHPNCQVRKQLECSHDGQITEIVHKGKHDHPKPQPSRRMPIDTIFSIQEGRSDRFSSLKGTKCREASHDIEPNGTPKLSPVTANDGDIGNEAVDNDDPESKHRCLNTDTF |
| AktWRKY04 | ATGGCTGAAAAAGAAGGAGCTTCAAGAGCAACAGCTCCTTCCCGCCCTACAATTTCTCTCCCTCCTCGCTCTTCGGTTGAGAATCTTTTCATAGGTGGAGCTGGCGCGAGCCCTGGTCCGATGACACTCGTTTCAAATTTCTTCTCAGAGAACGATCCTGACTCGGAGTATCGATCCTTCTCTCAACTCCTCGCAGGGGCCATGGCTTCCCCTGTTGCGGCCACTGGTAGGAGGCCGAGCTTGTCGATGGAAAGCTCGATAGAGGATGATTCTTCCAAGGGGAAGGATTCGAGTGATGGAATTGAAAAAGATTTTCGGTTTAAGCAGAATCGACCCTTGAGTTTGGTGATTGCGCAGTCGCCCATGTTTACTGTTCCACCTGGGTTGAGCCCTGCTAGCTTGCTTGATTCACCAGGGTTCTTTTCGCCTAGTCAGGGACCCTTTGGAATCTCCCACCAACAGGCTTTGGCGCAAGTTACAGCTCAGGCTGCCCAATCACAACCTCATCTGCAAATCCCATCTGAATATTCGTCTTCTCTGTTAGCACCTCCTACAACTTCATTGGAACACCATCCATTCTATGCGACTCCCCCTCAGCAGATGCCACCCTTAATGTCAGCCCCTAAAAGTACCATGGTGGAATCATCTGCGGTTTCTTATTCTAGTCAGAGATCCCAACCTCCCCTTATTGTCGATAAGCCTGCCGAGGATGGCTTCAACTGGCGGAAATATGGGCAAAAGCAGGTGAAGGGCAGCGAATACCCTCGAAGCTACTATAAATGTACCCATCCAAATTGTCATGTCAAGAAACAGGTTGAACGTTCTCTTGATGGTCAAGTGACTGAGATTATCTATAAGGGTCAGCACAATCATGATAAACCTCAACCCAATAGACGTGCGAAAGAAAGTGGGAATTCAAATGGTAACTTAAATTTACACGGGAATCCTGGAATGGGTCACAGTGGGAATTTGAGCAGATCAAATGAAATTGTTGTTGCTCCTCCAAGGTCTAAGAGGGATCAAGAATCTAGCCAGGCAACACCTGAACGGTCATCTGGCTCAAGTGATGGTGAGGAAGTAGGTGATGCTGAAACAAGGGTAGATGAAGGGAATGGTGATGAACCTGATCCGAAGAGAAGGAATACAGAGGTCAGGGTAACTGAGCCAGCTTCTTCACACAGGGCAGTTTCAGAATCTAAGATCATTGTGCAGACAACAAGTGAAGTTGATCTTTTGGATGATGGTTATAGGTGGCGCAAGTACGGGCAGAAAGTCGTCAAAGGGAATCCTCATCCAAGGAGCTACTATAAATGCACAAATCCAGGATGTAATGTCCGTAAACATGTTGAGAGGGCTTCAACAGATCCAAAAGCAGTCATTACAACATATGAGGGAAAACACAATCATGATGTGCCAGCAGCAAAGACCAGCAGCCACAACACAGCCAACAATTATGTGTCCCAGCTAAAACCACAAGATGCATTGGTTAATAAGACAGATTCTGGACGCAGTGATCAACAGCCTGTAGCGCTTTTGAGATTAAAAGATGAGCAAATCACCTAA |
|  | MAEKEGASRATAPSRPTISLPPRSSVENLFIGGAGASPGPMTLVSNFFSENDPDSEYRSFSQLLAGAMASPVAATGRRPSLSMESSIEDDSSKGKDSSDGIEKDFRFKQNRPLSLVIAQSPMFTVPPGLSPASLLDSPGFFSPSQGPFGISHQQALAQVTAQAAQSQPHLQIPSEYSSSLLAPPTTSLEHHPFYATPPQQMPPLMSAPKSTMVESSAVSYSSQRSQPPLIVDKPAEDGFNWRKYGQKQVKGSEYPRSYYKCTHPNCHVKKQVERSLDGQVTEIIYKGQHNHDKPQPNRRAKESGNSNGNLNLHGNPGMGHSGNLSRSNEIVVAPPRSKRDQESSQATPERSSGSSDGEEVGDAETRVDEGNGDEPDPKRRNTEVRVTEPASSHRAVSESKIIVQTTSEVDLLDDGYRWRKYGQKVVKGNPHPRSYYKCTNPGCNVRKHVERASTDPKAVITTYEGKHNHDVPAAKTSSHNTANNYVSQLKPQDALVNKTDSGRSDQQPVALLRLKDEQIT |
| AktWRKY07 | ATGGCGGTGGAACTCATTAGAGATTACAGAAGCGAGAATCTAAAATCTAAAATGGAAGAAAATGCAGTCGAAGAAGCCGCTTCTGCAGGACTTGCAAGCGTGGAAAAGCTTCTAAGATTACTCTCTCATCAAAAACAACAACAACAATCATCCATGGAGGTTGAGATGGATTACAGATCTGTAGCAGATGTTGCTGTAACTAAGTTCAAGAAGGTTATTTCTTTACTGGGTCGGAATCGAACGGGTCATGCCCGATTCAGAAGAGGTCCATCTCCTTTGGTTTCTTCTCCATCTCAACAACATACACAACAAGTTGTTGATCCAACGGTTAAGATTTACTGTCCAACACCAATCCAACGACTCCCACCACCACTACCTCAAAGAAAGGAATCTTGTTCTTCAACTACGATTAATTTCACGACTTCCCCTCCAAATTCTTTCATGTCTTCGTTAACTGGAGATACAGATACAAAACAACAGATTTCGAATTCTTCGGCTTTTCAAATTACGAATCTTTCTCAGGTTTCTTCTGTTGGGAGGCCTCCTCTCTCTTCTTCTTCGTTGAAGAGAAAATGTAGCTTTTCTGATGATATGGGTGGTAATAAGTGCGGTGGAGGTTCTGGAAAGTGTCATTGTTCAAAGCGAAGAAAATCGAGGGTGAAGAGAGTTGTTAGGGTTCCTGCGATAAGTTCGAAGATGGCTGATGTTCCACCAGATGATTTTTCGTGGAGGAAATATGGGCAGAAACCCATTAAAGGATCTCCACATCCAAGGGGGTACTACAAGTGCAGTAGTCTTCGAGGTTGCCCTGCACGTAAACACGTGGAAAGAGACTCAGAAGATCCAACGATGCTGATAGTCACGTACGAAGGCGAGCACAATCACTCTCACTCGGTCGCAGAGACAAGTGGGTTAATTCTTGAATCGTCTTAA |
|  | MAVELIRDYRSENLKSKMEENAVEEAASAGLASVEKLLRLLSHQKQQQQSSMEVEMDYRSVADVAVTKFKKVISLLGRNRTGHARFRRGPSPLVSSPSQQHTQQVVDPTVKIYCPTPIQRLPPPLPQRKESCSSTTINFTTSPPNSFMSSLTGDTDTKQQISNSSAFQITNLSQVSSVGRPPLSSSSLKRKCSFSDDMGGNKCGGGSGKCHCSKRRKSRVKRVVRVPAISSKMADVPPDDFSWRKYGQKPIKGSPHPRGYYKCSSLRGCPARKHVERDSEDPTMLIVTYEGEHNHSHSVAETSGLILESS |
| AktWRKY08 | ATGTCTGATAAAACCCTAGATCACCACCATCACCATGACCCATACCATAACAATGATCAAACCAACATAAGTGGTACTAGATTTCCATTATCTAGTGATCCATCATCTTCTTTTGATCAGATGGCTACAAACATCCCATACTCATCATCTAACTATCTACATGGGTTTGATCCAGTGCACATGAGCTTCACTGAATCACTGTATGGTTCCATGGATTATAACACACTAAAAATGGCCTTCGACATGTCGTGCTCACCGTCTGAAGTTCTCTCTTCTGTTGATGGTGGTGGTGCTGGTCAGAAAACTGGTGGTCTTGGAGAATCAATATTGGAGGGTGGTGACATTCCGATGACACCGAACTCTTCGGTGTCAAATTCATCTTCTAATGAGAAAGACGCCGCTGAAGAAGAGACGACGAAGAGTACTAAGAAAGATGAGAAGCCGAAAGGGAACAAACCAAAAAAGAAATTAGAGAAACGTCAAAGAGAACCTCGATTCGCGTTCACCACTAAGAGCGAAGTTGATCATCTTGAAGATGGATACAGATGGAGAAAATATGGGCAGAAGGCAGTCAAGAACAGCCCTTATCCAAGAAGTTATTATCGATGCACCAGCCAGAAATGCATGGTGAAAAAACGTGTAGAGAGATCATTCCAAGATTCATCGATCGTGATTACAACATACGAAGGCCGACATAATCATCATAGTCCAGCGACTCTTCGAGGGAATGCTCTCAGAATGTCAGCACCTTCTATGTTACCATCACCACAATCAGTTCCAAGCTTCCCTCAAGAGCTACTAGTTCAAGCTCCCTCTATAAATAACAATCAAGGTAACACAGGTTCCATGCATACCCAAAACCTAACTACACTTCATCAGCTCCAATTTCCTGACTATGGCCTTTTGCAAGACATGGTTCCCTCATTCACCCATGAACAACATCAACAACAACAGTGA |
|  | MSDKTLDHHHHHDPYHNNDQTNISGTRFPLSSDPSSSFDQMATNIPYSSSNYLHGFDPVHMSFTESLYGSMDYNTLKMAFDMSCSPSEVLSSVDGGGAGQKTGGLGESILEGGDIPMTPNSSVSNSSSNEKDAAEEETTKSTKKDEKPKGNKPKKKLEKRQREPRFAFTTKSEVDHLEDGYRWRKYGQKAVKNSPYPRSYYRCTSQKCMVKKRVERSFQDSSIVITTYEGRHNHHSPATLRGNALRMSAPSMLPSPQSVPSFPQELLVQAPSINNNQGNTGSMHTQNLTTLHQLQFPDYGLLQDMVPSFTHEQHQQQQ |
| AktWRKY11 | ATGGAGATAATTGAGATGGATTACTCGAAAGTGATTTCATCACAGACACAGAGCCAGAGTAGAACGAGAACAGGTCATGCACGTTTCAGAAGGGGTCCTTCGGTTTATCCCAAACAGCAAAACCAAGAGAGCAATTACGAACCCCGTTTGGTGACGACCCATCAACACGAAGAGGAGAATAAACCAAAATCTTCTGGATTTAAGATTTATAATTGCCCGACGCCGCCTATCCAACAAATAAACTATATAACACAGGTTTCCTTGGAAGAACTACAGCCTTCATCTTCGTTGAAGAGAAATTTTAGCTCTTCTGGAGATGCTGTTACCAAGTGCGGAGGAAGTGCTGGGAAGTATCATTGTTCGAATTCGAAGAGAAGGAAATCGAGAGTGAAGAGGGTGGTGACAGTGCCAGCGATTAGCTTGAAGATGACTGATATTCCAGCAGACGATTTTACTTGGAGGAAATACGGTCAAAAGCCCATCAAAGGATCCCCACATCCAAGGGGGTACTACAAGTGCAGTAGCATGAGGGGTTGCCCCGCACGAAAACGCGTGGAAAGAGCTTCAGATGATCCAAAAATGCTGATCGTCACCTACGAAGACGAGCACAACCACTCTCAATCGGTGGGGGAACCAACTGCTGTTATACTAGAATCTTCCTAG |
|  | MEIIEMDYSKVISSQTQSQSRTRTGHARFRRGPSVYPKQQNQESNYEPRLVTTHQHEEENKPKSSGFKIYNCPTPPIQQINYITQVSLEELQPSSSLKRNFSSSGDAVTKCGGSAGKYHCSNSKRRKSRVKRVVTVPAISLKMTDIPADDFTWRKYGQKPIKGSPHPRGYYKCSSMRGCPARKRVERASDDPKMLIVTYEDEHNHSQSVGEPTAVILESS |
| AktWRKY12 | ATGGAAGGAGATCGAGAAGCTTCTCGCGGTTGTTACGAGCTTGGGATCTCGTTTTCTAGTCCTCCACAAGCAATCCATGAGATGGGGTTTGTTCAGTTTGAAGAAAATCAGGGTTTGAGCTTCTTAGTCCCTCCCTCTCAGTCTTCTCAGGTTTCTCTGTCTTTTAACAACAGCAACAACAACAACAACGCTATTGGATTTAGTTGTAGCGATCTTGTGTCTAGGTCTTCTTGGAATAATGAACAGGTCGGAACTTTGGATCCAAAGGTTGTTAATGATGATAATTGTACTGGTAATGTTAACGATGGCAACAATTCATGGTGGAGGAATTCATCTTCAGAGAAGAGCAAAGTAAAGGTGAGGAGGAAGCTTAGAGAACCAAGGTTTTGTTTCCAAACTAGGAGTGATGTAGATGTACTAGATGATGGTTACAAATGGAGGAAATACGGCCAAAAAGTAGTCAAGAACAGTCTTCATCCAAGAAGTTATTATCGTTGCACACATAATAATTGTCGAGTGAAGAAGAGGGTTGAACGATTATCTGAAGATTGTCGAATGGTGATTACGACTTATGAAGGCAGACACACCCACTCCCCTTGTGATGATTCAAATTCATCTGAACAAGAATGCTTCAGCTCATTTTAA |
|  | MEGDREASRGCYELGISFSSPPQAIHEMGFVQFEENQGLSFLVPPSQSSQVSLSFNNSNNNNNAIGFSCSDLVSRSSWNNEQVGTLDPKVVNDDNCTGNVNDGNNSWWRNSSSEKSKVKVRRKLREPRFCFQTRSDVDVLDDGYKWRKYGQKVVKNSLHPRSYYRCTHNNCRVKKRVERLSEDCRMVITTYEGRHTHSPCDDSNSSEQECFSSF |
| AktWRKY13 | ATGTCTTCATCTTCTCAAGTTATGCTAAACCAGGGCTTATTGGAGGATCAAGAGATGTCATCCTCTCAAATGGGTTTCTACGCAAATGGATCTTCTGTCCCTCCCAACTGGACCTTTCATCCACTTGGGTATCTTCAACCCAACAGAAGTACTTCTATGGGCACAAGAGATGTTACTTCTCATCTAAGTGAAAATCTCTCTTCCACAGCCCCAAAGCAAAGAGACGTAACTTCTCATCTTGATGAATTTGAAGAAGGTCCTTCACTTCGAAGATCTAACACTAATCTATGGGCTTGGGGAGAAGTGGAAGGATGGTTGGGAAGTAAGAGATATGGTGGAGATAATCATGTAGGAGTTGGTGCAATGAAGATGAAGAAGGTGACGAAGGCAAGGAGAAAAATTAGAGAGCCAAGGTTTTGCTTCAAGACTATGAGTGACGTGGATGTCTTGGACGATGGTTACAAGTGGAGGAAGTACGGTCAGAAAGTAGTGAAGAACACACAACATCCAAGGAGCTACTACCGTTGTACACAAGACAATTGTCGCGTGAAGAAGAGAGTGGAGCGATTAGCGGAGGACCCACGGAATGTGATCACGACTTATGAAGGGAGACATGCACATTCTCCATCACATGATCAGGAAAATTCCCAAGCTTCTTCCCAAATCAATAATTTCTTCTGGTAG |
|  | MSSSSQVMLNQGLLEDQEMSSSQMGFYANGSSVPPNWTFHPLGYLQPNRSTSMGTRDVTSHLSENLSSTAPKQRDVTSHLDEFEEGPSLRRSNTNLWAWGEVEGWLGSKRYGGDNHVGVGAMKMKKVTKARRKIREPRFCFKTMSDVDVLDDGYKWRKYGQKVVKNTQHPRSYYRCTQDNCRVKKRVERLAEDPRNVITTYEGRHAHSPSHDQENSQASSQINNFFW |
| AktWRKY17 | ATGGCGGTTGACCTTCTAGGGTATTCGAAGATGGAAGAACAGATCGCGATTCAAGAAGCGGCCACTGCAGGATTGAGAAGCATGGACCATCTGATCCGCATGTTATCTCATCAAAATCAACAAAACCAACAAAATCAGCAAATCAATCAGTTGGATTGCAGAGAAATTACTGATTTTACAGTATCTAAGTTCAAGAAAGTGATTTCGATCTTGAATCGGACTGGTCACGCTAGGTTTCGTCGCGGCCCTTCATGTTCTTCTTCATCATCTTTGGCTCCCCAAACTCAAGCTCAAACCCTAACCCTAACAACACCAACAGTTACACCAAATACGGTTTCGATTGTTCAACCACAACCGCAAATTTCACAAGGCCTAACCCTAGATTTCACAAAACCTAATCTGGTTAATTCAAACCCCAACCCTAATCTAAGCGAGGTTTCGACAAGCCAGTTTACCAAGGACAGTTTCAACTTATCTCCTCCGATTTCTACAACTTCATCGTTCATGTCTTCAATCACCGGAGACGGTAGCGTCTCTAACGGAAAACAAGGATCTTCTCTCTTCATTGCTCCGGCATCGACTGTTTCCGCCGGAAAACCGCCTCTTTCCTCTTCTTATAAGAAAAGATGCCACGAACACGGTCACTCCGACACTGTTTCTGCCAAATACACCGTTTCAGGCGGCCGTTGCCATTGTTCCAAGAGAAGGAAATCTCGAGTGAAGAAAACCATTAGAGTCCCTGCAATCAGTTCAAAGATGGCTGATATTCCTTCAGACGAATACTCATGGAGAAAATACGGACAGAAACCTATCAAGGGCTCACCTTATCCACGGGGATATTATAAGTGTAGTAGCGTAAGAGGATGCCCAGCAAGGAAACACGTGGAACGAGCACCAGATGATCCAACGATGCTGATCGTAACATACGAAGGAGAGCACCGTCACTCACAACAAACTCCCCTATCTGAACCAACCTCACTTGGAGGTGGTGTTAGTCTCATGTTCGAGTCTTAG |
|  | MAVDLLGYSKMEEQIAIQEAATAGLRSMDHLIRMLSHQNQQNQQNQQINQLDCREITDFTVSKFKKVISILNRTGHARFRRGPSCSSSSSLAPQTQAQTLTLTTPTVTPNTVSIVQPQPQISQGLTLDFTKPNLVNSNPNPNLSEVSTSQFTKDSFNLSPPISTTSSFMSSITGDGSVSNGKQGSSLFIAPASTVSAGKPPLSSSYKKRCHEHGHSDTVSAKYTVSGGRCHCSKRRKSRVKKTIRVPAISSKMADIPSDEYSWRKYGQKPIKGSPYPRGYYKCSSVRGCPARKHVERAPDDPTMLIVTYEGEHRHSQQTPLSEPTSLGGGVSLMFES |
| AktWRKY18 | ATGGAACTAAGTTTGTTGGATACTTCTCTGAATTTCGACCTCAACTTGAATCCTATACCGAGTCGATGTGGAACTCCTAAGAAGGAATTGCAAAGTGATTTCATCGATGTAGGAATGAAACTTTCAGAAAAGGAAAAGGCTGGTCTATTAGTACAGGAGTTGAATCGAATTGCTGCAGAGAATAAGAAGCTGAGTGAGATGGTGACAGTTGTATGTGAAAAATATAACATTTTAAAGAACCATATGATGGACTTGATGAAAAACAATTCCAATAAAGGGTCTGCGATATCAATGAAGAGGAAGGCTGACAGCATTGATAATTCCCTTAATAATGGGATGATTGCCAATATGGAGAGTTACTCTAGTGATGAAGGATCCTGTAAGAAATCCAGAGGAGATCTCACTACAAAGATCTCGAAGGTTTATGTAAAGAGCGATCCATCCGATACTCGACTTGTAGTGAAGGATGAATATCAATGGAGGAAATATGGACAAAAAGTCACTAGAGATAACCCATCTCCTAGAGCTTACTTCAAGTGCTCCTTTGCCCCAAGTTGCCCAGTCAAAAAGAAGGTACAAAGAAGTCTTGAAGATCAATCTATCTTAGTGGCAACCTATGAAGGGGAGCACAACCACCCGCACCCTTCTCAAGTCGAGGAAGCAATGGGTTCAAGCCGTGGAGTAGCCCTTGGTTCGGTTCCTTGTTCTGCCTCCATCAACTCTTCAGGCCCCACTATAACACTTGATCTGACTCAACCCAGATTATCTAATGATCCCAAAAAACCTAGAGGGGATATTGAATCACCGGTGTTCAAACAGTTTTTGGTTGAGCAGATGGCTTCTTCGTTGTCGAAAGATCCAGGGTTCACAGCAGCACTTGCAGCCGCGATTTCTGGAAAAATTATTCAACATTCTTCGACTGAAAAATGGTAA |
|  | MELSLLDTSLNFDLNLNPIPSRCGTPKKELQSDFIDVGMKLSEKEKAGLLVQELNRIAAENKKLSEMVTVVCEKYNILKNHMMDLMKNNSNKGSAISMKRKADSIDNSLNNGMIANMESYSSDEGSCKKSRGDLTTKISKVYVKSDPSDTRLVVKDEYQWRKYGQKVTRDNPSPRAYFKCSFAPSCPVKKKVQRSLEDQSILVATYEGEHNHPHPSQVEEAMGSSRGVALGSVPCSASINSSGPTITLDLTQPRLSNDPKKPRGDIESPVFKQFLVEQMASSLSKDPGFTAALAAAISGKIIQHSSTEKW |
| AktWRKY19 | atggaagtaaatgtgaaatcgaaccttacaAATCATGAAAGAAGATCTCTTGAAGCTGAAACCAGAGCTTACGAGCTTCAATTTGAGAGCTTTAATGGTGGAAACGTTCTGAATTCGAGTTCTAATAATAATGCTTTAATGGGAGAAAATCCAAAAAGGAACGATTTTTCTGTAAAATCGGATTCTAGCGATTTCTCTAGTTCCAGATCGAATGCTGCTAGGTATAAATTGATGACTCCAGCTAGACTTCCAATCTCAAGGTCTCCATGTTTGACGATCTCTTCAGGTCTGAGCCCTACTACGTTGCTTGATACTCCTGTTCTTCTCTCGAACATGAAGGCAGAACCTTCACCGACCACTGGTGCCTTCAGCAAACCCCAAATCATGCATGACACACTCGTTTCTGATGAGTTCTCTTCTCCGAGGGACACATCCAATAGCTGTACTTATGATGAAAGAAACTATAATGACTTTAAGTTTAAATCTCACACTAGATTGAGCCCAGGTTCAAGCCTATCATTGGGACCTTTGGCTTCTACAGGGTTGAACCACCAGCACTGTGAGTCATTTGTTCGAGTCGAAGCTCAACACGAGACTCATACATTTGCATCCTCACCTTCTATTAGAAATGAGAAGATGTTAGCTTCCTCACATGAATTAACTTTATCTGTAACTGCTCCAAACCCATCAGTTCACATGGTGACTTCAAAAGCTAGTGCACCCAGTGAAGTTGCTTCTGATGAGTTGTCCCAGAGACAGGGTTCTCAAAATGGGATCCAAGCAATGCAATCTGATCATAGAGGAACTAATTCTTTGATAACGACTGAGAAATCATCGGAAGATGGATATAGTTGGCGAAAATATGGGCAGAAACATGTTAAAGGATGTGAGTTTCCACGGAGCTATTATAAATGTACTCATCCTTACTGCCAAGTGAAAAAGCAGTTGGAACGGTCTCACGATGGGCAGATTACAGATATAATCTACAAAGGAAATCACGATCATCCTAAACCTCAGCCTAGCCGTCGGATGACAGTTGGAACAATTGTGTCAACCCAAGAAGAAGGATCTGCTAGAGACAAGTCATCAAATGCGAATACCCAGACAAATCATCAAATTGAGCAAAATGTTACCCCTGATCTTTCTCCTGTTATGGCCAGTGGTGATAATGTAGAAGGTGTTCAGGATGATGATTATGATCCAGAGTCTAAGCGCAGGAAGAAAGACATTGGCGGCGTAGATGTTACAACAGGGGGTAAAATAACCGGGGAATCACGCCTTGTTGTTCAAACTCTAAGTGAAATTGATATACTGGATGATGGGTACCGCTGGCGCAAATATGGGCAGAAAGTGGTGAGAGGGAATCCTAATCCAAGGAGTTACTACAAATGCACAAATGTTGGATGCCCAGTTAGAAAACATGTGGAGAGGGCATCACATGATCCAAAAGCAGTTATAACTACATATGAGGGAAAACATAACCATGATGTACCAGCTGCAAGGACTAGCGCCCATGATGCATCAGTGTCAAAAGTTAATAATACAGCTTCAAATGACATGTTAATGATTAGATCTGAAGAAATTGACACCATTAGCCTTGATCTTGGGGTCGGGATTAGCTCAATTCCTGAAAATAGATCAAATGAGAAGCAGCAAACACTGGTGGTGGAACCTGCTCATAGCCAATCCCACATCGCTGATTCTAATTGTAGTAATATGAACCAACGAGCCCCAGTTTTGGCATATTGTGGTAGTTTGAAAGATGGCACAGATCAGTATGCGAAAAGGGAGGGCCGGGTTGAAAGTTTTAGATTTGAGACTCTACCATTAAATCATAACTATAACCCATACCTGCAGAGTGTGGGAAGATTAATAACAGGTCCATAA |
|  | MEVNVKSNLTNHERRSLEAETRAYELQFESFNGGNVLNSSSNNNALMGENPKRNDFSVKSDSSDFSSSRSNAARYKLMTPARLPISRSPCLTISSGLSPTTLLDTPVLLSNMKAEPSPTTGAFSKPQIMHDTLVSDEFSSPRDTSNSCTYDERNYNDFKFKSHTRLSPGSSLSLGPLASTGLNHQHCESFVRVEAQHETHTFASSPSIRNEKMLASSHELTLSVTAPNPSVHMVTSKASAPSEVASDELSQRQGSQNGIQAMQSDHRGTNSLITTEKSSEDGYSWRKYGQKHVKGCEFPRSYYKCTHPYCQVKKQLERSHDGQITDIIYKGNHDHPKPQPSRRMTVGTIVSTQEEGSARDKSSNANTQTNHQIEQNVTPDLSPVMASGDNVEGVQDDDYDPESKRRKKDIGGVDVTTGGKITGESRLVVQTLSEIDILDDGYRWRKYGQKVVRGNPNPRSYYKCTNVGCPVRKHVERASHDPKAVITTYEGKHNHDVPAARTSAHDASVSKVNNTASNDMLMIRSEEIDTISLDLGVGISSIPENRSNEKQQTLVVEPAHSQSHIADSNCSNMNQRAPVLAYCGSLKDGTDQYAKREGRVESFRFETLPLNHNYNPYLQSVGRLITGP |
| AktWRKY20 | ATGGCAGTTGGTAGAATTCTATTCCAAGAAGAAAGATCTGACGTGTTGTCTTCTTTAAACAGCACAGACGATAAGTCATCAAATGGACATGATGCTTCGCAATCAAACAGGATTGATGATGAGGCTGCGGATGATAATCCGGAGTCTAACCGCAGCTCCCTTAAGTTCAATGAGGAGACCACCAGCAATGCTGACGCGGGGTTGAACCAGCAACAATGCAAGTCATCTGTGCAAGTCGAAGATCAAAGCGAGTCTCAGATATTTCTATCCTTACCTTCAGTTAATGAGACTATGCCCATATCCTCCCATGAGATGACTCTAATGGTTCCAAACCCACCAGTTTACATGGTTACCACAAGTGGTATTGTACCTATTGAAGTTGACTCCGATGAGTTGGGGCAAGGAGAGGGCTCAGACGGTGTGGTTCAACCAGTGCCCTTAAATCATAAGGAAACTAGTCCATCAATATCACTTGAGAGATTGTTGGAAAATGCGTATTACTGGCGAAAATATGGACAGAAGAATATTAAAGGAAGTGAATTTCCGCGAAGCTATTACAGATGCACACATCCTAACTGCCAAATGAAAATGCAATTTGTACGATCCCATGATGGGCGGGTTATAGAGATTATTTGTCGGGGAAGGCACAATCATCCTAAACCTCAACCTTGTAGTCGATTGGCAATTGGTGCTATCTTGTCTATCCATGAAGAAACATTTAATGAAATTTCTTCTCTAAGCAACACTGAAGATAAATCAGCAAATGGACGTGGCCAAACATCTCTTCATGAGAAGCGAAATGGTACCCCTGATCTTTCTCTTGTCACAGTCAGTGATGATGATGGCGAAGGTGCAGGTGGGGAATTGAACAGGATTGGCGACGAGGTTGAAGATGATAATGGTCCAGAGTCTAAGCGCAGGAGGAAAGACACGGGTGATGTAGATTTTGCTCAATTTGGTAGGGCAATCAAGGAAACACGTGTTGTTGTTGAAACTATAAGTGAATCTGATGTAGTGGATGATGGATACCGCTGGTGCAAATATGGGCGGAAAATGGTTAAAGGGAATCCTTATCCGAGGAACTACTTCAGATGCTCAAATACGGGATGCTCAGTTAAGAAGCACGTGGGGAGGGCATTACATAATCCGAAAGCACTAATAACCACATATGAGGGAAATCATAACCATGATCAACCTCCTCCAAGGATTCCTAACCAGGACACAGCTGGGCCCATAGTTACAGCTTCAGATGGCACCTTAGAAACTAGATCAGAACTTGATACAATTATCCTTGAAGTTGGGGCCGGGCTGTGCTCGAGTCCTGAAAATATACTAGACACCGAACCCATCCAAAGTCAATCTCACATTGCCGATCCTGATCGTGTCGAGTTGATTGAAGCAGCCCCAGTTTCAGCACATAATGATGATTTAAACGGAAGCGCAGATGGAGACAGATCAAGGGATCAGGGTGAGAGCTTTTACTTCAACACTCCACCTTTGGACCAATCATCTGTGACTCAAACATAG |
|  | MAVGRILFQEERSDVLSSLNSTDDKSSNGHDASQSNRIDDEAADDNPESNRSSLKFNEETTSNADAGLNQQQCKSSVQVEDQSESQIFLSLPSVNETMPISSHEMTLMVPNPPVYMVTTSGIVPIEVDSDELGQGEGSDGVVQPVPLNHKETSPSISLERLLENAYYWRKYGQKNIKGSEFPRSYYRCTHPNCQMKMQFVRSHDGRVIEIICRGRHNHPKPQPCSRLAIGAILSIHEETFNEISSLSNTEDKSANGRGQTSLHEKRNGTPDLSLVTVSDDDGEGAGGELNRIGDEVEDDNGPESKRRRKDTGDVDFAQFGRAIKETRVVVETISESDVVDDGYRWCKYGRKMVKGNPYPRNYFRCSNTGCSVKKHVGRALHNPKALITTYEGNHNHDQPPPRIPNQDTAGPIVTASDGTLETRSELDTIILEVGAGLCSSPENILDTEPIQSQSHIADPDRVELIEAAPVSAHNDDLNGSADGDRSRDQGESFYFNTPPLDQSSVTQT |
| AktWRKY21 | ATGGATTCAACAACAGCTAAGAATTCTATTCAGCTTACCCAGAAAATGTTCTTAGAAAACCCATCATTAGAAATGGGTTCACTTTCTAAGAATCCTCACCAACTTTCCCAACAAACACCACCAACACACTATCAACTCCTCCAACAACAACAACAACATCAGCAACAACAACACAGGTTTCAAATTCAGCAACAACAACAGATGAAATTCCAAGCCGAGATGATGTATAGGAGGAGCAATAGCGGCATAAACCTCAAGTTTGATAGCTCTAGTTGCACACCCACCATGTCATCAACTAGATCTTTCATCTCATCTCTAAGCATGGATGGAAGTGTGGCTAACATGGATGGAAAAGCCTTTCATTTGATCGGTGCACCACAGTCGTCGGATTGGAACTCGAACCAGACTCCAAAGAGGAGGTGCTCTGGTAAGGGAGAAGATGGAAGTGTTAAATGTGGAAGTAGTGGTAGATGCCACTGTTCAAAGAGGAGGAAACTGAGGGTGAAGAGATCTATCAAGGTGCCTGCTATTAGTAACAAACTTGCAGATATCCCTCCCGATGAGTATTCGTGGAGGAAGTATGGGCAGAAGCCAATTAAGGGTTCTCCACACCCTAGGGGATACTATAAATGTAGCAGCATGAGAGGTTGCCCAGCGAGGAAGCATGTCGAGAGGTGCTTGGAAGATCCCTCAATGCTTATCGTCACTTACGAAGGTGAGCACAACCACTCTAGGCTGCTCTCGCAATCTGCCCACACAGAGACCATATAG |
|  | MDSTTAKNSIQLTQKMFLENPSLEMGSLSKNPHQLSQQTPPTHYQLLQQQQQHQQQQHRFQIQQQQQMKFQAEMMYRRSNSGINLKFDSSSCTPTMSSTRSFISSLSMDGSVANMDGKAFHLIGAPQSSDWNSNQTPKRRCSGKGEDGSVKCGSSGRCHCSKRRKLRVKRSIKVPAISNKLADIPPDEYSWRKYGQKPIKGSPHPRGYYKCSSMRGCPARKHVERCLEDPSMLIVTYEGEHNHSRLLSQSAHTETI |
| AktWRKY23 | ATGGAGAAGAAAGAATCAATGGAACCAGAGAATTCGATCGAGTTATCGACATTTTCCGATCAGATTCCCACTAATTACTATCCATCATCAAGCATCTTCGACATGTGTGAAGACGAAAAAGGCTCTCTAGGCTTCATGGACTTACTTGGTATCCATGATTTTAATCATTCTATATTCGATTTGCTACAACCATCATCAGTAGCGATACCTCCACCAACAACAACCACAACTTCCTTACTTCCAGAGTCATCTGAGGTGTTGAATTTTCCGACGACGCCGAACTCTTCATCGTCTATCGAAGCAACAGCCAACGATGAGCAAACGAAGGTAGCTGAAGAAGAAGATGAACAAGAGAACACTAAGAATCAATCAAAACCCAAAACCAAGAATAAAAAACGACAGAGGGAACCGAGATTTGCGTTCATGACAAAGAGCGAGGTTGATCATCTTGAAGATGGGTACAGATGGAGAAAATACGGACAAAAAGCTGTGAAAAATAGCCCTTTTGCTAGGAGCTACTACCGTTGCACCAATGGCACATGTAGTGTGAAGAAGCGTGTGGAACGATCGTCCGAGGATTCGACGATCGTCGTAACAACCTATGAAGGCCAACACACTCATCCTAGCCCAATCATGCCACGAGGAAGCCATGCCGTAGTCCCACAAGTTTCGGGCAGCTTTGGGGCCGTCGACGCCACCACCGCTTCCTTTGCTACTTCGATGCAAATGACACAAACTCATTACCAACAACAACCCTATTTCCATAGCTTAATACCTCTTTCAAATTTTGGTTCGTCGACTTCACTTTCTTCATCTTTACCTACTTTTCTTCATGAGAGACGTTTTTGCACTCCATCAGCAACATCTTTGCTTAGAGATCATGGGCTTCTTCAAGATATCGTTCCGTCCGATATGCGTAAAGAGGAGTAG |
|  | MEKKESMEPENSIELSTFSDQIPTNYYPSSSIFDMCEDEKGSLGFMDLLGIHDFNHSIFDLLQPSSVAIPPPTTTTTSLLPESSEVLNFPTTPNSSSSIEATANDEQTKVAEEEDEQENTKNQSKPKTKNKKRQREPRFAFMTKSEVDHLEDGYRWRKYGQKAVKNSPFARSYYRCTNGTCSVKKRVERSSEDSTIVVTTYEGQHTHPSPIMPRGSHAVVPQVSGSFGAVDATTASFATSMQMTQTHYQQQPYFHSLIPLSNFGSSTSLSSSLPTFLHERRFCTPSATSLLRDHGLLQDIVPSDMRKEE |
| AktWRKY25 | ATGGAAGAAACAGAAGGAAGTGGTGGTGGGGCAATTGGTTTGGATGCCCCAATTGAGATTTCTAGGGATTGGAAGCCTCCATCAATGTGTATTAATGGAGGCAGTATTGCTGAGAGAAGGGCTGCGAGGTGCGGATTCAATGCCCCAAGGATCAATACAGCTCGGTTTAGATCCATTAGTCCGTTGTCGTCGCCATCGGTTCATTCACCTTATCTCACGATACCGTCGGGTATAAGTCCCACAACATTGCTTGATTCCCCTGTTATGCTTCCAAATTCTCAGGCACAACCATCTCCAACAACTGGAACTTTTCCATTGCCCTCTCTGATTCATGGGAGTACAATGCCAATTTCTTCAATTTCTAATTCAGAAAAAGACAAGGGCGAGGATGCTGATTCCCCATTCATGTTCAAGCCTCATTTGGATGCTTTTTCTTTGCAATGCCTTTCCGGTGCAGAAAATAAGGTTTCTTCTCTTTCCAATGAGGCTCAGGATATGGGGATGGATAATGTCAACCATCAAGTTCTCGTTCCAGTGCAACCACAAGTTGATTTCGAGTGTCAAGCAGGGTTTTTGAATCAAAAAGTGATAAAGAATTCTCCCTCTGATATAGAAGTATCCAACGGTATGATTGTTAATTCAAAGTGCATTGCTTTGCAAACACTGGATTCTGGTTTTGCCAGCGATCAAGCACCTCAACATGAGGAGCAGTTGCACGTAGAGGATACTACAGGCTCACAACAGCTTTTGGAAGGGGACCAGAGAGAGGCATATCTACCCATGGCAATAGGTAAACCATCAGAAGACGGATATAATTGGAGAAAATACGGCCAAAAACAGGTAAAAGGAAGTGAATTTCCTCGGAGCTACTACAAATGCACTCATCCAAATTGTCAGGTGAAGAAAAAGATAGAGCGTTCACATGATGGTCAGATAACCGAAATTATTTATAAGGGTGCTCATAATCATCCAAAGCCTCAGCCTAGTCGACGATCGGCACTTGGATCAGCATTCCCTTTTAATGAGATTTCAGAGACTGGTGAGGGCTCTGGATCTTATGTCAACAAATTTCAAGGAGGATATACTTGGAGAAGTACTCTACAGGGTTCTAAGGATATTAAAGTTAGCTCAGATTGGGTAGCTGATGGTCTGGAAAGAACATCGTCTACATCTGTTTTAACTGAGCATTCTGATCCATTATCAACTGCGCAAGGGAAACACTTAGATATACTTGAATCTGCTGATACTCCAGAGCTTTCATCTACACTTGCTAGTCATAATGAAGATGAAGATAGGGCCACACAGGGAAGTATATCACTTGGTGAAGATGATGATGATGAGTCAGAGTCAAAAAGAAGGAAGAGAGATTGCGGTTTGATTGAAACAAATTTGGTATCCAGAGCTGCTCGTGAACCGAGAGTTGTTGTCCAAACAGACAGTGAAGTGGACATACTTGATGATGGATACCGCTGGCGCAAGTACGGGCAAAAAGTGGTCAAAGGGAATCCAAATCCTAGGAGCTACTACAAATGCACGAGTGCTGGATGCTCTGTGAGAAAGCATGTGGAAAGAGCCTCACATGATTTGAAATATGTGATCACCACGTATGAGGGAAAACACAACCATGAAGTACCAGCAGCCAGAAACAGTAGTCATGTCAACTCAACTAATGGAAATGTACTTCCATCCACCACAAATGCTCAAGCCACCCTCAATTTACCGAGAAACACCAATGTCCCAAAACCCGAACCACAAGTTCAAGATTTTGTACCTCATTTTGAGAGAAAACCTAACCTCAGTAATGACTTCTTTAAACCCATTAATCTAGGTGAGCTTGCTAGTGATATGAAGTTTGGGGCTATGAAGCTTCCACCCTTTCAGACTGCCATGCCTTCTTTTGGTGGATTCAATGCTAACCACAGCGAAGCTCTTCAATCAGCTTCTATTGCTCATATTGCTTCTGAATTCCCAATTACATTGCCAATGAATCTTCCCCTCTCAACAAACCTGGCAATGTCGGGTTTTAATTTTAATAGCATCGGAAAGCGTGTTTGTCCTACTCAGACTTACCTTGGGGAACAGCAACCTAAGGAAGCTGATATGAGGTTCCTCAGGCCTAAACAGGAGCTGAAGGATGAAACTGTTTTTGAGACCCTTCTGCCTATTGATCATCTTGCTGGTGCGTCATCGTCTGTATATCACCAGATTATGAGAGGTTTTCCATTATAG |
|  | MEETEGSGGGAIGLDAPIEISRDWKPPSMCINGGSIAERRAARCGFNAPRINTARFRSISPLSSPSVHSPYLTIPSGISPTTLLDSPVMLPNSQAQPSPTTGTFPLPSLIHGSTMPISSISNSEKDKGEDADSPFMFKPHLDAFSLQCLSGAENKVSSLSNEAQDMGMDNVNHQVLVPVQPQVDFECQAGFLNQKVIKNSPSDIEVSNGMIVNSKCIALQTLDSGFASDQAPQHEEQLHVEDTTGSQQLLEGDQREAYLPMAIGKPSEDGYNWRKYGQKQVKGSEFPRSYYKCTHPNCQVKKKIERSHDGQITEIIYKGAHNHPKPQPSRRSALGSAFPFNEISETGEGSGSYVNKFQGGYTWRSTLQGSKDIKVSSDWVADGLERTSSTSVLTEHSDPLSTAQGKHLDILESADTPELSSTLASHNEDEDRATQGSISLGEDDDDESESKRRKRDCGLIETNLVSRAAREPRVVVQTDSEVDILDDGYRWRKYGQKVVKGNPNPRSYYKCTSAGCSVRKHVERASHDLKYVITTYEGKHNHEVPAARNSSHVNSTNGNVLPSTTNAQATLNLPRNTNVPKPEPQVQDFVPHFERKPNLSNDFFKPINLGELASDMKFGAMKLPPFQTAMPSFGGFNANHSEALQSASIAHIASEFPITLPMNLPLSTNLAMSGFNFNSIGKRVCPTQTYLGEQQPKEADMRFLRPKQELKDETVFETLLPIDHLAGASSSVYHQIMRGFPL |
| AktWRKY26 | ATGGCTTCTTCACCTGGAAGTTTAGAGACTTCTGCTAATTCTCATTCAAACTTTTCTTTTTCGTCCCAATTTTTAAATCCCTCTTTCACTGATCTTCTCTCGGGAGGAGGAGAAGATGATACATATAATCAAAATCAAGAGAGAATTACAATGAGTCAAGGACTATCAGATCGTATTTCTGATCGAACAGGTGGTGTTGGAGTTCCAAAATTTAAATCAATTCCTCCTCCTCAACTACCCATCTCTCCAACTTTGTTTTCTCCTTCTTCTTATTTTTCAATACCAACTGGTATCAGCCCAGCTGAACTCTTAGATTCACCTGTCCTTCTCTCATCTTCTAATATTCTACCATCTCCAACTACTGGATCATTTCCTGCTCAAGCATTCAATTGGAGGGGTAATTCTGGTGATTTCCAACAAGGTATCAAGAAAGAATACAGAAACTCCTCTGATTTCTCTTTCCAACCCCAAACAAGGCCTACTACAACTTCGTCTGGATCGTTCTTTCCGACCTCTAAGACAACAATTTCTTCGGGAGAAAGCAATAAAGGACAGCAACAACCATGGAATTTCCAAGAATCAACCAAACAGACTAATTTCTCAACTGGGAAGAACATTGTCAAATCAGAATTTGTCCCATTACATAGCTTTTCACCTGAAATTTCTACAATACAAACTAATACCCAAACCAATGGAGGACTCCAATCTGATTATAACCAATACTCTCAACCTTCTCAATCTATTAGAGAGCAAAAAAGATCAGATGATGGATATAATTGGAGAAAATACGGCCAAAAACAAGTGAAAGGAAGCGAAAATCCTCGTAGTTATTACAAGTGCACTTATCCTAATTGCCCAACTAAGAAGAAAGTTGAGAGGTCTTTAGATGGACAGATTACTGAAATAGTTTATAAGGGTAGTCATAACCATCCAAAGCCTCAATCTACTAGAAGATCATCATCTTCATCTCATCAAATTCAAGCTTCTTTGGCCCCTTCTTTAGAAATATCTGATCATTTTGCATCACATGGGACTACACAAATGGAATCAGTTGCGACGCCTGAGAATTCTTCGATCTCTATTGGAGATGATGATGTCGATCGAAGCTCTCAGAGGAGTAAGTCAGGTGGTGATGAATTTGATGAAGATGAACCAGAGGCCAAAAGATGGAAGAAAGAGAATGAAAATGAAGGTATATCAGCTTCAGCAAATAGAACTGTGAGGGAACCTAGAGTTGTAGTTCAAACAACTAGTGACATTGACATTCTTGATGATGGATATAGGTGGAGGAAATATGGGCAGAAAGTAGTAAAGGGAAATCCCAACCCAAGGAGTTACTACAAGTGTACAAGTACTGGATGCATGGTACGAAAGCACGTCGAGAGAGCATCCCATGATCTTAGAGCGGTGATCACAACATACGAGGGGAAGCATAATCATGATGTTCCTGCAGCTCGTGGCAGTGGCAGCCATTCGATTAATAGGCCATTGCCGGATAACAACAACAATAACAATAATGTAGCAATGGCAATAAGGCCTTCGATCACGAACAATCACTCTAACAATATGATGAGCAACATGCTTCGGAATCCGAGAATACAAATGTCGGACGGGCAAGCGCCCTTCACACTAGAGATGCTACAGAGCCCAGGGAGTTTTGGATTTGGAAACTCGATGGGTTCATACATGGGTCAACACGTACAACAACAACAACAACAACAGCACACAGACAATGGGTTCCCAAAGACCAAAGAAGAACCAAAGGAAGACATGTTTATGGAGTCTCTCTTATACTGA |
|  | MASSPGSLETSANSHSNFSFSSQFLNPSFTDLLSGGGEDDTYNQNQERITMSQGLSDRISDRTGGVGVPKFKSIPPPQLPISPTLFSPSSYFSIPTGISPAELLDSPVLLSSSNILPSPTTGSFPAQAFNWRGNSGDFQQGIKKEYRNSSDFSFQPQTRPTTTSSGSFFPTSKTTISSGESNKGQQQPWNFQESTKQTNFSTGKNIVKSEFVPLHSFSPEISTIQTNTQTNGGLQSDYNQYSQPSQSIREQKRSDDGYNWRKYGQKQVKGSENPRSYYKCTYPNCPTKKKVERSLDGQITEIVYKGSHNHPKPQSTRRSSSSSHQIQASLAPSLEISDHFASHGTTQMESVATPENSSISIGDDDVDRSSQRSKSGGDEFDEDEPEAKRWKKENENEGISASANRTVREPRVVVQTTSDIDILDDGYRWRKYGQKVVKGNPNPRSYYKCTSTGCMVRKHVERASHDLRAVITTYEGKHNHDVPAARGSGSHSINRPLPDNNNNNNNVAMAIRPSITNNHSNNMMSNMLRNPRIQMSDGQAPFTLEMLQSPGSFGFGNSMGSYMGQHVQQQQQQQHTDNGFPKTKEEPKEDMFMESLLY |
| AktWRKY27 | ATGGCTACTACTACAAACATTGAAGATCCTTTCTCCTTCTTTGCTCCTTTTGATTTTAAACAAGAAGATGGTCTTCTCTGTTTTCCAGATCTCTTTGAAACAGGTACTGTTATTGAAGAACTCGAACAACTCTACAAACCCTTCTTCCCCAATTTCCAGCCCATCTCTCCTCAGAACATACCCACACCCATTCCTCCAATAATCCTACCTCAACAACAACAACAACAACAACAACAACAACAACAACAACAGCAGCAGCAACAGAGACAACAACAACAACACCAACAACCGAATCGTTCTCTTGTGGGGTCTTTTACTGCTTCTTCTAGCTCCCATCCTCAAACCCCTAGACCAAAAAGAAGGAAGAACCAACAAAAGAAGGTTGTGTGCCAAGTACCCGCAGAGGGTCTCTCTTCTGATATGTGGGCTTGGCGTAAATACGGACAGAAACCCATCAAGGGCTCTCCCTACCCAAGAGGCTATTACAGATGCAGTAGTTCAAAGGGTTGTTTAGCGAGGAAACAAGTGGAGCGAAACCGATCAGATCCTGCGATGTTCATCGTAACCTACACAGCAGAACACAACCATCCAGTCCCCACTCATCGAAACTCTCTCGCTGGAAGCACTCGTCAGAAATTCACTACATCTCCAAAAACTACAACCAACACCAAACCCTCTTGTTCATCTTCTTCTCCAACTTCAACAACAGATCTCTCTCCAACAACCCCCTTTAACAACATCAATGGATGA |
|  | MATTTNIEDPFSFFAPFDFKQEDGLLCFPDLFETGTVIEELEQLYKPFFPNFQPISPQNIPTPIPPIILPQQQQQQQQQQQQQQQQQQRQQQQHQQPNRSLVGSFTASSSSHPQTPRPKRRKNQQKKVVCQVPAEGLSSDMWAWRKYGQKPIKGSPYPRGYYRCSSSKGCLARKQVERNRSDPAMFIVTYTAEHNHPVPTHRNSLAGSTRQKFTTSPKTTTNTKPSCSSSSPTSTTDLSPTTPFNNING |
| AktWRKY28 | ATGTCTGATGAAACTAGAGATCTATACCACCATCACCCATTCAACGATGTTGATAGCCGAAACATCAGTGGGTCCACCTTCCCATTCTCTAATGATCCTTCAATTTTCAATCCAACGGTTCCTACTGCACCAGGTGGCTCGTCATCTTACAATCTACATGGATTTGATCCATCTTACATGAGCTTCACTGATTGTTTACATGGCTCTATGGACTATACTACAGTAGCAAGAGCCTTCGATTTGTCGTGTTCTTCGTCTGAAGTTTTTAGTTCTTTAGATGAAGGTCAGAAGAAGAGTGGTCTTGGAGATTCAGTGGGGACTATTCCAGTAACACTCAATTCTTCGATCTCATCGTCGTCTAATGAATCAGTTGCCGAAGAAGATTCAATAAAGAAAGATCGACAGTCGAAAGGTTTTGAAGATGGAGTTGAAAGCTCTAAGAAAGTGAACAAACCCAAAAAGAAAGGAGAGAAAAGGCAAAGAGAGCCGCGATTTGCATTCATGACTAAGAGTGAGGTGGATCATCTTGAAGATGGTTACAGATGGAGAAAATATGGGCAAAAGGCAGTCAAGAACAGTCCTTATCCAAGAAGCTATTACAGGTGCACCACTCAGAAGTGCACAGTGAAGAAAAGAGTGGAGAGATCTTTCCAAGATCCATCAATAGTGATTACCACTTATGAAGGCCAACATAACCATCAAAGCCCGGCGACAATTCGAGGAAATATGGCTGGAATGTTAGCACCTTCTATGTTAACATCACAACTGATGGGGCCGAGCTTCCATCGAGAACTACTACAGGTACCCAACCAAGGTCACACAAACTCCAATTATCCACCACACTTCACTACTCTTCAGCAGCTTCAGCTTCCTGACTATGGTCTTTTACAAGACATAGTTCCTTCATTCAACCACAAACACCAGCCATGA |
|  | MSDETRDLYHHHPFNDVDSRNISGSTFPFSNDPSIFNPTVPTAPGGSSSYNLHGFDPSYMSFTDCLHGSMDYTTVARAFDLSCSSSEVFSSLDEGQKKSGLGDSVGTIPVTLNSSISSSSNESVAEEDSIKKDRQSKGFEDGVESSKKVNKPKKKGEKRQREPRFAFMTKSEVDHLEDGYRWRKYGQKAVKNSPYPRSYYRCTTQKCTVKKRVERSFQDPSIVITTYEGQHNHQSPATIRGNMAGMLAPSMLTSQLMGPSFHRELLQVPNQGHTNSNYPPHFTTLQQLQLPDYGLLQDIVPSFNHKHQP |
| AktWRKY30 | ATGGCAAGTAGCGGATTTAGCCTTAACCACTATTTGGATCAGGAGAAAAACACAAATAGTTTAACGGTCGACAGTGGATTTGGTAACAGACACCCTATTTCGGAGCACAGCACAGCTTATCCAATGGCAAGTAGCGGATTTGACCTTAACCACAATTTTGATCAGGAGCGTCAAGACATCGGTGATGAAATAATGAACGACCTCAATTTTGAAGATCACAACATAGATAATTCAGTAGCCAGCAATGGAATTGTTGGTATACAAACTCGAAAAGTGAACACAAGATACTTAGATCAAAACCCTTTGATAATCAATGTGTTAGTCCAAGGAAAGGAACTTTTGAATCAGCTTCGAATCCATCTTAATCCCTCTTCTTCGACAGGAGAATTTCTAATAGAAAAGATGCTATCTTCCTTCAACGAGACTCTTTTGATTCTGAATCCAAGTGTTTCAGAAACAGAGATTCAACCAATAGAACCCATATCTAGTATTTTGGATTCTCCACGTTCGGTCAATGGGAGTCCACAAAGCGACGATTTGAATCGAAGAGATACGTACAAGAAGAGGGAAATGTTGCCTCGATGGTCCAAACAAGTCCGTGTTTGCTCTCGGACTGGGCTCAAAGGTGCTCTTGATGATGGTAACAGTTGGATGAAATATGGACAAAAAGACATCCTTGGAGCTAAGTACCCAAGAAACTATTTTCGATGCTTGTATCATAAACGTCAAGGGTGTTTGGCTACAAAGAAAGTTCAACGATCCGAAGATGACCCATCATTCTTCGAAGTCACTTATCGAGAAAGACACACCTGCATCCAGGGCAGAGCTAGTAAGAAGTTGGTGGGGGGCAATTGCCCCTCCAGAACTTTGGGACACCCCTATATTTTCAAGTAA |
|  | MASSGFSLNHYLDQEKNTNSLTVDSGFGNRHPISEHSTAYPMASSGFDLNHNFDQERQDIGDEIMNDLNFEDHNIDNSVASNGIVGIQTRKVNTRYLDQNPLIINVLVQGKELLNQLRIHLNPSSSTGEFLIEKMLSSFNETLLILNPSVSETEIQPIEPISSILDSPRSVNGSPQSDDLNRRDTYKKREMLPRWSKQVRVCSRTGLKGALDDGNSWMKYGQKDILGAKYPRNYFRCLYHKRQGCLATKKVQRSEDDPSFFEVTYRERHTCIQGRASKKLVGGNCPSRTLGHPYIFK |
| AktWRKY31 | ATGGACAGAGGAGGAGGACTCAGTATTGAATCTGATCCTATTGGTTTCTTTGTAACCAAGCCAACTCCCATTAATTCATTTTTGAAACCAAATAATCTCAAACGAAAATTCGATTTAATGGAACCACGTATTGATTTCTCCATGAATCTTAGTTCTAGTGATAATCAAATCACACAGCCTTCGTCCGATGAGAAACGGGTCGATGAGATGGATTTCTTCTCTGATAAAAACAGACTTCAAGATTCCAATAATAACAACTCTTCCAACATTGATGTCAAGAAGGAGAATTCTCATGGTGGAGTTCCTCCACCAAGGTTCAATTTTGATGTTAATACTGGTTTAAACCTTCTTACTGCTAATACGGGAAGCGATCAATCTACGGTGAGCGATGGGATATCGACCAACGTTGACGATAAGCGAAGAATGAATGAGCTGGTAGTTCTTCAAGTTGAGCTTGAGAGGATGAATGAAGAGAATCAGAGGTTGAAAGGGATGTTAAATCAAGTGAACAACAACTATAACAATCTTCAGATGCATTTTGTAACCATGATGCAACAACAAAATCAAAAACCTGAAATCATCCCAGAAAATGAGGTGACTGATGGAAAAATCGAGGAGAAGAAACAAGAAGGCAGTGTAGGATTAATGGTGCCTCGACAATTCATGGATCTAGGCCCTGTGGCGACAGCTGAAACCGATGAAGTTTCTCAGTCTTCTACTGAAGAAAGAAGTCATAATCGCTCTGTATCACCGCCTAACAATGCAGAAGTAGTGTCAAAGAATGGCAACAACAATGAGATTGTACCCTTTGATCAGGACAAGAGCGATTTCTCCGATGGTAGAGGAGTTGGTAGGGAAGAGAATTGGGGTCCTAACAAAGTCCCTAAATTGAATGGTTCCAAGAATGTTGAGCAATCCACCGAAGCCACTATGAGGAAAGCGCGAGTCTCAGTTCGAGCACGATCTGAAGCACCAATGATCACCGATGGATGCCAATGGAGAAAGTATGGGCAAAAGATGGCTAAGGGGAACCCGTGTCCTCGAGCTTACTATCGATGCACCATGGCAGTCGGATGCCCTGTTCGTAAACAGGTTCAAAGATGTGCCGAAGATCGAACAATATTGATTACAACTTATGAGGGCAATCATAATCATCCACTCCCTCCAGCTGCCATGGCTATGGCATCGACCACATCGGCTGCAGCATCTATGCTGCTTTCAGGATCTATGACGAGTGCCGATGGGCTAATGAACTCGAATTTCTTAACGAGGACTCTATTGCCTTGTTCGTCGAGTGTCGCCACAATCTCTGCTTCGGCTCCCTTTCCAACTGTCACATTGGACCTTACCCATACTCCCAATCCTCTTCAATTCCAAAGGCCACCAACTCAATTTCATGTCCCCTTCATGAACCCATCTCAGGCATTTACATCCACGCCCGCCCCTCAGCTTCCACAAGTTTTCGGCCAATCTTTATATAATCAATCGAAATTCTCCGGCCTTCAGCTCTCACAAGAAATAGACCCAACTCAATTTGCTCACCAACCACCACAACCACAACCACCACCACAACAACCCTCATTGGCCGACACAGTGAGTGCGGCAACAGCTGCCATCACTGCTGATCCAAACTTCACAGCAGCTCTAGCAGCGGCAATTTCATCAATAATCGGCGGAGCTCATCAAACAAACAACAACAACAATGCCAATACCAACAATAACAGTAGTGCCAACACCACTACTACCAATAGCAATAACAACAAATGA |
|  | MDRGGGLSIESDPIGFFVTKPTPINSFLKPNNLKRKFDLMEPRIDFSMNLSSSDNQITQPSSDEKRVDEMDFFSDKNRLQDSNNNNSSNIDVKKENSHGGVPPPRFNFDVNTGLNLLTANTGSDQSTVSDGISTNVDDKRRMNELVVLQVELERMNEENQRLKGMLNQVNNNYNNLQMHFVTMMQQQNQKPEIIPENEVTDGKIEEKKQEGSVGLMVPRQFMDLGPVATAETDEVSQSSTEERSHNRSVSPPNNAEVVSKNGNNNEIVPFDQDKSDFSDGRGVGREENWGPNKVPKLNGSKNVEQSTEATMRKARVSVRARSEAPMITDGCQWRKYGQKMAKGNPCPRAYYRCTMAVGCPVRKQVQRCAEDRTILITTYEGNHNHPLPPAAMAMASTTSAAASMLLSGSMTSADGLMNSNFLTRTLLPCSSSVATISASAPFPTVTLDLTHTPNPLQFQRPPTQFHVPFMNPSQAFTSTPAPQLPQVFGQSLYNQSKFSGLQLSQEIDPTQFAHQPPQPQPPPQQPSLADTVSAATAAITADPNFTAALAAAISSIIGGAHQTNNNNNANTNNNSSANTTTTNSNNNK |
| AktWRKY32 | ATGGCGGAAAACCAAAGCTTCGAAAATCCCAAACAAGAACAGAGACAAAGAGAAGAAACACAACAAGAGAAACCTCCCTCTGAGCAACAGAACACTCTCTCTTCTACCTCTCTATCCGATGTTTCTTCCTCTAAATCTGTTTTCAGGGACCAAAGTTCTCAATTAGAAACCCTAACTGTACTTCCACCGATTCTCTCCTCCGAGAACGGTCATGAATCTGATTGTCCCTCTTTTTCTCAGTTACTAGCTGGTGCGATGGCTTCTCCTGCGTCGAATTCGTCGCAGATATTATCTTCTGGAGGATTGGATGCGAAGCCGACTGTTCAAGTGGTTCTTAGTCCGTCTGGATTCAAGGGGCAATTTGGGATGTCCCATCAAGAAGTCTTGGCAAGTGTAACTGCTCAAGCTCAGGCACAGATGCAGCTTCATGTTGCATATCCATCTTCCTCATCAGAGTTATTACCTGTTTCTCTTATACAATCCATTTCTTCTACCATAACTCCAACTCCACTTAAGCAAGAACTTTCACCAGTATCTGAGGATGATAATGTCTGTACACCCGAAGCTGACGAGCAGAATTCCTCCGATCAGAAAGCTAAACCTGCTCACGTAATTATGAAGACACCCTCCACTGATGGATACAATTGGCGGAAGTATGGTCAGAAGCAGGTGAAGACTACTGATAGTTCTCGAAGTTATTACAAATGCACATTTGGTAATTGTCATGCTAAGAAGAAGGTTGAATGTTGTGATCACTCTGGTCGTATAATTGAGATCATTTATAAAGGTCAACACAATCATGACCCACCTCAAAAGATCAGATGCACTAAAGCTAGTGGTTTTGTTACGCCTAGTGGGTCTGTATCATCTGCTGGGTCCGGTGGAGGGAGTGGGACCATAGAATGCCCTAGCGAGCTTAACAATTTAGATGCATCTAGATCTAGGTTAGAACCTATATATGCTACACCTGAAAAGAAGCTAAATAGCTTAAGTGAGTGCAGTAGGGATGCTCATGTTAGGGCTGCGGAAGATCAGGGTGATGAACCATATCCAAAACGGAGGATGAAGGATGGTAGCGTGGCATATTCGAACCCTCAATTTAAAACAGTTAAGGAGCCTAAAATTGTTGTCCAAGCAGCTGGTGATGTTGGAATATCAGGAGATGGATTCAGGTGGCGCAAGTATGGGCAGAAAATGGTGAAGGGAAATCCTAACCCTAGGAGCTACTACAAATGCACATCTGCTGGATGCCCTGTCCGTAAGCATGTTGAGAGAGCTATAGAAGACACGACAACCATCATAATAACTTATGAGGGGAAACATGATCATGACATGCCCGTACCCAAGAAGCGCCATGGTCCACCAAGTACTGCTCTTCTTATTGCTGCTGCTGCAGCCATGAACAGTACACAATTGAAAACATCCGAAGCTTTAGTAAGGAAACCTAAAACCAAATGGCCTATGGAAAATGATCAAGGAGAATTGGCTGGCGAGAGGGCAATAGAACTTGGAGGTGAGAAGGTACTTGAATCGGCTCGAACTCTTCTTAGTATAGGAATTGAGCTCAAGCCCTACTGA |
|  | MAENQSFENPKQEQRQREETQQEKPPSEQQNTLSSTSLSDVSSSKSVFRDQSSQLETLTVLPPILSSENGHESDCPSFSQLLAGAMASPASNSSQILSSGGLDAKPTVQVVLSPSGFKGQFGMSHQEVLASVTAQAQAQMQLHVAYPSSSSELLPVSLIQSISSTITPTPLKQELSPVSEDDNVCTPEADEQNSSDQKAKPAHVIMKTPSTDGYNWRKYGQKQVKTTDSSRSYYKCTFGNCHAKKKVECCDHSGRIIEIIYKGQHNHDPPQKIRCTKASGFVTPSGSVSSAGSGGGSGTIECPSELNNLDASRSRLEPIYATPEKKLNSLSECSRDAHVRAAEDQGDEPYPKRRMKDGSVAYSNPQFKTVKEPKIVVQAAGDVGISGDGFRWRKYGQKMVKGNPNPRSYYKCTSAGCPVRKHVERAIEDTTTIIITYEGKHDHDMPVPKKRHGPPSTALLIAAAAAMNSTQLKTSEALVRKPKTKWPMENDQGELAGERAIELGGEKVLESARTLLSIGIELKPY |
| AktWRKY33 | atggcttcttcaggaggaagcttagaaACCTCTGCAAATTCTCATCCAACCTTCTCTTTCTCACCTCAATACATGACTTCCTCCTTCACTGATCTTCTCTCTGGAGATGGTGACACTAATAATCAAAACCAAGAGAGAATTAACATGAGCAGAGGTATTTCAGATCGAATAGCTGAGAGAACTGGTTCTGGGATTCCAAAATTCAAGTCAATTCCTCCTCCATCACTACCCATCTCTCCTCCTTCTCCTTCTCCTTCTCCTTCTTCTTACTTTGCTATACCACCTGGTCTTAGTCCAACTGATCTCTTGGATTCACCTGTCCTCCTCTCCTCTTCTAATATTCTTTCATCTCCAACTACTGGAACTTTTCCTACTCAGGCATTTAATTGGAGGAGTACTTCTGTCAATTTCCAGCAAGGTGTCAAGCAGGAAGAAAGAAACTATTCTGATTTTTCTTTCCAACCACAAACAAGGCCTACTGCTGCATCATCCGGATCGATTTTTCAGTCCTCTAAGACTGCGATTTCATCGGGAGACACCTACAAAGAACAACAACAACAACAACAACCATGGAGTTTCCAAGACTACACAAGGCAAACTGATTTCTCAGCTGGGAAGAATATTGTCAAATCCGAATTCACTCCATTGCAAAGCTTTTCGCCCGAGATTTCTACAATACAAGCTAATAATACCCAAAGTAATGGTGGACTCCAATCCAATTATAACCAATATTCTCAATCACTTAGAGAACAAAAAAGATCAGATGATGGATACAATTGGAGAAAATATGGCCAGAAACAAGTTAAAGGAAGTGAAAATCCGCGTAGTTATTATAAGTGCACTTATCCTAAATGCCCAACTAAGAAGAAAGTGGAGAGGTCTTTAGATGGACAAATTACTGAAATAGTTTACAAGGGTAGTCACAACCATCCCAAGCCTCAGTCTACTAGAAGATCATCCGCTTCTTCTCAACAAATTCAAACTTCTGTGGCCTCGGAAGTCTCCGATCACTTTGGGACACATGGGTCCACACAAATGGAGTCTGTTGTCACTCCAGAAAATTCTTCATTATCATTTGGAGATGACGATGTCGATCAAAGCTCTCAGAGGAGTAAATCAGGGGGTGATGAACCTGATGAAGATGAACCTGAGGCCAAAAGATGGAAGACAGAGTGTGAAAATGAGGGAATTTCTGCTTCAGGGAGTAGAACTGTGAGGGAACCTAGAGTTGTAGTTCAAACAACTAGTGATATTGACATTCTTGATGATGGATATAGATGGAGAAAATATGGGCAGAAAGTAGTAAAAGGGAACCCCAATCCAAGGAGTTACTACAAGTGTACAAGTGCTGGATGTCCCGTGCGAAAGCATGTTGAGAGAGCGTCCCATGATCTAAGAGCAGTGATCACAACATACGAGGGGAAGCACAACCACGATGTCCCTGCAGCCCGTGGTAGTGGTAGCCATGCAATCCATAGGCCAATGCCCGACAACAACAATAACAATTTGGCCATGGCTATAAGGCCTTCAGCCACAACTAATAACTCTAATCAAATTTCGGGCAACATGTTTCAGAATGTTAGAATGCCGACATCTGAAGAGCAAACACCCTTTACACTAGAGATGCTGCACAGTCCAGGGAGTTTCGGGTTCCCGAGATTTGGCAACTCCATGGGCAACTACATGAATCAACCACAGTACACTGATGACATGTTCCCAAAAACGAAAGAAGAACCAAGGGACGACATGTTTCTCGAGTCATTGCTATACTGA |
|  | MASSGGSLETSANSHPTFSFSPQYMTSSFTDLLSGDGDTNNQNQERINMSRGISDRIAERTGSGIPKFKSIPPPSLPISPPSPSPSPSSYFAIPPGLSPTDLLDSPVLLSSSNILSSPTTGTFPTQAFNWRSTSVNFQQGVKQEERNYSDFSFQPQTRPTAASSGSIFQSSKTAISSGDTYKEQQQQQQPWSFQDYTRQTDFSAGKNIVKSEFTPLQSFSPEISTIQANNTQSNGGLQSNYNQYSQSLREQKRSDDGYNWRKYGQKQVKGSENPRSYYKCTYPKCPTKKKVERSLDGQITEIVYKGSHNHPKPQSTRRSSASSQQIQTSVASEVSDHFGTHGSTQMESVVTPENSSLSFGDDDVDQSSQRSKSGGDEPDEDEPEAKRWKTECENEGISASGSRTVREPRVVVQTTSDIDILDDGYRWRKYGQKVVKGNPNPRSYYKCTSAGCPVRKHVERASHDLRAVITTYEGKHNHDVPAARGSGSHAIHRPMPDNNNNNLAMAIRPSATTNNSNQISGNMFQNVRMPTSEEQTPFTLEMLHSPGSFGFPRFGNSMGNYMNQPQYTDDMFPKTKEEPRDDMFLESLLY |
| AktWRKY34 | ATGGCTGGGATTTCTGATCATGTTGCCATAATTGGGGATTGGGTTCCCCCAGATCCCAGCCCTAGGACCTTTTTCTCTTCCATCATGGGTGATGATTTAGGGTCAAGATCTTTCCCACCACTTCTAGGAGGAAACAGTGGGTGTGAGGGGTCTTTCGTGGGATCGTCTAAAAATCTGAAAGAGGCAACAATTCTTGAGGAAGAAGATGGAACAGGAGTTGTTGTTTCTGGTGATTGTTTGTCAGAGTCAACCTATCTTTCCTACCAGGAATCAGGCTCCCGTGGGGGTCTTGCTGAGAGGATGGCAGCAAGAGCTGGGTTTAGTGCTCCAAGGTTGAACACTGCAAGGATTAAATCTGCTAATGTGTCCCTCTCCCCTGATGTTCGGTCACCTTACCTAACAATTCCGCCAGGTCTAAGTCCAACAACATTGCTAGATTCTCCAGTGTTCCTTTCAAATTCTCTGGATCAGCCATCTCCAACAACGGGAAAGTTATGCTTCACCCCAAGTAGTAACAGTAAAAGCTCCATGTTAGTCCCAGAAGCCCCAGACAGAAATAAGGATAGTCCGTTTGAATATGTTGATACCACATCATTTGTATTCAAGCCTCGTGTGGAATTGAGTTCCTCATTTTTCCCCAGTGCAGCAAATGAAGAAACTCCTGCTACAAATCCTCAGCAGTCTTTTGCCAATATTGAGGTGTCGGTCCATTCAGAGCGCACCCTTTGGTCACGAAGTCTAGAAGGCACCGAAGTTCACTCACAGAATATAAGCAGTTACCATCCTTGGGCAAACTTCCCCAAATCATCTGGTGAAAAGGATACCAATGGTAAAAATGCCAAATCAGAGCCAAGGGTATGTGATCCTGTTATTGCCATTGTCGAACATTCTCCAATGCTCAATGATCAGCAGGACTCAGAAGGAAATCAGAGAAGCAATGGAGAAATCAATTCCATTGTTGAAGGTGGTGCCACATCTGAGGATGGGTACAATTGGAGGAAGTATGGGCAGAAACAGGTGAAAGGCAGTGAGTATCCTCGGAGTTATTACAAGTGCACACATCCAAATTGTCAGGTCAAGAAGAAGGTGGAGCGATCTCATGAGGGCCACATTACCGAGATCATCTACAAGGGGGCCCACAATCACCCTAAACCTCCACCTAATCGCCGATCTGCTGTTGGATGCTCTGACGCACAGAATGATATGCAACTTGACATCCCTGAACAAGCCAGAGCACAGGCTGGGGCTGAGGGTGATCCTACATGGGCAAACTTACAGAGAGGAAATGTTTCCAGAGATCCTGATTGGAGGCATGAAAACCTAGAGGCAACATCATCCGCATCTGTGGGCCCTGATTTCTGTCCGCAATCTACCTCCATGCAGGCTCGAAAGGGCCCTCACTTTGAAGCCACCATTGATGTGGATGTGTCGTCCACGATGTCCAACAATGAAGACGAGAATGATCGGGGAACACAGAGAAGTGTATCATTGGGTTATGATGGCGAAGGAGATGAATCTGAGTCGAAGAGAAGAAAATTAGATGAGATGGAAGTTGGTGGAGCAGCTGCAAGAGCCATTCGTGAACCAAGAGTTGTTGTCCAAACTACTAGTGAAGTGGACATCCTTGACGATGGATACCGCTGGCGCAAGTACGGGCAAAAAGTTGTCAAAGGAAATCCAAATCCAAGGAGCTACTACAAGTGCACAAATGCAGGTTGCACTGTTAGAAAGCACGTGGAACGAGCATCTAACGACCTCAAATCAGTAATCACCACATATGAGGGGAAGCATAATCATGATGTTCCTGCTGCAAGGAGTAGCAACCATGTCAGCTCCTGTCCCTCTAGCACAGCACCCACCCAAGTTGCTCAACCCCGTATCCACAGGCCTGAACCATCCCAAATCAATGACGGCATGGTAAATTTCGATGGTCCTGCATCTGTTGGTGCATTCAGTCTGCCTGGAAGGGAGCTGGGAACCTCGCCTGGCTTCTCTTTTGGTATGCGTCAGCCAGGCCTGGCCGGCCTTGCAATGGCTGGGTTGGGCCCGGGCCTACGTAAGCTGCCTGCTTCACCACTCCATCCATATTTAGGACAGCAACGCCACATGAATGAAACAGGGCTCATAATGCCAAAAGGAGAACCAAAGAACGAGCCTGTCTCTGAGACTGGTCTGAACCTATCCGACAGTTCCTCAGTTTATCATCAGATCATGGGTAGGCTGCCTCTTGGGTCTCAGATGTAA |
|  | MAGISDHVAIIGDWVPPDPSPRTFFSSIMGDDLGSRSFPPLLGGNSGCEGSFVGSSKNLKEATILEEEDGTGVVVSGDCLSESTYLSYQESGSRGGLAERMAARAGFSAPRLNTARIKSANVSLSPDVRSPYLTIPPGLSPTTLLDSPVFLSNSLDQPSPTTGKLCFTPSSNSKSSMLVPEAPDRNKDSPFEYVDTTSFVFKPRVELSSSFFPSAANEETPATNPQQSFANIEVSVHSERTLWSRSLEGTEVHSQNISSYHPWANFPKSSGEKDTNGKNAKSEPRVCDPVIAIVEHSPMLNDQQDSEGNQRSNGEINSIVEGGATSEDGYNWRKYGQKQVKGSEYPRSYYKCTHPNCQVKKKVERSHEGHITEIIYKGAHNHPKPPPNRRSAVGCSDAQNDMQLDIPEQARAQAGAEGDPTWANLQRGNVSRDPDWRHENLEATSSASVGPDFCPQSTSMQARKGPHFEATIDVDVSSTMSNNEDENDRGTQRSVSLGYDGEGDESESKRRKLDEMEVGGAAARAIREPRVVVQTTSEVDILDDGYRWRKYGQKVVKGNPNPRSYYKCTNAGCTVRKHVERASNDLKSVITTYEGKHNHDVPAARSSNHVSSCPSSTAPTQVAQPRIHRPEPSQINDGMVNFDGPASVGAFSLPGRELGTSPGFSFGMRQPGLAGLAMAGLGPGLRKLPASPLHPYLGQQRHMNETGLIMPKGEPKNEPVSETGLNLSDSSSVYHQIMGRLPLGSQM |
| AktWRKY39 | ATGAGAGAAGACAATGATGTAAAAGAAAGCAAGCCACAAAGAAACATAGGCATAAAAGAAATCCATCATAAGAGATCAAGAATAGGAGGTTCTTCCTCTGAAAAAGCAGACCCAAAGCCTTCAAAAGAAACCAATATGGAAAATCAAGCCTCCAAAAGAAGAAAAGTGTTTCAGAAGACAGTGGTGACAGTGAGGATTGAAACAAGCAGTGGTCGACAGAAGAATGAAGGGCCACCTTCTGATATCTGGTCTTGGAGGAAATATGGACAAAAACCCATCAAAGGATCCCCTTATCCAAGGGGCTATTATAGATGTAGCACATCAAAAGGTTGTTCAGCAAAAAAGCAAGTTGAGAGATGCAGAACAGATGCTTCAGTGCTCATCATCACTTACTCCTCAAGTCATAACCATCAAGACCCTGATCACATCCATCCCAACAGTCTAAATCAAGAACAAGACTTTGAAACCCAACCCACTGATGAACTGCCCACCACCCCAAAACAACAACAACAACAACAGCAATCTATCATCACTAGTGCAGAAGAAAAACTCACTGAAGATCATCTACACTACTTCCAATCACCAATCAAACCTTTAAACATAACTCTAGAGGAAACCCATGACTCATTAAACCTCCTGTTTGATGAAGAACCTATCTCTTATCCCACTCTCATGACCTTCTCTACTCCCAAATCTGAAGAAAATGACTTCTTTGATGAACTTGAGGAGCTACCCATCTCTTCATCTTTTACAAGCTTTATAAGCAGCAATTTCTTTGATGAAACAAAAGATCAGGATTCCTTCAGCCAACCCCAAATTTTTGGGATAAGGCTTTGA |
|  | MREDNDVKESKPQRNIGIKEIHHKRSRIGGSSSEKADPKPSKETNMENQASKRRKVFQKTVVTVRIETSSGRQKNEGPPSDIWSWRKYGQKPIKGSPYPRGYYRCSTSKGCSAKKQVERCRTDASVLIITYSSSHNHQDPDHIHPNSLNQEQDFETQPTDELPTTPKQQQQQQQSIITSAEEKLTEDHLHYFQSPIKPLNITLEETHDSLNLLFDEEPISYPTLMTFSTPKSEENDFFDELEELPISSSFTSFISSNFFDETKDQDSFSQPQIFGIRL |
| AktWRKY40 | ATGGACTTGACGACTTCAGTGGACACTTCCCTCAGCCTCGACCTCAATATTAATCCTTCATGGTGTCACGAAGATGTTCGGGCTGGTGTTTTAGTAGAGAAGCTCGATAAGATGAATGCAGAAAACAAGCGACTCACCGAGATGTTGGCCATTACGTGCCAAAATTATAACATTTTGAAGAGTCATCTCAAAGAATTGATGAGCAATAAGCCCGAAAAAATGAGGACTACATCAAGGAAAAGGAAGGCCGGAAGTGATGCTTACGTCAATAACAATGGAATCAATGTTAGTGTGGATAGTAGCAGCTCTAATGATGAAGATTTAAGTAAGAGACTAAGAGAAGACGTCAAGACGAAGATCTCGATGGCTTATGTAAGAACCGATCCATCTGATACAAGTCTTGTAGTGAAGGATGGATATCAATGGAGGAAATATGGGCAAAAGATTACCAAAGACAACCCATGTCCTAGAGCTTATTTCAAGTGTTCCTTTGCCCCAAGTTGCCCTGTCAAGAAGAAGGTGCAAAGAAACGTTGAAGATCGATCGATCCTTGAAGCAACATACGAGGGTGAGCACAATCACCCAAGATCTTCTCGAGTTGAAACATCAATAAATTTAATTCATGGTGTAAATGTTGATTCGGTTCCTTCTTTAGCTTCTAGTAGTCCTTCAGACCTCTCGAAGAAACTTGACCAGACCCAACCTGGATTACATAATGATGCCGAAAAAACTAGTAGGGAAATTGAATCACCAATGTTCCAACAATTTTTCGCCGAACAGATGGCTTCTTCCTTGTCAAAGGATTCTGGTTTCAAAGAAGCACTAGCATTGGCCATATCAGGAAGAATTCTTCAACACTCACCTACAAAAAAATGGTGA |
|  | MDLTTSVDTSLSLDLNINPSWCHEDVRAGVLVEKLDKMNAENKRLTEMLAITCQNYNILKSHLKELMSNKPEKMRTTSRKRKAGSDAYVNNNGINVSVDSSSSNDEDLSKRLREDVKTKISMAYVRTDPSDTSLVVKDGYQWRKYGQKITKDNPCPRAYFKCSFAPSCPVKKKVQRNVEDRSILEATYEGEHNHPRSSRVETSINLIHGVNVDSVPSLASSSPSDLSKKLDQTQPGLHNDAEKTSREIESPMFQQFFAEQMASSLSKDSGFKEALALAISGRILQHSPTKKW |
| AktWRKY41 | ATGGAGGTGAACGAGGATTGGGAGCGAAAGCCATTGATAAATGAGTTAATTCAAGGGAGACAGCTAGTGCAACAGCTTCGGATCCATCTCAACCCTTCTTCTCCGATTGGAGAATTACTAATCGAAAAGATCTTATCTTCATTCGATAACACCCTGTCGATGATTCATAAGAACGGACTCGATGAATTGTTTAAGCCAACAGGACCCACAATTGGCATGTTGGAGTCCCCACTTTCAAGTCCCCAAAGTGATGATACCGATCGCGCTGACACATCCAAGAAAAGGAAGACGCCATCGCAATGGACTGAACAAATACAAGTTTGCTCGGGGCCAGGGCTTGAAGGACCTCTTGAAGATGGATTTAGTTGGAGGAAATATGGGCAGAAAGACATTCTTGGAGCCAAGTATCCAAGAGGCTATTATCGATGCACGTATCGTAATGTTCAAGGGTGTTTGGCTACAAAGCAAGTACAAAGATCCGAGAACAACCCATCGATGTTCGATGTGACTTATCGAGGAAGACACACCTGCATCCAAACATTCCCCCAAACTCGGCCAACCCAAAACCCAAATCAAGAAGATCAAAAACAAAAACAAAAACAAAAACAAGAACAAGAACAAAAGCAAAAACAAACACAAGAAACACTCTTGAATTTTAAAACTGGCCTCGAATTCAGAAAATTCGAAAACTTTGACACCCTAGAGCCAAATTCCTTCACTTTTCCTTCTTCATCAATTGAAGGTGTGAAGATTGATAAAAATCACATATTTTCATCATCGATGCTCGATAACAATTTTATGGGTAGTTTTTCTCCACCATTTATATCTCCAGCTACATCGGAATCACAATGCAAGATGAATGGTTTTGGAGGTAGATTGAATTTGCAAACTTCAGAGTCTGATCATACTGAGATAATATCAGCCACTACTTCGTCCTCGAATTCTCCCATTGTGGACTTGGGTTTCACGCTTGATTCATTCGCATTTGACCCGAATTTCCAATTTTGCAATTCAGACTTCTTCAATTGA |
|  | MEVNEDWERKPLINELIQGRQLVQQLRIHLNPSSPIGELLIEKILSSFDNTLSMIHKNGLDELFKPTGPTIGMLESPLSSPQSDDTDRADTSKKRKTPSQWTEQIQVCSGPGLEGPLEDGFSWRKYGQKDILGAKYPRGYYRCTYRNVQGCLATKQVQRSENNPSMFDVTYRGRHTCIQTFPQTRPTQNPNQEDQKQKQKQKQEQEQKQKQTQETLLNFKTGLEFRKFENFDTLEPNSFTFPSSSIEGVKIDKNHIFSSSMLDNNFMGSFSPPFISPATSESQCKMNGFGGRLNLQTSESDHTEIISATTSSSNSPIVDLGFTLDSFAFDPNFQFCNSDFFN |
| AktWRKY44 | ATGGAAGTCCAAGAGACAGATAGGATGGTTATAGCTAAACCTGTTGCTTCAAGGCCTTCACTTTCAAACTTCAGATCATTTTCTGCGCTCCTTTCGGGTGCCATCAGTGCCACACCCCCGTGTGCATTTTCTGAAACAACTGTTTCTGCCGTTAGACCCAAGACAGTGAGATTCAAGCCGACAGTCAGTCGGTCTTCGATTGAAGCCATTTCATCGCTGGGTGAGATATCTGGAACAGAAGTTTGTTATTCGTCGGACAAGGTTGTGGTACCTGAAAGCAAGTCTACTGTGGTATATAAACCATTGGCAAAGCTTGTATCAAGTAAAACTGTTCATTTCTTGGCAAATCTGGGAAACTTCGATATCACTAATCAACAAACCGCCGCAACACGGGTGCAGCCCCAGCTTACTTCAAATCTTCATCGAAAACTTCCACCACAAATGAAAACAAACCAAACAATTGAGTCCTTGAAGATGGCATCACAGAACATGGAAGAGAATCAGAGAGCACTATCATCCACAACTAGTGGGGATCAACCTTCTTACGATGGATATAATTGGAGAAAGTATGGGCAAAAGCAAGTGAAAGGAAGCGAGTACCCACGAAGTTACTACAAGTGCACGTATCCAAATTGTGCTGTGAAGAAGAAGGTTGAGAGATCACTTGATGGACAGATAGCTGAAATTGTCTACAAGGGTGAGCACAATCATTCGAAACCTCAGCTACTTGTCTACAAGGGTGAGCATAATCATTCGAAACCTCAGCTTCTCAAGCGACAGTGTTCAGGATCACAAGAGCAAGGATTTGTGTCTGATGTGATCAGTCAAGAATTGGGGAATCCATCAGGGAGTAACCGAGTTAATGAGAGGAAGGAAGGATTGAAAGGTAGAAAAGAGAATAAGAATGAAGTAGGATTGTCGGTAAATCCTTCCTATACGGGCAAAGCTCAAATCCCCAACTATCCTATGAGGACTTGTAATGGTGTAGGAACTCCTGATAATTCGGGTGGTCTTAGTGGAGATTGTGAGGAAGCAAGCAGGGGCGTCGATGCAGATGATGATGAGCCCAAAATTAAGCGAAGGAAGAACGGTCACCAATCCAATGAAGTAGGTCTACTGGGGCAAGATGTACGAGGGCCTCATATTGTGGTGCAAAATTCTACAGATTCTGAGATTTTGGGGGATGGATTTCGTTGGCGAAAGTATGGGCAGAAGGTCGTGAAGGGAAATCCATACCCTAGAAGTTACTACAGATGCACCAATCTAAAGTGCAACGTGCGCAAGCATGTAGAAAGAGCATTAGATGATCCAAGAGCTTTTATCTCAACATACGAGGGAAAGCATAATCACGAAATGCCCACAAACCTCAATCTTGGTGCTTCTGACCCAGATTCAGTAACTCCTACTAACAAAGCGAAGCGATGA |
|  | MEVQETDRMVIAKPVASRPSLSNFRSFSALLSGAISATPPCAFSETTVSAVRPKTVRFKPTVSRSSIEAISSLGEISGTEVCYSSDKVVVPESKSTVVYKPLAKLVSSKTVHFLANLGNFDITNQQTAATRVQPQLTSNLHRKLPPQMKTNQTIESLKMASQNMEENQRALSSTTSGDQPSYDGYNWRKYGQKQVKGSEYPRSYYKCTYPNCAVKKKVERSLDGQIAEIVYKGEHNHSKPQLLVYKGEHNHSKPQLLKRQCSGSQEQGFVSDVISQELGNPSGSNRVNERKEGLKGRKENKNEVGLSVNPSYTGKAQIPNYPMRTCNGVGTPDNSGGLSGDCEEASRGVDADDDEPKIKRRKNGHQSNEVGLLGQDVRGPHIVVQNSTDSEILGDGFRWRKYGQKVVKGNPYPRSYYRCTNLKCNVRKHVERALDDPRAFISTYEGKHNHEMPTNLNLGASDPDSVTPTNKAKR |
| AktWRKY46 | ATGCAGAACACAATATATTTAGATAAAAAACATTTGATGATCAATGTGTTGACCCAAGGAAAGGAACATTTGAATCAGCTTCGAATCCATCTCGGTCCCTCATCTCGGACAGGAGAATTTCTAATGGAAAACATTCTACCTTCCTTCAACATGACTCTTTCGATTCTGAATTCAAGTGTTTCAGAAACACAGATTCAACCAATAGGACCCATCTCTAGTATATTGGGTTCTCCACATGCGATCAATGGGGATCCACAAAGAGACGATTCGAATCGAAGAGATAGGGAAAAGATGCCTTCATGGACTGAACGAGTGCGTGTTCACGCTGGGACTGAACTTAAAGATGCTTTGGTTGATGGTTACAGTTGGGAGAAATATGGACAACAAAGGGTTCTTGGAGCTAAGTACCCAAGATGCTATTATCGATGCGCTCATCATTACCCTCAAAATTGTAGAGCTACAAAGCATGTTCAGCATTCAAAAGATGACCCATCAATCGTTGAAGTCACTTATCAAGGAGAACACACTTGCATCCCAGCACTCTGA |
|  | MQNTIYLDKKHLMINVLTQGKEHLNQLRIHLGPSSRTGEFLMENILPSFNMTLSILNSSVSETQIQPIGPISSILGSPHAINGDPQRDDSNRRDREKMPSWTERVRVHAGTELKDALVDGYSWEKYGQQRVLGAKYPRCYYRCAHHYPQNCRATKHVQHSKDDPSIVEVTYQGEHTCIPAL |
| AktWRKY47-1 | ATGGAGAGAAGCCGAGAACTGACACTCTTACACACCGGTCAGAATTCCGGTGCTTCTGATCACCAAATTGAGAATTCAGTTGATCGGAAGGTTGTTATGCCGATGGAAGAGGTCGATTTCTTCTCCAATATTCATCATCATCGTCGACAGGTCGAAGTTCTTCGATCAAACAGTCAAATCCAAGATCAAGAGAGGAAAGATAAAATCGAACAGCCCCGTGTAAGCACTGGATTGCATCTTCGTACTCTCAATACCGATATTGAAGGACCCACAATTGAAGAGAAACAGATGACCCAATTGAGAACAGTTAAATTGGAACTCGATCGATCGAAAGATGAGAATAAAAAATTGAGGAGCATGTTGGATCAGGTAACCAAAAATTACAGTGCCCTTCAGGCTCATATACTTCTGGCCATGCAACAACAAACACGGGATAATCGTCAAGAACAGAAGGACCAAAGAAATGGCATGTCAAGCTCAGCATTGTCTGCCCATCAGTTCATGGACCCCAGTCCCTCCGGTGCATTAGATATCAACGAGCTTTCACACTCCAACGATGAGACACAAGATCCATCAACTTCTCCTGCTAATTGCATTGAAGTTATGTCGAAAGAATCAGATCATGACATGACTCAGATTGCAAGGAAGAGGTCTTGTACCGAAGATAACGCTGTTCAAAATTCCCGGAATTCGGGGCCTAACAAGAGCCCAAAGCTAGCACAAGCGAAATGCATCGAACAAGTTCCTGAGGTCCCATGTCGGAAGGCAAGAGTTTCTGTAAGGGCACGATCGGATGCACCACTGATAAGTGATGGTTGCCAATGGCGAAAATATGGTCAAAAGATGGCGAAGGGTAATCCTTGTCCACGTGCTTACTATCGTTGCACCATGGCTGTTGGATGTCCTGTTCGTAAGCAGGTCCAGAGATGTGCAGATGACAAGACCATTCTCATAACAACCTACGAAGGAAACCACAACCACCCTCTCCCTCCTGCAGCCACAGCCATGGCTAACACAACGTCAGCTGCTGCAACGATGTTACTCTCGGGTTCAACCATGAGCAAGGAAACCCTCGCAAATTCTTGCTTCTTCCCATCTTTTCCCTATACATCCACCATGGCTACCCTATCTGCCTCCGCCCCATTTCCTACTATCACTCTTGACTTGACACAGACCCCCTCAAACAACATGCCATTCCAACGTGCTTCACTTCCATCCACACCCTTCCCGTTGCCATTGAATGGTTTGCCATGGCAGTTAGGGCAGCAACCCATGTACGTTCCCCCCATGCCCACGGTGCAACTAGCACAGCGTCACCCATCCATGGTTGAGACCATCACTGCAGCGATAGCCACCGACCCTAACCTAACTGCAGCCGTGGCTTCAGCTATATCATCAATTATGAGTGCACCTCGAAGCGAAGACGGCAATGTTAATAATTCTGGTGTGCCTGTATTGTCACAATTTTCTCAGCCCTGCACCACTTTCCCTACCAACTAG |
|  | MERSRELTLLHTGQNSGASDHQIENSVDRKVVMPMEEVDFFSNIHHHRRQVEVLRSNSQIQDQERKDKIEQPRVSTGLHLRTLNTDIEGPTIEEKQMTQLRTVKLELDRSKDENKKLRSMLDQVTKNYSALQAHILLAMQQQTRDNRQEQKDQRNGMSSSALSAHQFMDPSPSGALDINELSHSNDETQDPSTSPANCIEVMSKESDHDMTQIARKRSCTEDNAVQNSRNSGPNKSPKLAQAKCIEQVPEVPCRKARVSVRARSDAPLISDGCQWRKYGQKMAKGNPCPRAYYRCTMAVGCPVRKQVQRCADDKTILITTYEGNHNHPLPPAATAMANTTSAAATMLLSGSTMSKETLANSCFFPSFPYTSTMATLSASAPFPTITLDLTQTPSNNMPFQRASLPSTPFPLPLNGLPWQLGQQPMYVPPMPTVQLAQRHPSMVETITAAIATDPNLTAAVASAISSIMSAPRSEDGNVNNSGVPVLSQFSQPCTTFPTN |
| AktWRKY47-2 | ATGGAAAGACGCCGGGAAATGACACTCTTACACACAGTCGAAAATCCTGATGTCTCTGATCATGTGATTGATAATTCATCTGATAGGAAGCATACGATGAAGGAGATGGATTTCTTCTCCGATAATCGTGGATACTCGGATGATATTCGTTACCAGGACCATAGGAGAATGGATTCATCAACAAAAAGCGAGTCTGGTGTAAATACCGGATTAAATCTTCTAACTCTCAATTCTGGAATCGAACAATCAACAATTGAAGAGAAACCGAAGGCACAATTGAGTATGCTTCGGGTTGAACTAGATCGAATGAACAACGAAAATCGGAGGCTTAGAAGCTTATTGGATCAGATAACCAAGAACTACTGTGCCCTCAAGAGCCAGCTAATGCTAACAATCCAACAACAGGCATGCAAGAACCGACAGGAGCAGAAGGATGAGAGCAATGGTTTATCTGCCCAACAGTTCATGGACCCCGGTCCATCTGATGGCCTAGATATCAATGAACCTTCAAACGATAATCTCGAGCCTCAAGAACAATCGGCTTCTCCAATTAACAATGTCAAAATGATGTCCACAGATCATGACATGAATCAGATTTCCAGGAAACGACCATCAATCGAAAATGGTTCCGATCATATGACCCCGAGTTGGGGGCATCACAAGAGTCCTAAGCCCACACAACCTGATAGTATGGAAGAAGTTCCTGAGGTCCCCTTTCGAAAAGCGAGAGTGTCTGTACGAGCACGATCTGATGCGCCTATGATAAGCGATGGTTGCCAATGGAGAAAGTATGGTCAAAAGATGGCGAAAGGCAACCCTTGTCCGCGTGCTTACTACCGTTGCACCATGTCCGTTGGATGCCCAGTTCGTAAGCAGGTGCAAAGATGTGCTGACGACAAGACCATTCTCATGACAACTTATGAAGGAAACCACAACCACCCTCTCCCTCCTGCAGCCACAGCTATGGCTAACACAACGTTGGCAGCATCCTCCATGCTCCTCTCAGGCTCGACCACTAGCAAGGAAACCCTAATGAATTCTGGCTTCTTCCCATCTTTTCCCTATGCATCCACCATGGCCACCCTATCTCCTTCCGCATCATTTCCCACCATCACCCTGGACCTGACCCAGACTCCCAACCCCATGCAATTCCAACGTCCACCACCTCCCTCAACGCCCTTTCCACTACCCTTCAATGATTGTCAAAAGCTGTTGGGGCAACGGCCAATGTACATGCCCCCTAAGATGTCTGTGATTCCAGGGATGCAGCTCCGCCAACGACCCCCGTCCATGGTTGAAAGCGTGACTGAAGCAATTGCAACCGATCCAAATTTCACGGCGGCCCTAGCTGCAGCCATCTCATCAATTATAGGTGCACCGCGGAGCAATGATGGAACCAACAACAACAACAATAATGGTGCTACAGTACCTGGGTCACCACAACTTCCACAGTCCTGCACCACTTTGACTACCAACTAG |
|  | MERRREMTLLHTVENPDVSDHVIDNSSDRKHTMKEMDFFSDNRGYSDDIRYQDHRRMDSSTKSESGVNTGLNLLTLNSGIEQSTIEEKPKAQLSMLRVELDRMNNENRRLRSLLDQITKNYCALKSQLMLTIQQQACKNRQEQKDESNGLSAQQFMDPGPSDGLDINEPSNDNLEPQEQSASPINNVKMMSTDHDMNQISRKRPSIENGSDHMTPSWGHHKSPKPTQPDSMEEVPEVPFRKARVSVRARSDAPMISDGCQWRKYGQKMAKGNPCPRAYYRCTMSVGCPVRKQVQRCADDKTILMTTYEGNHNHPLPPAATAMANTTLAASSMLLSGSTTSKETLMNSGFFPSFPYASTMATLSPSASFPTITLDLTQTPNPMQFQRPPPPSTPFPLPFNDCQKLLGQRPMYMPPKMSVIPGMQLRQRPPSMVESVTEAIATDPNFTAALAAAISSIIGAPRSNDGTNNNNNNGATVPGSPQLPQSCTTLTTN |
| AktWRKY49 | ATGGAGAAACTAAATGCTAATTGGTCGGATGGGTGGTCGGACGATGATCTTGTGAACGAGCTTCTCGACAACGAATCACCTTTCTTTGTATTGCCCTTGCCCGAGATCATTGAATGCGAACCAAGTCCTTTATCAAAACCCATATCAAACCATCTTGTCTCCAAAGTCTACTCTGGACCAACAATTCAAGATATCGAGAGTGTGTTATCGGTAACAAACCATATCAATCGATCCAATGATCAAAGTATTCCGGTGGGAGGCGGGATTTCAATTATAGAGAAAGGATTAAGTAAGATTGATAATAAGTACACTCTTAAAATCAAGAGTTGTGGGAATAATGGAATGGGAGATGATGGTTACAAATGGAGGAAATATGGGCAGAAATCAATCAAAAATAGCCCCTACCCAAGGAGTTATTATAGGTGCACCAATCCAAGGTGCAGTGCAAAGAAGCAAGTGGAGCGATGTAGCAAGGACCCAGACACACTCATCATCACCTATGAAGGGCTCCATCTCCACTTCACCTATCCTCATTTCCTTCTAGCCGAACCACAACATGCCTTACCACCCAACAAAAAGTCCAAGAAAACAACTACACAAACACCAGAATCTGAAGAAGCCCAAGAAATTCCTCCACTACTACTGGACCCTATTCCATTAAGCCCACCAGCTATTTTTAGTCCACAACAAGATATGTACAAAGGGATGGGCCCGCAAGGGTTGCTTGAAGATGTTGTGCCTTTAATAATTCGAAATCCATCAAACACCACCACCACCACCAGTTTGTCAAGCTCTTCTTCTTCTTCCTATCCATCTTCTCCTCTTATTTCTTCTTCTTCCTCCCTAAGTTGGTCTCCTAACTTTTCTTCTTTTGATGTTAGTGTCATTTCTAGTACAATGTGA |
|  | MEKLNANWSDGWSDDDLVNELLDNESPFFVLPLPEIIECEPSPLSKPISNHLVSKVYSGPTIQDIESVLSVTNHINRSNDQSIPVGGGISIIEKGLSKIDNKYTLKIKSCGNNGMGDDGYKWRKYGQKSIKNSPYPRSYYRCTNPRCSAKKQVERCSKDPDTLIITYEGLHLHFTYPHFLLAEPQHALPPNKKSKKTTTQTPESEEAQEIPPLLLDPIPLSPPAIFSPQQDMYKGMGPQGLLEDVVPLIIRNPSNTTTTTSLSSSSSSSYPSSPLISSSSSLSWSPNFSSFDVSVISSTM |
| AktWRKY50 | ATGGCCGATTCTGCTTTCATGGTAAGCGATTTTCTCAAAAACCCTAACCCTAATTACACTCATCTAATGGATCAAACGGATTTAATGGAGTTTGAAGTCTCCGATTATCTCTTATTCGAATCTGGCTCCGAAAACGACTCCACTTCGACTCACATCGGATGTGATGAATTTCCCATTAATCCTATTTTGCAAGAAAAGATCGAAGATGTCGTCGAATACAAGGATGACACAACACCGAGAAGTAGTAACATGCAAGGTAGAAAATGTAAAAGTGGGATGAAGAAATTAAAGATGGAAGTGGGGTTTAGGGTCGCTTTTCGAACAAAGTCGGAGCTAGAAATCATGGATGATGGATTTAAATGGCGAAAATATGGGAAGAAATCCGTGAAGGACAGCCCAAATCCGAGGAATTACTATCGTTGCTCGAGTGGTGGGTGCCAAGTGAAAAAAAGAGTGGAAAGAGACCGAGAAGACTCAAGTTATGTGATAACGACGTACGAGGGGGTGCACAACCATGAAAGCCCTTGTGTTGTGTACTACAACCATAAGCCTTTAATGGTTCCCACAGGATGGACTCTCCAAGCTTCTCACTCGTGA |
|  | MADSAFMVSDFLKNPNPNYTHLMDQTDLMEFEVSDYLLFESGSENDSTSTHIGCDEFPINPILQEKIEDVVEYKDDTTPRSSNMQGRKCKSGMKKLKMEVGFRVAFRTKSELEIMDDGFKWRKYGKKSVKDSPNPRNYYRCSSGGCQVKKRVERDREDSSYVITTYEGVHNHESPCVVYYNHKPLMVPTGWTLQASHS |
| AktWRKY51 | ATGGAAAGTGGTACAAACTTCGAATTTTCGGATTTTCTAGAGATTGAAGAATGGTTTGAGGAAGATCAAACTCCCATGGATTTTGAATCTTTCAAAAATCCATTTTCTTCGACGATGAAAACCGATGATCATGGTGATAGTAGTAGTCATGAAGGTGGTACCAGCAAGAGAAGTGGAAATGGTGGTGAGAAGAATAAGGAGGTGAAATCTAAAGTTGCATTTAGAACAAAATCAGAGCATGAGATCTTAGATGATGGGTTCAGGTGGAGGAAGTATGGGAAGAAAATGGTGAAGAATAGCCCAAATCCAAGGAATTATTACAAGTGCTTAGTTGATGGGTGCCCTGTGAAGAAGAGAGTGGAAAGAGATGGGGATGATCCAAAGTATGTGATAACAACATATGAAGGAATCCATAACCATGAGTGCCCCTCCCACTAA |
|  | MESGTNFEFSDFLEIEEWFEEDQTPMDFESFKNPFSSTMKTDDHGDSSSHEGGTSKRSGNGGEKNKEVKSKVAFRTKSEHEILDDGFRWRKYGKKMVKNSPNPRNYYKCLVDGCPVKKRVERDGDDPKYVITTYEGIHNHECPSH |
| AktWRKY53 | ATGGAGAACACAAGAGATTTAGATCAAAAACCTTTGATGATCAGTGAGTTAACCCGTGGAAAGGAACTCTTGAATCAGCTTCGAATCTATCTCGATCCCTCTTCTTCGACAGGAGAATTTCTAATAGAAAAGATTCTATCTTCCGTTAACAAGGCTCTTCTGATTCTGAATTCAAATGTTTCAGAAACAGAGATTCAACCAACAGGACCCATCTCTGGTATATTGGATTCTCCACGTTCAATCAATGGGAGTCCACAAAGCGACGATTCGAATCGAAGAGATATGTATAAGAAGAGGAAAACGTTGCCTCGATGGACTGAACAAGTGCGTGTTTGCTCTGGGACTGGACTTGAAGGTCCTCTTGAAGATGGTTACAGTTGGAGGAAATATGGACAAAAAGACATCCTTGGAGCCAAGTACCCAAGAGGCTATTATCGATGCACTCATCGTAATGCTCAAGATTGTAATGCTACAAAGCAAGTCCAACGATCCGAAGATGACCCATCAATCTTTGAAGTCACTTATCGAGGAAAACACACTTGCATTCCTGCATCCCGTCTAGCTCAGGGTTTGCCACCACCAGATAAACAAAAGCAAAATTCAAATCAACAAGAACAACAAAGGCAAAAGGAATCACAAGAGATACTCTTGAACCTTAAATCGGGCCTTAAAGTCAAAACCGAAGGCTTGGATACCCGAGAATTAACATCTCCTTCTTTCTCATTCCCTTCAACATCAATCGAGTGCATAAAAACAGAAAATGAAATATTTTCATCATTGACACTCGATAACAATCTCATTGGTAACTTTTCTTCTCCCTTCATATCCCCAACGACGTCCGAATCAAACTACTTCTCACCATGCCGGATATTAGGTACACCAAATTTGCAAAGCTTGGACTCTGATCTCAATGAGATCATCTCAGCTGCTACTTCATCCTCGAATTCTCCTACTTTGGACTTGGATTTCTCACTTGATCCAATTGAATTCGACCCGAACTTCCCATTCGACAGTCTAAGCAtgttcaattaa |
|  | MENTRDLDQKPLMISELTRGKELLNQLRIYLDPSSSTGEFLIEKILSSVNKALLILNSNVSETEIQPTGPISGILDSPRSINGSPQSDDSNRRDMYKKRKTLPRWTEQVRVCSGTGLEGPLEDGYSWRKYGQKDILGAKYPRGYYRCTHRNAQDCNATKQVQRSEDDPSIFEVTYRGKHTCIPASRLAQGLPPPDKQKQNSNQQEQQRQKESQEILLNLKSGLKVKTEGLDTRELTSPSFSFPSTSIECIKTENEIFSSLTLDNNLIGNFSSPFISPTTSESNYFSPCRILGTPNLQSLDSDLNEIISAATSSSNSPTLDLDFSLDPIEFDPNFPFDSLSMFN |
| AktWRKY54 | atggagtctccATGGCCGGAAAACTTATCAAATAATCAGAAAAAAATGAAGGAAAAGCTGATTGAAGGCGGGGAAGTAGCAATCGAACTTCAAAATCTCTTACAAAAGCCTCTCGGAGATCAAGGATTGGCGACGGTCGAAGACCTTACGGAGAAAATCCGGCTATTGTTCTCCGACACACTTTCCCTACTAAATTCTGATGATTCCGGCGAGATTTGTCTAAGTCCGGTAACTACTCTGGTAAGCTCACCTTTCTCCGATGACCGGAAAACGGACGATTCCGGTGAGAGTAGAAAAATTCTGGATTTTAAAGAAGATCGGCGAGGAGGATGTAAGAGAAGGAAGATTTGCCAGACACGTATGGAGGTTACACGTACACCTATCGACGACGGCTACTCATGGAGAAAATATGGGCAAAAACCAATCCTCAACGCCAAATTTCCAAGGAATTACTTTAGGTGTACACATAAGCATGATCAAGGTTGCCAAGCAATCAAACAAGTGCAGCGGACCGAAGATAATCCGCCACAGTACCGAACCACATACATGGGCCATCATACTTGCAAAGATACACTTTTAGTTCCAAAACTCATCTTAGATTCTACTCCAAGAGAATCTTTTGTCCTTGATTTTCAATCAAACATCACACCAAAACAACACCACCTCCCTTTCTCATCCTTCCACTCAATAAAGCAGGAATCTAAAGAAAAGATACCAAATGATCTGAACCAATTTCAATCATTATCGTCAGATCATCTTGATTGGCCTGATCTAACGATGTTGGATACATCTATGTCAAGAACAGTGTTGTCATCCACACCGAGGTCGGACTACCATGGGAATGTTACTTCTGATTTGTACACGTGTATGGCTAGTTCTCAAAGTTTGGATATGGATTTGATAGTGGGGTCTGTTAATTTTGATGATGATATGTTCCATTTCGATGAGCTATTTAATTAA |
|  | MESPWPENLSNNQKKMKEKLIEGGEVAIELQNLLQKPLGDQGLATVEDLTEKIRLLFSDTLSLLNSDDSGEICLSPVTTLVSSPFSDDRKTDDSGESRKILDFKEDRRGGCKRRKICQTRMEVTRTPIDDGYSWRKYGQKPILNAKFPRNYFRCTHKHDQGCQAIKQVQRTEDNPPQYRTTYMGHHTCKDTLLVPKLILDSTPRESFVLDFQSNITPKQHHLPFSSFHSIKQESKEKIPNDLNQFQSLSSDHLDWPDLTMLDTSMSRTVLSSTPRSDYHGNVTSDLYTCMASSQSLDMDLIVGSVNFDDDMFHFDELFN |
| AktWRKY57-1 | ATGGATGAAGATAAGAAAGCTGAACCAGGAGGAAGTACAGAAATTACAGGCGACTTCAGCTGGTTTGAGCCTGACTCCTTCTTCGATCGAGAAAGCAGTATTTTGAGCGAATTTGGATGGAATCAAGAATCTAGTAGTTTTCTTAAGTATGATCAGATCGATTCAGAGAAAGAGTTGGATTTGGCGGGAAGCTTTCCATTTCAGGAAAACTGCTTTTCCTCCTGTTTTAATCAGATAGTTCAGACGGGAACTGTCGATAAATCCGGCGATGCTTCAACGTCGAATCTGTCCGTATCTTCAAGTTCATGTGACGAGCAGCCGGAGAAATCGCCGAGATCTGACGGAAAACCGCCTGATAAACCAAGTCCTGGTAGAAAGAAAGGGCAAAAGCGAATCCGACAGCCACGGTTTGCATTTATGACTAAGAGCGATGTTGATCATCTAGAAGATGGTTATAAGTGGAGGAAATACGGACAGAAAGCCGTTAAAAATAGTCCATACCCTAGGAGCTATTACCGATGTACGAATAGCAAATGCACAGTAAAGAAAAGGGTTGAGCGCTCCTCTAAAGATCCCACCATTGTAATTACCACATACGAAGGCAAGCACTGTCACCACACAATCAGCTTGCCAGTACGAGGCGGTATCTTTTCTCATGGGCCGCCCACATTTGCCGGTCAGATGAACGTTTCAGCCCCACAGATATATTTTCCAGGGGGGCAATACCCTATAGGAGCAGGTCTACTTAATTTCAGGCAATCCCATCAACTCCAACACGAAGTCGGGTCAGCCCAAGTGCTGCCCAAGCCAGCTCCGCAGCTTCCAACTGATGAGGGATTGTTGGGTGATATTGTGCCCGGTGGAATGCGAAGAAGATGA |
|  | MDEDKKAEPGGSTEITGDFSWFEPDSFFDRESSILSEFGWNQESSSFLKYDQIDSEKELDLAGSFPFQENCFSSCFNQIVQTGTVDKSGDASTSNLSVSSSSCDEQPEKSPRSDGKPPDKPSPGRKKGQKRIRQPRFAFMTKSDVDHLEDGYKWRKYGQKAVKNSPYPRSYYRCTNSKCTVKKRVERSSKDPTIVITTYEGKHCHHTISLPVRGGIFSHGPPTFAGQMNVSAPQIYFPGGQYPIGAGLLNFRQSHQLQHEVGSAQVLPKPAPQLPTDEGLLGDIVPGGMRRR |
| AktWRKY57-2 | ATGGATGAGGAGAAGAATCTGAAACCAGAAACAGATACAGTTGGATACTCAAGTTGGATGAAGTCCACTAACTTCTTCTGCAGTGAAGGCGAAAGTAGTATTCTGAGAGAATTCGGATGGAATCTGGAGCCTGAGAGAGATCGGAGTCAAGAAACAAGCAGTTTTATGGATTTCGATCAGATCGATTTGGACCAGGGAACAGATTTGACGAGAAGCTTTCCATTGCCGGAAAACCACTCTTCTTCATGTTTTAACCAGGTGACTCCGACTGGATCGGTTGATAAATCCGGAGATGCTTCCACGTCGAATCCGTCGGTATCTTCAAGCTCGAGCGATGAGCAGCCGGAGAAATCGACGGGATCTGACCGAAAACCGCCGGACAAACCGAGTAAGGGTAGAAAGAAGGGGCAAAAGCGAACCCGCCAATCCCGGGTTGCATTCATGACTAAGAGCGATGTCGATCATCTTGAAGATGGCTATAGATGGCGGAAGTACGGACAGAAGGCCGTTAAAAATAGCCCATACCCAAGGAGCTATTATCGCTGTACAAACAGCAAATGCACAGTAAAGAAGAGGGTTGAACGCTCCTCTAAAGATCCTACTGTTGTAATCACCACATACGAAGGTCAACACTGTCACCACACTGTCAGCTTCCCCCGTGGTGGTATCCTTTCTCATGAGGCCGCCATCTTTGCTGGGCGTATGAACTCTATTAGTTCAATCCCACAGATGTATGTTCCAGGGGTGCAATACCCCATTGGAGCTCATCCATTTGATCATGTCAGGCGACAATCACATCATCTCCAACATGAAGTAGGGTCCACAGCACAAGTTCTTACTGATACAGCTCCTCAGCTTGCAACTGATTATGGATTGTTGGATGATATTGTGCCTGCTGGAATGCGCAGATGA |
|  | MDEEKNLKPETDTVGYSSWMKSTNFFCSEGESSILREFGWNLEPERDRSQETSSFMDFDQIDLDQGTDLTRSFPLPENHSSSCFNQVTPTGSVDKSGDASTSNPSVSSSSSDEQPEKSTGSDRKPPDKPSKGRKKGQKRTRQSRVAFMTKSDVDHLEDGYRWRKYGQKAVKNSPYPRSYYRCTNSKCTVKKRVERSSKDPTVVITTYEGQHCHHTVSFPRGGILSHEAAIFAGRMNSISSIPQMYVPGVQYPIGAHPFDHVRRQSHHLQHEVGSTAQVLTDTAPQLATDYGLLDDIVPAGMRR |
| AktWRKY58 | atggaggagaatgtgaaatctgagcttgaagatcctcatagaagagctcatgaatctgaaaccagagcTTACGAGCTTCAATTTGAGAGCTTTAATGGCGGTAACGGTTTGATTTCTAGTTCTAATAATGCTTTAATGGAAGAAGATCCAAAGAGGAACGTTTTTTCTGTGAAATCTATTTCTAGGGATTTCTCTGGTTCCGGATCGAATGCTGCGAGGTACAGATTGATGTCTCCAGCGAGCCTTCCGATCTCAAGATCGCCATGTTTGACGATTCCTCCTGGTTTGAGCCCGACAACGTTGCTTGATTCTCCTGTTCTTCTCTCAAATATGGCAGAACCTTCACCAACAACTGGTACCTTCCACAAGCCTCAAGTCATGCATGACAGTGTGGTTTCCGAGGCATTCTCTTCTCTGAGGGACACTTCAAATAGCAGTACTTATGATGAAAGAAGTTATGGCAACTTCGAGTTTAAACCTCACACTAATTCTAGCTCAGGTCCAGGCCTATCATCATTGGGACCTTTGGCTTCTACAGGTTTGAACCACCAGAAGTGTGAGTCATTTGTGCAAGTCCAAGGTCAATGCCAGTCTCAGACATTTCCATCCTCACCTTCGGTTAAAAATGAGAATATGGGAGCCTCCTCGCATGAATTGACTCTATCTGTAACCACTTCAAACCCACCAGTTTACATGGTGACATCAAGAGGTGGTGTAACAGCTGAAGTTGCTTCGAATGAGCAGTGGCCGAGACAGGGTTCTCACAGTGGGGTCCAAACAATGCAATCTGACCATAATGGGACTAGTCCTTTGATAGCAACTGAGAGATCATCAGAAGATGGATATAACTGGCGAAAATATGGGCAGAAACATGTTAAAGGATGTGAATTTCCTCGGAGCTATTATAAATGTACACATCCTAACTGCCAAGTGAAAAAGCAATTGGAACGCTCTTATGATGGCCAGATTACAGATATTATCTACAAGGGAAAGCATGATCATCCTAAACCTCAGCCTAGCCGTCGAATGGCAGTTGGAACAATTCTGTCTATCCAAGAAGAAGGATCTGATAGGTTTTCCCCTTTAAATGACACAGAAGACAAGTCATCAAATGAGCATGGCCAGACATATCAAATTGAACCAAATGGTACCCCTGACCTTTCTCCTGTTAATGAAAGTGGCAATAATGTAGAAGGCACAGGTGCACAATCAAACATTATTGTCAATGATGTTGATGATGACGATGATGATCCAGAATCTAAACGCAGGAAGAAAGACCTTGGTGGTGTAGATCTTACCCATATGGGTAAAGTGACCCGGGAACCACGCGTTGTTGTCCAAACTCTAAGTGAGGTTGATATACTGGATGATGGGTACCGCTGGCGCAAATATGGGCAGAAAGTGGTAAAAGGGAATCCTAATCCAAGGAGTTACTACAAATGCACAAATGCTGGATGCCCAGTTAGAAAACATGTGGAGAGGGCATCACATGATCCAAAAGCAGTTATAACCACATATGAGGGAAAGCATAACCATGATGTTCCAGCTGCAAGGAATAGTACCCATGATACAGCAAGGCCTGCAGTTTATAATACAACTTCGAATGGCATGTTAAGGACTAGATCTGAAGAAATTGACACAATTAGCCTTGATCTTGGTGTTGGGATTAGCTTGAATCCTGAAAATAGATCAATTGAGAAGCAGCAAACAATGGATGGAGAATCTGTCGAAAGTCAATCCCACATTGCCAATCCTAGTAGTAGTAAGGTGATACAAGGTGGAGCCCCAATTTTGGCATATACCAGTGGTTTGAATGATGGCACAGATCAGTATGGATCAAGAGAAGGCCGGGCTGAAAGCTTTAGCTTCGAGACAGGACCATTAAACCATTCCTCTAACCCATATCGGCAGAACATGGGAAGATTAATAATGGGTCCATAG |
|  | MEENVKSELEDPHRRAHESETRAYELQFESFNGGNGLISSSNNALMEEDPKRNVFSVKSISRDFSGSGSNAARYRLMSPASLPISRSPCLTIPPGLSPTTLLDSPVLLSNMAEPSPTTGTFHKPQVMHDSVVSEAFSSLRDTSNSSTYDERSYGNFEFKPHTNSSSGPGLSSLGPLASTGLNHQKCESFVQVQGQCQSQTFPSSPSVKNENMGASSHELTLSVTTSNPPVYMVTSRGGVTAEVASNEQWPRQGSHSGVQTMQSDHNGTSPLIATERSSEDGYNWRKYGQKHVKGCEFPRSYYKCTHPNCQVKKQLERSYDGQITDIIYKGKHDHPKPQPSRRMAVGTILSIQEEGSDRFSPLNDTEDKSSNEHGQTYQIEPNGTPDLSPVNESGNNVEGTGAQSNIIVNDVDDDDDDPESKRRKKDLGGVDLTHMGKVTREPRVVVQTLSEVDILDDGYRWRKYGQKVVKGNPNPRSYYKCTNAGCPVRKHVERASHDPKAVITTYEGKHNHDVPAARNSTHDTARPAVYNTTSNGMLRTRSEEIDTISLDLGVGISLNPENRSIEKQQTMDGESVESQSHIANPSSSKVIQGGAPILAYTSGLNDGTDQYGSREGRAESFSFETGPLNHSSNPYRQNMGRLIMGP |
| AktWRKY65 | ATGGAAGGTAGATTCAAGTTCAACAACCCTTTTGTAAGAGAGCAAGATGAAGATGTCAGAATGATGCCGGAGAACTGTGATTCTCCACCTAATTCCGGCATGATCAACGACAGAAAGAAGACTACCACATCGTCTCCGAAAAAAAGGCGCGCTGCACAGAAGAGAGTGGTATCCATACCGATCGGGGACGTTTCTCGTGTAAAGGGCGAGGGAGCTCCACCGTCTGATTCTTGGGCTTGGAGGAAGTATGGGCAGAAACCAATCAAGGGATCTCCTTATCCCAGAGGGTATTATCGATGTAGTAGTTCGAAAGGCTGTCCAGCAAGAAAACAAGTAGAGAGGAGCCGAGTGGACCCCACCATGCTCGTGATAACGTACGCCTGTGAGCACAATCACCCCTGGCCGGCCACCAAAAACTCTACTACAACTACCACCACCACTACCAATGACAACAAATCCCGGTCTGAAGAACCACCGGTTCTCTCGAACCAAAAAGAAACCGAACCGGACCACAAATTCGCCGATCTTGGTGACGAGTCTCTAATCACAACAGACGATTTCGGTTGGTTTTCCTACGTGGCATCCACAACTCCCACAATGCTCGAGAGCCCAATTTGCTCGGGAAACATCAACGAAGGTGCTGACGTGGCGATGATCTTTCCAATGGGAGAAGAAGACGAGTCGTTGTTCGCCGATTTGGGCGAGTTACCAGAATGTTCTGTGGTTTTTCAAAGAGGGTTTATCGAACAGGAGGAGCGGCGCACGCGGTGCAGTTTAAGGGAGGCTTCATGGTGCCAGAGCACAGGATGA |
|  | MEGRFKFNNPFVREQDEDVRMMPENCDSPPNSGMINDRKKTTTSSPKKRRAAQKRVVSIPIGDVSRVKGEGAPPSDSWAWRKYGQKPIKGSPYPRGYYRCSSSKGCPARKQVERSRVDPTMLVITYACEHNHPWPATKNSTTTTTTTTNDNKSRSEEPPVLSNQKETEPDHKFADLGDESLITTDDFGWFSYVASTTPTMLESPICSGNINEGADVAMIFPMGEEDESLFADLGELPECSVVFQRGFIEQEERRTRCSLREASWCQSTG |
| AktWRKY68 | ATGGAGAAGAAAGAAACAATGGAGACAGAGAATTTGATGGGTATGCCGACATTTTCAGATCAGATTTTTAGCAATTATTCTCTACAAGGCATCTTCGACATGTCGTTTGAAACCGAGAAAGCTTCTCTAGGTTTCATGGAGTTACTTGGTCAAGATTTTTCTCCCACAACTTTTGACAATTTTCTACAAAACCCATCTCCACCATTACCCCCACCACCACCTACAGAGACATCTGAGGTGTTGAATTTGCCTGCAACGCCGAACTCTTCATCGATTTCTTCTTCTTCGACTGAAGCACCTAATGAAGAACAAAAATCTAAGACAGTAGAGGACGAAGATCTGGAAAAAACTAAGAAAGAATTGAAACCTAAAAAGACGAAGCAAAAACGGCAGAGAGAGCCAAGATTTGCGTTCATGACAAAGAGTGAGATTGATCATCTGGAAGATGGGTACAGATGGAGAAAATACGGACAAAAAGCTGTGAAAAATAGCCCTTTTGCTAGGAGCTACTACCGTTGCACCAATGGCACATGTAGTGTGAAGAAGCGTGTGGAACGATCGTCCGAGGATTCGACGATCGTTGTAACAACCTATGAAGGCCAACACACTCATCCTAGCCCGATCATGCCACGAGGAAGCCATGCCGTAGTCCCACAAGTTTCAGGCAGCTTTGGGGCCGTGGACCCCGCCGCCGCTACCTTCGCTACTTCGATGCAAATGACACAACCTCAGTACCAACAACAACCCTATTTCCATAGCTTAATACCTCTTTCAAATTTTGGTTCGTCGACTTCACTTTCTTCATCTTTACCTACTTTTCTTCATGAGAGACGTTTTTGCACTCCATCAGCAACATCTTTGCTTAGAGATCATGGGCTTCTTCAAGATATCGTTCCGTCCGATATGCGTAAAGAGGAGTAG |
|  | MEKKETMETENLMGMPTFSDQIFSNYSLQGIFDMSFETEKASLGFMELLGQDFSPTTFDNFLQNPSPPLPPPPPTETSEVLNLPATPNSSSISSSSTEAPNEEQKSKTVEDEDLEKTKKELKPKKTKQKRQREPRFAFMTKSEIDHLEDGYRWRKYGQKAVKNSPFARSYYRCTNGTCSVKKRVERSSEDSTIVVTTYEGQHTHPSPIMPRGSHAVVPQVSGSFGAVDPAAATFATSMQMTQPQYQQQPYFHSLIPLSNFGSSTSLSSSLPTFLHERRFCTPSATSLLRDHGLLQDIVPSDMRKEE |
| AktWRKY70 | ATGGAGTCTCCTTGGCCTGAAAATTTCTCCTTTGATCGAAAGAAGGCAATTGAAGAGCTGGTTCGGGGCCAAGAATTCTTAACCCAACTTCGAAATCTCCTCCAGAAAAGGACTATGGTAGATCTTGAGTTGGTGTCAGGCCAAGACCTTGTAGTAAAGATATCTGATTCGTTCGCCAACGTTCTTTCGGTACTAAATTCCGGTGAGTCCAGTGATGTGTGTCATAGCCGGGAGAATAACCAACTTAGTTCACCTTGTTCTGATGACCGGAAAAGTGATGATTCCGGCAATAAAAAAAAGATTCCGGCTACAAAGGGAGGAGTGCATAAGAGAAGGAAGACATCATCCAATACATGGACCAGGGTCACTCCAACTCCAACCCCTATCAATGACGGGTACGCATGGCGAAAATACGGCCAGAAGGAGATCCTTAACGCCAAATTTCCAAGGAACTACTTTAGGTGCACTCACAAGCACGATCAAGGCTGCCAGGCAACCAAACAAATGCAAAGAACTGAAGAGGACCCACCCATGTACCTCACCACATACATGGGGCACCACACATGCAGGGCCACACTGAAAGCTCCCCAACTCATATTAGATACTTCTCCAAGAGACTCTTTTGTGTTCAACTTTGAATCAAACGTGTCAAATAAACAAGAGTTCCCCTTCTTTGCAATAAAACAGGAATCTAAAGATGAGATACTAATAAGTGATCTGACCCACAATTACTCATCAGAGTATCTTCTGCCAACATTAGAATCAAATGGACCCACAACAATGTTGGCTTCGACTTCAGGGTCAGATCACGGGGATGTAATGTCTGGTGTGTACGAATGTTCAACTAGTTCTCCGAGTTTGGATATGGATTTGCTTGTGGAATCTGTGGATTTCGATACTGTGTTCCGCTTCGACGATGACGAGTTCAAACCTCAGTTTTAG |
|  | NKREREREMMESPWPENFSFDRKKAIEELVRGQEFLTQLRNLLQKRTMVDLELVSGQDLVVKISDSFANVLSVLNSGESSDVCHSRENNQLSSPCSDDRKSDDSGNKKKIPATKGGVHKRRKTSSNTWTRVTPTPTPINDGYAWRKYGQKEILNAKFPRNYFRCTHKHDQGCQATKQMQRTEEDPPMYLTTYMGHHTCRATLKAPQLILDTSPRDSFVFNFESNVSNKQEFPFFAIKQESKDEILISDLTHNYSSEYLLPTLESNGPTTMLASTSGSDHGDVMSGVYECSTSSPSLDMDLLVESVDFDTVFRFDDDEFKPQF |
| AktWRKY74 | ATGGAAGGTTCATTTTCTGGAAAAATTGATGATTCAAACACTTCAAGAGAAATCAAGACAGAAGCCCAAACCCAAGTCTCTAAAAGAAGGAAAATGATTGAGAAGACTGTGGTGAGAGTGAAGATTGAAGAAAATGTTGGGAGACAAAAGATTGAAGGGCCACCTTCTGATTTCTGGTCTTGGAGGAAATATGGACAAAAACCCATCAAGGGATCTCCTTACCCAAGGGGATATTACAGGTGTAGCACATCAAAGGGTTGTTCAGCCAAAAAACAAGTAGAGAGATGCAGGACAGATGCTTCAATGCTCATCATTACGTACACCTCAAGCCATAACCATTCAAGCCTTAACATCCCCACCAATCTAACCCAACAACAACCACCATCACCACCACCACCACCACCAACCCAACCCACTGAGGAACAAAATCCCACCATCACAAGTGAGGAAGTACTCAGTGAAGATCACCCCCACTTCTTCCAATCCCCCATCAATTTCTCACAAGACATCAGCCATAACAAAGGAGAAAATCCTTTAACAGTAACACTAGATAAAACCCAGGACTCATCAAGTCTTCTCTTTGATGAAGAACCATTATCTTACCCCCATCTCATGAACTTTTCATCAACACCAAAATCAGAAGAAAATGATTTCTTTGATGAGCTTGAAGAGCTACCCACTTCTTCATCTTTCACAAGTTTTATGAGGAGCAATTTCTTTGATGAAAGGATCCTTGTTCTTCCTTCCTGA |
|  | MEGSFSGKIDDSNTSREIKTEAQTQVSKRRKMIEKTVVRVKIEENVGRQKIEGPPSDFWSWRKYGQKPIKGSPYPRGYYRCSTSKGCSAKKQVERCRTDASMLIITYTSSHNHSSLNIPTNLTQQQPPSPPPPPPTQPTEEQNPTITSEEVLSEDHPHFFQSPINFSQDISHNKGENPLTVTLDKTQDSSSLLFDEEPLSYPHLMNFSSTPKSEENDFFDELEELPTSSSFTSFMRSNFFDERILVLPS |
