# Supplementary material for: Characterization of the WRKY gene family in Akebia trifoliata and their response to Colletotrichum acutatum
Source: BMC Plant Biol. 2022 Mar 14;22:115. doi: 10.1186/s12870-022-03511-1 (PMC8919620; doi:10.1186/s12870-022-03511-1)
Supplement: Supplementary file 2 — Additional file 2. The number of WRKY genes belonging to each subgroup in different species. [file 12870_2022_3511_MOESM2_ESM.docx]

Additional file 2 The number of WRKY genes belonging to each subgroup in different species

| Gene | Phylogenetic group | | | | | | |
| --- | --- | --- | --- | --- | --- | --- | --- |
|  | I | IIa | IIb | IIc | IId | IIe | III |
| *AtWRKY* | 13 | 4 | 7 | 18 | 7 | 9 | 14 |
| *BdWRKY* | 16 | 3 | 6 | 21 | 6 | 10 | 22 |
| *OsWRKY* | 15 | 4 | 8 | 15 | 7 | 11 | 36 |
| *AktWRKY* | 12 | 2 | 3 | 10 | 4 | 4 | 7 |
| *AcWRKY* | 25 | 4 | 8 | 25 | 12 | 13 | 10 |
| *VvWRKY* | 12 | 3 | 8 | 15 | 7 | 6 | 6 |
| *SlWRKY* | 15 | 5 | 8 | 16 | 6 | 17 | 11 |
