# Supplementary material for: Characterization of the WRKY gene family in Akebia trifoliata and their response to Colletotrichum acutatum
Source: BMC Plant Biol. 2022 Mar 14;22:115. doi: 10.1186/s12870-022-03511-1 (PMC8919620; doi:10.1186/s12870-022-03511-1)
Supplement: Supplementary file 3 — Additional file 3. Expression data of the AktWRKY family genes after C. acutatum infection. [file 12870_2022_3511_MOESM3_ESM.docx]

Additional file 3 Expression data of the *AktWRKY* family genes after *C. acutatum* infection.

| **Gene name** | **C01** | | | **I02** | | | **H05** | | |
| --- | --- | --- | --- | --- | --- | --- | --- | --- | --- |
|  | fold-change | p-value | up/down | fold-change | p-value | up/down | fold-change | p-value | up/down |
| ***AktWRKY02*** | -1.77 | 0.02 | down | -1.06 | 0.05 | down | 0.24 | 0.05 |  |
| ***AktWRKY03*** | 5.16 | 0.01 | up | 2.56 | 0.01 | up | 4.68 | 0.00 | up |
| ***AktWRKY04*** | 2.56 | 0.02 | up | 3.34 | 0.01 | up | -1.46 | 0.03 | down |
| ***AktWRKY07*** | 1.67 | 0.00 | up | -1.97 | 0.00 | down | 6.48 | 0.00 | up |
| ***AktWRKY11*** | -4.25 | 0.00 | down | -6.50 | 0.07 |  | 5.26 | 0.00 | up |
| ***AktWRKY12*** | 3.41 | 0.00 | up | 5.84 | 0.00 | up | 1.65 | 0.03 | up |
| ***AktWRKY13*** | 3.67 | 0.00 | up | 5.71 | 0.00 | up | -1.20 | 0.05 | down |
| ***AktWRKY14*** | 3.16 | 0.01 | up | 5.52 | 0.00 | up | -3.75 | 0.02 | down |
| ***AktWRKY15*** | 2.43 | 0.01 | up | 5.57 | 0.00 | up | 2.62 | 0.14 |  |
| ***AktWRKY17*** | -1.88 | 0.03 | down | 0.81 | 0.46 |  | 1.19 | 0.02 | up |
| ***AktWRKY18*** | -4.63 | 0.00 | down | -2.92 | 0.02 | down | 3.56 | 0.01 | up |
| ***AktWRKY19*** | 2.69 | 0.01 | up | 2.89 | 0.02 | up | -5.02 | 0.04 | down |
| ***AktWRKY20*** | 1.20 | 0.00 | up | -3.25 | 0.01 | down | 5.03 | 0.00 | up |
| ***AktWRKY21*** | -3.30 | 0.00 | down | -3.96 | 0.00 | down | 1.69 | 0.01 | up |
| ***AktWRKY23*** | 1.84 | 0.03 | up | 3.50 | 0.00 | up | -3.98 | 0.01 | down |
| ***AktWRKY25*** | 0.97 | 0.28 |  | 3.82 | 0.03 | up | -0.52 | 0.29 |  |
| ***AktWRKY26*** | -4.29 | 0.01 | down | -3.95 | 0.01 | down | -1.52 | 0.01 | down |
| ***AktWRKY27*** | -1.43 | 0.01 | down | 2.42 | 0.01 | up | 0.88 | 0.24 |  |
| ***AktWRKY30*** | 0.89 | 0.16 |  | -2.00 | 0.02 | down | 2.66 | 0.02 | up |
| ***AktWRKY31*** | -4.66 | 0.01 | down | -3.96 | 0.03 | down | 3.69 | 0.12 |  |
| ***AktWRKY32*** | 1.42 | 0.01 | up | 3.59 | 0.01 | up | -0.18 | 0.62 |  |
| ***AktWRKY33*** | 3.26 | 0.00 | up | 1.70 | 0.00 | up | 7.27 | 0.00 | up |
| ***AktWRKY34*** | 0.91 | 0.09 |  | 2.32 | 0.01 | up | -0.72 | 0.02 |  |
| ***AktWRKY39*** | 1.99 | 0.05 | up | 3.12 | 0.00 | up | -0.56 | 0.03 |  |
| ***AktWRKY40*** | 5.66 | 0.00 | up | 6.71 | 0.00 | up | -3.12 | 0.04 | down |
| ***AktWRKY41*** | -0.51 | 0.54 |  | 2.57 | 0.11 |  | 6.12 | 0.00 | up |
| ***AktWRKY44*** | -1.56 | 0.07 |  | -1.01 | 0.17 |  | -3.94 | 0.01 | down |
| ***AktWRKY46*** | -2.70 | 0.00 | down | -2.27 | 0.53 |  | 0.13 | 0.88 |  |
| ***AktWRKY47-1*** | 6.48 | 0.02 | up | 4.91 | 0.00 | up | -5.16 | 0.00 | down |
| ***AktWRKY47-2*** | -3.67 | 0.00 | down | -4.49 | 0.02 | down | 1.85 | 0.01 | up |
| ***AktWRKY49*** | 0.19 | 0.63 |  | 3.38 | 0.07 |  | 3.93 | 0.09 |  |
| ***AktWRKY50*** | -5.98 | 0.00 | down | 0.57 | 0.32 |  | 2.34 | 0.02 | up |
| ***AktWRKY51*** | -6.16 | 0.00 | down | -1.52 | 0.22 |  | 1.83 | 0.02 | up |
| ***AktWRKY53*** | -0.87 | 0.01 |  | 2.10 | 0.01 | up | -0.24 | 0.19 |  |
| ***AktWRKY54*** | 0.70 | 0.15 |  | -2.85 | 0.01 | down | -0.44 | 0.26 |  |
| ***AktWRKY57-1*** | 4.07 | 0.01 | up | 5.28 | 0.00 | up | -1.47 | 0.04 | down |
| ***AktWRKY57-2*** | 2.17 | 0.07 |  | -0.17 | 0.73 |  | 0.73 | 0.42 |  |
| ***AktWRKY58*** | 0.19 | 0.31 |  | -3.44 | 0.01 | down | 2.63 | 0.39 |  |
| ***AktWRKY65*** | -6.66 | 0.01 | down | -2.27 | 0.46 |  | 4.50 | 0.01 | up |
| ***AktWRKY68*** | 5.20 | 0.01 | up | 3.87 | 0.05 | up | -3.19 | 0.01 | down |
| ***AktWRKY70*** | -3.51 | 0.01 | down | -6.72 | 0.00 | down | 1.73 | 0.10 |  |
| ***AktWRKY74*** | -5.10 | 0.01 | down | -6.10 | 0.00 | down | 2.52 | 0.02 | up |
