# Supplementary material for: Characterization of the WRKY gene family in Akebia trifoliata and their response to Colletotrichum acutatum
Source: BMC Plant Biol. 2022 Mar 14;22:115. doi: 10.1186/s12870-022-03511-1 (PMC8919620; doi:10.1186/s12870-022-03511-1)
Supplement: Supplementary file 4 — Additional file 4. The list of primer-sets of AktWRKY genes for qRT-PCR. [file 12870_2022_3511_MOESM4_ESM.docx]

Additional file 4 The list of primer-sets of *AktWRKY* genes for qRT-PCR.

| Gene name | Forward primer | Reverse primer |
| --- | --- | --- |
| *AktWRKY02* | CGGTCACCCTAAACCTCCAC | ATGATGTGGCCTCCAGGTTG |
| *AktWRKY03* | TGGAGAAAGGAGGGAGTGGA | TGGTGTTTCTTGGACCGCAA |
| *AktWRKY04* | GTACGGGCAGAAAGTCGTCA | GTTGTGGCTGCTGGTCTTTG |
| *AktWRKY07* | TGGGTGGTAATAAGTGCGGT | AGTACCCCCTTGGATGTGGA |
| *AktWRKY11* | TACCAAGTGCGGAGGAAGTG | GGCAACCCCTCATGCTACTG |
| *AktWRKY12* | GTCCCTCCCTCTCAGTCTTC | CACCATGAATTGTTGCCATC |
| *AktWRKY13* | CCCTCCCAACTGGACCTTTC | CTTCCACTTCTCCCCAAGCC |
| *AktWRKY14* | AGCCGAAAGGGAACAAACCA | CATTTCTGGCTGGTGCATCG |
| *AktWRKY15* | ATGGGCAAAAGGCAGTCAAG | AAGGTGCTAACATTCCAGCCA |
| *AktWRKY17* | GCCGTTGCCATTGTTCCAAG | TCATCTGGTGCTCGTTCCAC |
| *AktWRKY18* | CAAGTGCTCCTTTGCCCCA | GGAGGCAGAACAAGGAACCG |
| *AktWRKY19* | AGAAAGACATTGGCGGCGTA | ACTGGGCATCCAGCATTTGT |
| *AktWRKY20* | AAGCAACACCATCCGACCAT | GTGACATGCCCATCGTGAGA |
| *AktWRKY21* | CGTGGAGGAAGTATGGGCAG | CAGATTGCGAGAGCAGCCTA |
| *AktWRKY23* | CCTCCACCAACAACAACCAC | CGCAAATCTCGGTTCCCTCT |
| *AktWRKY25* | GAGTGCTGGATGCTCTGTGA | TTGAGGGTGGCTTGAGCATT |
| *AktWRKY26* | ACAACATACGAGGGGAAGCA | TGCCCGTCCGACATTTGTAT |
| *AktWRKY27* | CTGATATGTGGGCTTGGCGT | GGGGACTGGATGGTTGTGTT |
| *AktWRKY30* | CAAGTCCGTGTTTGCTCTCG | GGGTCATCTTCGGATCGTTGA |
| *AktWRKY31* | CTTCGGCTCCCTTTCCAACT | GTGAGAGCTGAAGGCCAGAG |
| *AktWRKY32* | ATGAAGGATGGTAGCGTGGC | CTTACGGACAGGGCATCCAG |
| *AktWRKY33* | TGGTAGTGGTAGCCATGCAG | CCGAAACTCCCTGGACTGTG |
| *AktWRKY34* | CCCTCACTTTGAAGCCACCA | TGGCTGTTTGGACGACAACT |
| *AktWRKY39* | AAACAAGCAGTGGTCGACAGA | GCACTGAAGCATCTGTTCTGC |
| *AktWRKY40* | AGATGTTCGGGCTGGTGTTT | CATCACTTCCGGCCTTCCTT |
| *AktWRKY41* | GAACGAGGATTGGGAGCGAA | ATTGTGGGTCCTGTTGGCTT |
| *AktWRKY44* | GCAGAAGGTCGTGAAGGGAA | CTGGGTCAGAAGCACCAAGA |
| *AktWRKY46* | TGTGTTGACCCAAGGAAAGGA | TGATCGCATGTGGAGAACCC |
| *AktWRKY47-1* | ACTTGACACAGACCCCCTCA | TTAGGTTAGGGTCGGTGGCT |
| *AktWRKY47-2* | AAACCACAACCACCCTCTCC | TGGTGGGAAATGATGCGGAA |
| *AktWRKY49* | AGTGGAGCGATGTAGCAAGG | TAGCTGGTGGGCTTAATGGAA |
| *AktWRKY50* | ACGACTCCACTTCGACTCAC | CGACCCTAAACCCCACTTCC |
| *AktWRKY51* | ATGATGGGTTCAGGTGGAGG | TGGGGCACTCATGGTTATGG |
| *AktWRKY53* | AATGGGAGTCCACAAAGCGA | AGCCTCTTGGGTACTTGGCT |
| *AktWRKY54* | CGGCGAGGAGGATGTAAGAG | CGCTGCACTTGTTTGATTGC |
| *AktWRKY57-1* | GCGCTCCTCTAAAGATCCCA | GGAGTTGATGGGATTGCCTG |
| *AktWRKY57-2* | ACGGGATCTGACGGAAAACC | AGCTCCTTGGGTATGGGCTA |
| *AktWRKY58* | ATGATGTACCGGCTGCAAGG | CCACCAGTGTTTGCTGCTTC |
| *AktWRKY65* | GTAGAGAGGAGCCGAGTGGA | ATTTGTGGTCCGGTTCGGTT |
| *AktWRKY68* | CCATCTCCACCATTACCCCC | ACGCAAATCTTGGCTCTCTC |
| *AktWRKY70* | CGGCTACAAAGGGAGGAGTG | AGCCTTGATCGTGCTTGTGA |
| *AktWRKY74* | AGACAGAAGCCCAAACCCAA | GGCTGAACAACCCTTTGATGT |
